# Structure-Activity Relationship of Indole-Tethered Pyrimidine Derivatives that Concurrently Inhibit Epidermal Growth Factor Receptor and Other Angiokinases

**DOI:** 10.1371/journal.pone.0138823

**Published:** 2015-09-24

**Authors:** Jiho Song, Jakyung Yoo, Ara Kwon, Doran Kim, Hong Khanh Nguyen, Bong-Yong Lee, Wonhee Suh, Kyung Hoon Min

**Affiliations:** 1 College of Pharmacy, Chung-Ang University, Seoul, Republic of Korea; 2 Life Science Research Institute, Daewoong Pharmaceutical Co., Ltd., Gyeonggi-Do, Republic of Korea; Lunenfeld-Tanenbaum Research Institute, CANADA

## Abstract

Antiangiogenic agents have been widely investigated in combination with standard chemotherapy or targeted cancer agents for better management of advanced cancers. Therapeutic agents that concurrently inhibit epidermal growth factor receptor and other angiokinases could be useful alternatives to combination therapies for epidermal growth factor receptor-dependent cancers. Here, we report the synthesis of an indole derivative of pazopanib using a bioisosteric replacement strategy, which was designated **MKP101**. **MKP101** inhibited not only the epidermal growth factor receptor with an IC_50_ value of 43 nM but also inhibited angiokinases as potently as pazopanib. In addition, **MKP101** effectively inhibited vascular endothelial growth factor-induced endothelial proliferation, tube formation, migration of human umbilical vein endothelial cells and proliferation of HCC827, an epidermal growth factor receptor-addicted cancer cell line. A docking model of **MKP101** and the kinase domain of the epidermal growth factor receptor was generated to predict its binding mode, and validated by synthesizing and evaluating **MKP101** derivatives. Additionally, a study of structure-activity relationships of indolylamino or indolyloxy pyrimidine analogues derived from **MKP101** demonstrated that selectivity for epidermal growth factor receptor and other angiokinases, especially vascular endothelial growth factor receptor 2 depends on the position of substituents on pyrimidine and the type of link between pyrimidine and the indole moiety. We believe that this study could provide a basis for developing angiokinase inhibitors having high affinity for the epidermal growth factor receptor, from the pyrimidine scaffold.

## Introduction

Angiogenesis, the formation of new blood vessels, is an essential physiological event in tumor progression [1]. Angiogenesis supplies tumors with nutrients and oxygen, thereby enabling their proliferation. Inhibition of angiogenesis has been considered a promising therapeutic strategy for suppressing tumor growth without excessive host toxicity. Over the last 2 decades, a number of antiangiogenic agents have been developed for clinical use, including monoclonal antibodies such as bevacizumab, and tyrosine kinase inhibitors (TKIs) such as sunitinib [2]. The primary molecular targets for antiangiogenic therapy include vascular endothelial growth factor receptors (VEGFRs), platelet-derived growth factor receptors (PDGFRs), and fibroblast growth factor receptors (FGFRs).

In general, multi-target agents are more effective than single-target agents for the treatment of complex diseases such as cancer [3,4]. Multi-target agents such as TKIs and aflibercept (anti-VEGF-A and -B) produced better clinical results in the regulation of tumor angiogenesis than the single-target agent bevacizumab (anti-VEGF-A) because tumors readily overcame the inhibition of angiogenesis by activating compensatory pathways such as PDGF or FGF signaling, or both [2,5]. Monotherapy with broad-spectrum angiokinase inhibitors such as sunitinib or sorafenib prolongs overall survival (OS) in some cancers [6,7,8,9], while monotherapy with bevacizumab showed unsatisfactory effect in various clinical conditions except glioblastoma [7,10,11]. However, many clinical trials have demonstrated that anti-angiogenic agents enhanced clinical efficacy when combined with conventional chemotherapy or targeted cancer agents such as erlotinib, an epidermal growth factor receptor (EGFR) TKI [12].

In phase III trials involving patients with advanced non-small cell lung cancer (NSCLC), the combination of bevacizumab and erlotinib as a second-line therapy resulted in prolonged produced progression-free survival (PFS) compared to erlotinib alone [13]. Sunitinib is an inhibitor of VEGFR1-3, PDGFRs, KIT, Fms-like tyrosine kinase 3 (FLT3), rearranged during transfection proto-oncogene (RET), and colony stimulating factor 1 receptor (CSF-1R). In another phase III trial for patients previously treated for advanced NSCLC, the combination of sunitinib and erlotinib produced a PFS that was significantly longer than that produced by erlotinib alone [14]. However, none of these combinations improved the OS in its respective phase III studies, and further investigation is required to improve OS. In a preclinical study, the combination of nintedanib (a triple angiokinase inhibitor of VEGFRs, PDGFRs, and FGFRs) and afatinib (an irreversible pan-ErbB inhibitor of EGFR, ErbB2, ErbB3, and ErbB4) potently inhibited tumor growth in HT-29 xenograft model regardless of the Kirsten rat sarcoma viral oncogene homolog (*KRAS*) status [15]. These data support the hypothesis that concurrent inhibition of key angiokinases and the EGFR could be clinically beneficial in cancer treatment. A phase II trial evaluating combination therapy with pazopanib and erlotinib is currently underway to corroborate this notion. While multi-targeted combination therapy is used for the treatment of a variety of advanced cancers, significant concerns exist regarding pharmacokinetics, dosing, and safety when designing such therapies [16]. Drug combinations are more most likely to cause unacceptable level of toxicity [17,18]. When dose-limiting toxicities occur, each component must be administered at a less than the optimal dose, which could compromise the efficacy. In such cases, monotherapy with well-designed and carefully evaluated multi-target agents would be preferable over combination therapy.

Pazopanib effectively inhibits tumor angiogenesis by potent inhibition of a variety of angiokinases including VEGFRs, PDGFRs, and FGFRs. It is currently used clinically for the treatment of advanced renal cell carcinoma and soft tissue sarcoma [19,20]. Monotherapy with pazopanib reduced tumor volume in 30 of 35 patients with stage I/II resectable NSCLC [21]. Phase II studies involving the administration of pazopanib alone and in combination with the EGFR inhibitor erlotinib are currently in progress [22]. However, we hypothesized that the modification of pazopanib to induce EGFR-inhibitory effect would lead to better treatment outcomes in patients with NSCLC and other EGFR-dependent tumors than those obtained with erlotinib or pazopanib alone. Therefore, the pazopanib derivatives could represent promising alternatives to combination therapy. Consequently, we screened derivatives of pazopanib for multi-kinase inhibitory activities that regulate both EGFR and angiokinases. To the best of our knowledge, derivatives of pazopanib that effectively inhibit EGFR have not been reported previously. Herein, we describe the synthesis and evaluation of pyrimidine derivatives that inhibit EGFR while retaining their inhibitory activity for angiokinases, and determined their structure-activity relationship (SAR).

## Materials and Methods

### Cell culture

HCC827 cells were purchased from the American Type Culture Collection (ATCC, Manassas, VA, USA). Cells were grown in RPMI 1640 (Welgene Inc., Daegu, Republic of Korea) supplemented with 10% fetal bovine serum (FBS) (Welgene Inc.) and 1% penicillin-streptomycin (Gibco®, Life Technologies, Carlsbad, CA, USA) in a humidified 5% CO_2_ atmosphere at 37°C. Human umbilical vein endothelial cells (HUVECs, ScienCell Research Laboratories, Carlsbad, CA, USA) were cultured in endothelial growth medium-2 (EGM-2; Lonza, Walkersville, MD, USA) from passages 3 to 6.

### *In vitro* kinase assay

All kinase assays were carried out using KinaseProfiler^TM^ and IC_50_ Profiler^TM^ (Millipore UK Ltd., Dundee, UK. Now Eurofins Scientific, Dundee, UK). All IC_50_ data were presented as the mean values. The Curves obtained to determine IC_50_ values were shown in Supporting Information (S1 Fig).

### Cell viability assay

HCC827 cells were seeded in 96-well plates in 100 μL of RPMI 1640 supplemented with 5% FBS and 1% penicillin-streptomycin. After a 24-hour incubation, the cells were treated with a series of test compound dilutions for 72 hours. Cell viability was assessed using EZ-Cytox (Daeil Lab Service, Seoul, South Korea) according to the manufacturer’s instructions.

For the HUVEC viability assay, HUVECs were treated with phosphate-buffered saline (PBS) or the indicated concentrations of VEGF inhibitors in EGM-2 medium for 24 hours. After the cells were washed with PBS, they were counted using an inverted light microscope (Nikon Eclipse Ti-U; Nikon Corp., Tokyo, Japan) in 5 random fields from each well.

### *In vitro* angiogenesis assay

For analyzing of the antagonistic response to VEGF, tube formation, scratch wound migration, and cell proliferation assays were performed after the exposure of HUVECs to 50 ng/mL VEGF-A (R&D Systems, Minneapolis, MN), which induced a significant angiogenic response. For the cell proliferation assays, cells were incubated overnight in endothelial basal media (EBM; Lonza, Walkersville, MD, USA) containing 0.5% FBS or supplemented with VEGF-A and/or VEGF inhibitors. The cells were washed with PBS and counted in 4 random microscope fields. The tube formation assay was performed by seeding cells on Matrigel-coated plates (BD Bioscience, Bedford, MA, USA) and incubating in EBM containing 0.5% FBS or supplemented with VEGF-A and/or VEGF inhibitors. After overnight incubation, tubule networks were quantified by measuring the tubule length in 4 random microscope fields. For the analysis of scratch wound migration, confluent cell monolayers grown on 6-well plates were scratched using a micropipette tip. After the plates were washed with PBS to remove dislodged cells and media, they were incubated with EBM containing 0.5% FBS or supplemented with VEGF and/or VEGF inhibitors for 8 hours. Cell migration was observed by optical microscopy and quantified by measuring the number of cells that had migrated from the wound edges.

### Molecular modeling

To conduct the docking studies, the crystal structure of the human EGFR kinase domain bound to TAK-285 (PDB ID: 3POZ) was prepared using the Protein Preparation Wizard [23] implemented in Maestro [24] with the following steps: (i) water molecules more than 5 Å from TAK-285 were removed; (ii) hydrogen atoms were added; (iii) protonation states of entire systems were adjusted to the pH range of 7.0 ± 3.0 using Epik; (iv) hydrogen bond networks and flip orientations/tautomeric states of Gln, Asn, and His residues were optimized; and (v) geometry optimization was performed to a maximum RMSD of 0.3 Å with the OPLS2005 force field. The protonated and neutral states of pazopanib and its derivatives were prepared using LigPrep [25]. For flexible docking, the grid box was centered on the crystal structure of TAK-285 using the default bounding sizes, with an inner box of 10 Å on each side and an outer box of 25.7 Å on each side. Glide SP (Standard-Precision) was employed for flexible docking with default parameters [26]. The best docked configurations with the lowest GlideScores were selected for analysis.

### Synthesis of MKP101-123

Synthetic procedures and spectral data of **MKP101**–**MKP123** are described in the supporting information (S1 Appendix).

## Results and Discussion

### Chemistry

**Fig 1:**
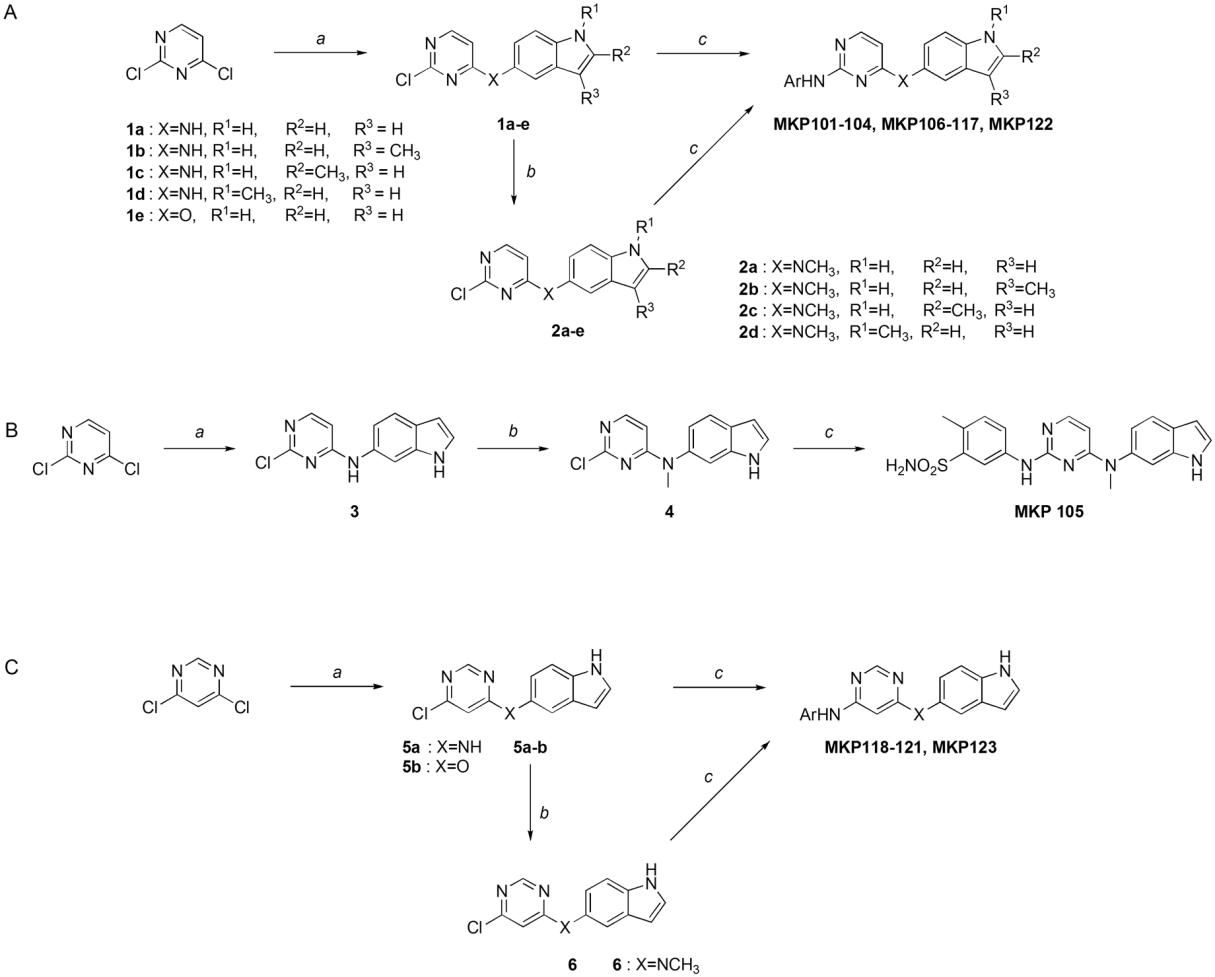
Synthesis of MKP101-123. Reagents and conditions: (A) Synthesis of **MKP101**-**104**, **MKP106**-**117**, **MKP122**: (a) 5-Aminoindoles (0.8–1.0 equiv.), Et_3_N (0.8–3.0 equiv.), isopropanol, rt, 1–10 h, or 5-hydroxyindole (0.77 equiv.), DBU (1.54 equiv.), MeCN, rt, 1 h; (b) CH_3_I (1.0–1.5 equiv.), NaH (or Cs_2_CO_3_) (1.0–1.2 equiv.), DMF, 0°C (or rt), 1 h; (c) ArNH_2_ (0.9–1.1 equiv.), 1-butanol, microwave irradiation, 200°C, 30 min. (B) Synthesis of **MKP105**: (a) 6-Aminoindole (1.5 equiv.), MeOH / H_2_O (1:3), rt, overnight; (b). CH_3_I (1.0 equiv.), NaH (1.0 equiv.), DMF, -10°C, 2h; (c) 5-Amino-2-methylbenzenesulfonamide (0.9–1.1 equiv.), 1-butanol, microwave 200°C, 30min. (C) Synthesis of **MKP118**-**121**, **MKP123**: (a) 5-Aminoindole (1.0 equiv.), Et_3_N (1.0 equiv.), isopropanol, rt, 2 h or 5-hydroxyindole (1.2 equiv.), DBU (2.0 equiv.), MeCN, rt, 1 h; (b) CH_3_I (1.1 equiv.), NaH (1.1 equiv.), DMF, 0°C rt, 1h; (c) ArNH_2_ (0.9–1.1 equiv.), 1-butanol, microwave irradiation 200°C, 30 min.

All indole-tethered pyrimidine derivatives were synthesized as shown in Fig 1. Commercially available 2,4-dichloropyrimidine was condensed with various 5-aminoindoles under basic condition to afford **1a-d**. *N*-methylation of **1a-d** using iodomethane yielded **2a-d**. Finally, condensation of intermediates **1** or **2** with a variety of anilines provided the corresponding disubstituted pyrimidines, **MKP101-104**, **106–117** and **MKP122** (Fig 1A). The synthesis of **MKP105** was accomplished by condensation of 6-aminoindole with 2,4-dichloropyrimidine and *N*-methylation, followed by reaction with 5-amino-2-methylbenzenesulfonamide (Fig 1B). **MKP118-121** and **MKP123** were prepared using a procedure similar to that used for the synthesis of **MKP106-117**. Condensation of 4,6-dichloropyrimidine with 5-aminoindole or 5-hydroxyindole yielded **5a-b**. *N*-methylation of **5a** provided compound **6**. **MKP118-121** and **MKP123** were obtained from condensation of various anilines with **5a-b**, and **6** (Fig 1C).

### MKP101 significantly inhibited EGFR, other angiokinases, and cell proliferation

**Fig 2:**
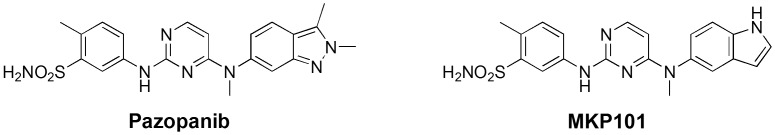
Structure of pazopanib and its indole derivative MKP101.

**Fig 3:**
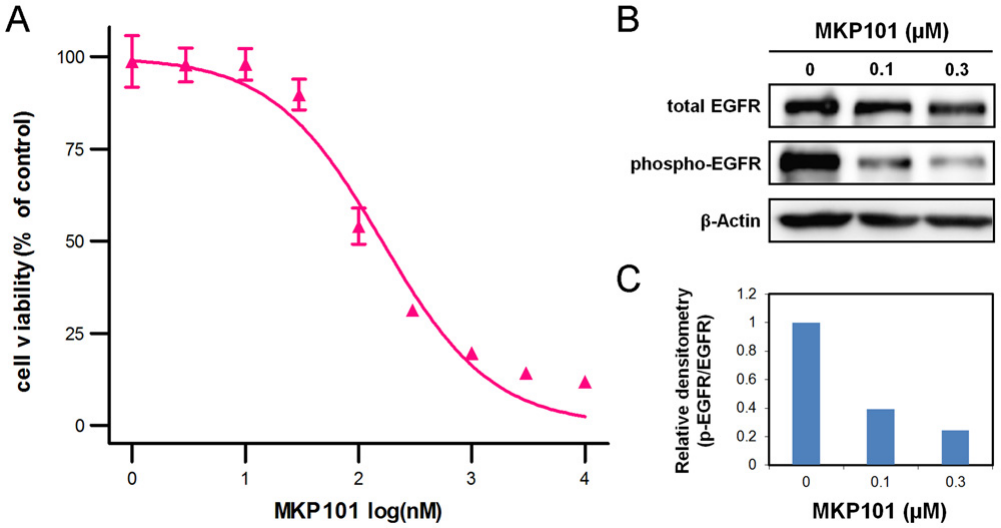
MKP101 inhibits proliferation of epidermal growth factor receptor (EGFR)-tyrosine kinase inhibitor (TKI)-sensitive non-small cell lung cancer (NSCLC) cells. (A) Inhibition of HCC827 cell proliferation. Cells were incubated for 72 h with a range of concentrations of **MKP101**, and cell viability was measured using the WST-1 assay. The IC_50_ value was 160 nM. (B) Inhibition of EGFR phosphorylation in HCC827 cells. Western blot analysis using antibodies to phosphorylated and total EGFR from a representative experiment is shown. Cells were incubated for 3 h at the indicated concentration. (C) Graph showing the relative intensities of the total and phosphorylated EGFR as determined by band densitometry. IC_50_, half-maximal inhibitory concentration.

Derivatives of pazopanib, primarily those with benzenesulfonamido moieties have been reported previously [27,28,29]. Some benzenesulfonamido pazopanib derivatives inhibited VEGF more potently than pazopanib, but did not exhibit considerable activity for EGFR [28]. Thus our derivatization strategy focused on the indazole moiety of pazopanib. The indole moiety is a viable bioisostere of indazole and is, therefore, suitable for the replacement strategy, [30]. Therefore, an indole moiety was introduced in the pazopanib molecule, and the resulting compound **MKP101** was evaluated for effects on EGFR and angiokinases (Fig 2). **MKP101** strongly inhibited wild type EGFR and the mutant EGFR L858R with IC_50_ values of 43 and 17 nM, respectively, while pazopanib did not inhibit EGFR. **MKP101** also significantly inhibited angiokinases including VEGFRs, FGFR3, PDGFRs, and cKit (Table 1). Kumar et al. provided IC_50_ values of pazopanib against 61 kinases [31], which were compared to the kinase profile data of **MKP101**. The potency of inhibition of the angiokinases by **MKP101** was similar to that induced by pazopanib in the *in vitro* kinase assay. In addition, 40 kinases were profiled to determine the selectivity of **MKP101**. Of the 40 kinases tested, only 3 kinases were inhibited by more than 90% by 1 μM **MKP101** (Table 2). Furthermore, **MKP101** was evaluated for anti-proliferative activity against HCC827 cells, an EGFR TKI-sensitive NSCLC cell line. In HCC827 cells, **MKP101** significantly inhibited proliferation with an IC_50_ value of 160 nM (Fig 3). Gefitinib was tested as a positive control, and had an IC_50_ value of 10 nM. The inhibition of EGFR phosphorylation by **MKP101** was demonstrated by western blot analysis, in which **MKP101** reduced the levels of phosphorylated EGFR.

**Table 1:** Half-maximal inhibitory concentration (IC_50_) values (nM) of MKP101 for human kinases.

Kinase	MKP101	Pazopanib*
EGFR	43	>3,000
EGFR (L858R)	17	-
FGFR1	28	80
FGFR3	43	138
VEGFR-1	3	7
VEGFR-2	32	15
VEGFR-3	2	2
PDGFRα	162	73
PDGFRβ	70	215
cKit	108	48

*The data, except for that of EGFR, are the values reported in reference [31].

**Table 2:** Kinase profile of MKP101 (1 μM) for the 40 kinases*.

Kinase	% inhibition	Kinase	% inhibition
Abl (h)	100 ± 2	MAPK2 (h)	0
AMPKα1 (h)	0	p70S6K (h)	20 ± 3
CaMKIIβ (h)	17 ± 4	PhKγ2 (h)	0 ± 4
CaMKIIγ (h)	37 ± 2	PKA (h)	7 ± 6
CaMKIIδ (h)	41 ± 3	PKBβ (h)	14 ± 4
CaMKIV (h)	0	PKCα (h)	10 ± 1
CDK1/cyclin B (h)	18 ± 8	PKCβI (h)	7 ± 5
CDK2/cyclin A (h)	12 ± 8	PKCβII (h)	8 ± 3
CDK2/cyclin E (h)	8 ± 1	PKCγ (h)	12 ± 2
CDK3/cyclin E (h)	6 ± 2	PKCδ (h)	0
CDK5/p25 (h)	47 ± 2	PKCε (h)	2 ± 3
CDK5/p35 (h)	38 ± 3	PKCη (h)	0 ± 6
CDK7/cyclin H/MAT1 (h)	16 ± 5	PKCι (h)	5 ± 6
CDK9/cyclin T1 (h)	54 ± 4	PKCμ (h)	7 ± 1
FLT3 (h)	91 ± 1	PKCθ (h)	16 ± 2
GSK3β (h)	18 ± 4	PKCζ (h)	5 ± 4
IR (h)	14 ± 3	PKG1α (h)	0 ± 1
LKB1 (h)	0	PKG1β (h)	7 ± 1
Lyn (h)	98 ± 1	ROCK-II (h)	4 ± 1
MAPK1 (h)	4 ± 5	SAPK2a (h)	82 ± 2

*data were presented as the mean ± standard deviation (S. D.).

Abl, Abelson murine leukemia; AMPK, AMP-activated protein kinase; CAMK, Ca^2+^/calmodulin-dependent protein kinase; CDK, cyclin-dependent kinase; FLT3, Fms-like tyrosine kinase 3; GSK, glycogen synthase kinase; IR, insulin receptor; LKB1, liver kinase B1; Lyn, Lck/Yes novel tyrosine kinase; MAPK, mitogen-activated protein kinases; PhK, Phosphorylase kinase; PKA, protein kinase A; PKC, protein kinase C; PKG, protein kinase G; ROCK, Rho-associated protein kinase; SAPK, stress-activated protein kinase; (h), human.

### Effect of pazopanib and MKP101 on VEGF-induced angiogenesis in endothelial cells

**Fig 4:**
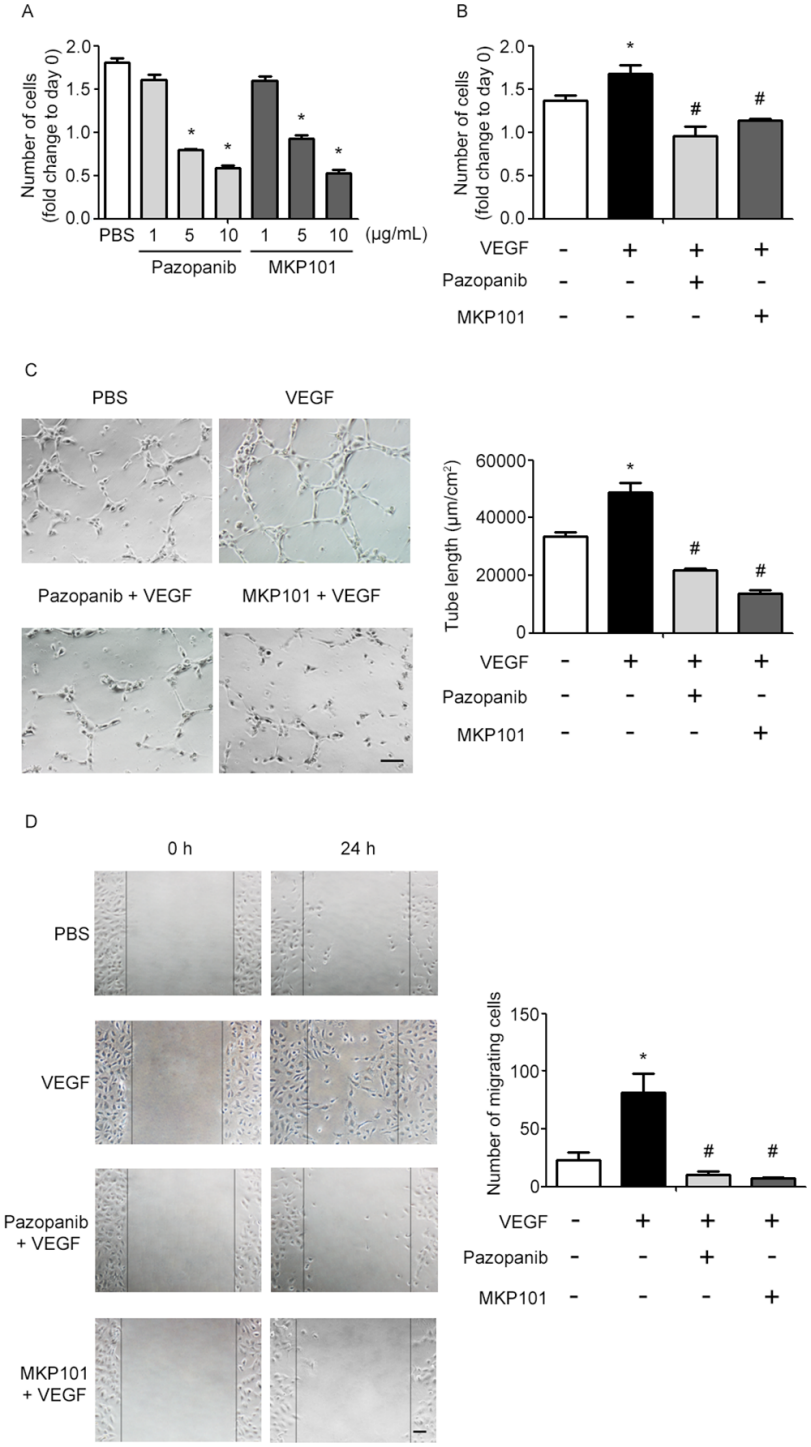
Pazopanib and MKP101 have similar inhibitory effects on VEGF-induced angiogenesis in HUVECs. (A) Cytotoxicity of pazopanib and **MKP101** in HUVECs. No cytotoxicity was observed at 1 μg/mL pazopanib or 1 μg/mL **MKP101**. (B-D) Pazopanib and **MKP101** block VEGF (50 ng/ml)-induced increases in endothelial proliferation (B), tube formation (C), and migration (D). Cell proliferation and tube formation experiments were performed using 1 μg/mL of pazopanib and 1 μg/mL **MKP101**. The scratch wound migration assay was performed using 0.5 μg/mL pazopanib and 0.5 μg/mL **MKP101**. In (A) and (B), the number of cells was expressed as the fold change with respect to the number of cells seeded at day 0. Tube formation responses were compared by normalizing the values relative to those of the corresponding PBS control samples (*p < 0.05 vs. PBS; ^#^ *p* < 0.05 vs. VEGF, mean ± SEM). Scale bar = 100 μm.

Non-cytotoxic concentrations of pazopanib and **MKP101** were tested in human umbilical vein endothelial cells (HUVECs) prior to the angiogenesis assays. Pazopanib and **MKP101** exhibited significant endothelial cytotoxicity at 5 and 10 μg/mL, but not at 1 μg/mL (Fig 4A). Therefore, *in vitro* angiogenesis assays were performed with pazopanib and **MKP101** at non-cytotoxic concentrations 1 μg/mL for both, and their inhibitory effects against VEGF-triggered angiogenesis were evaluated. As shown in Fig 4B–4D, *in vitro* angiogenesis data revealed that VEGF-induced increases in endothelial proliferation, tube formation, and migration were significantly inhibited by pazopanib and **MKP101**. **MKP101** blocked the VEGF-mediated angiogenic activity of endothelial cells with potency comparable to that of pazopanib.

### SAR and molecular docking studies

Based on the enzymatic inhibitory activity, the binding modes of pazopanib and its indole derivatives with the human EGFR kinase domain were analyzed using molecular docking studies. The indole-based **MKP101** (IC_50_, 43 nM) showed an inhibitory activity comparable to that previously reported for **TAK-285** (IC_50_, 23 nM), while the indazole-based pazopanib showed no inhibitory effect against EGFR [32]. Aertgeerts et al. previously reported that **TAK-285** competitively binds to the ATP site [32].

**Fig 5:**
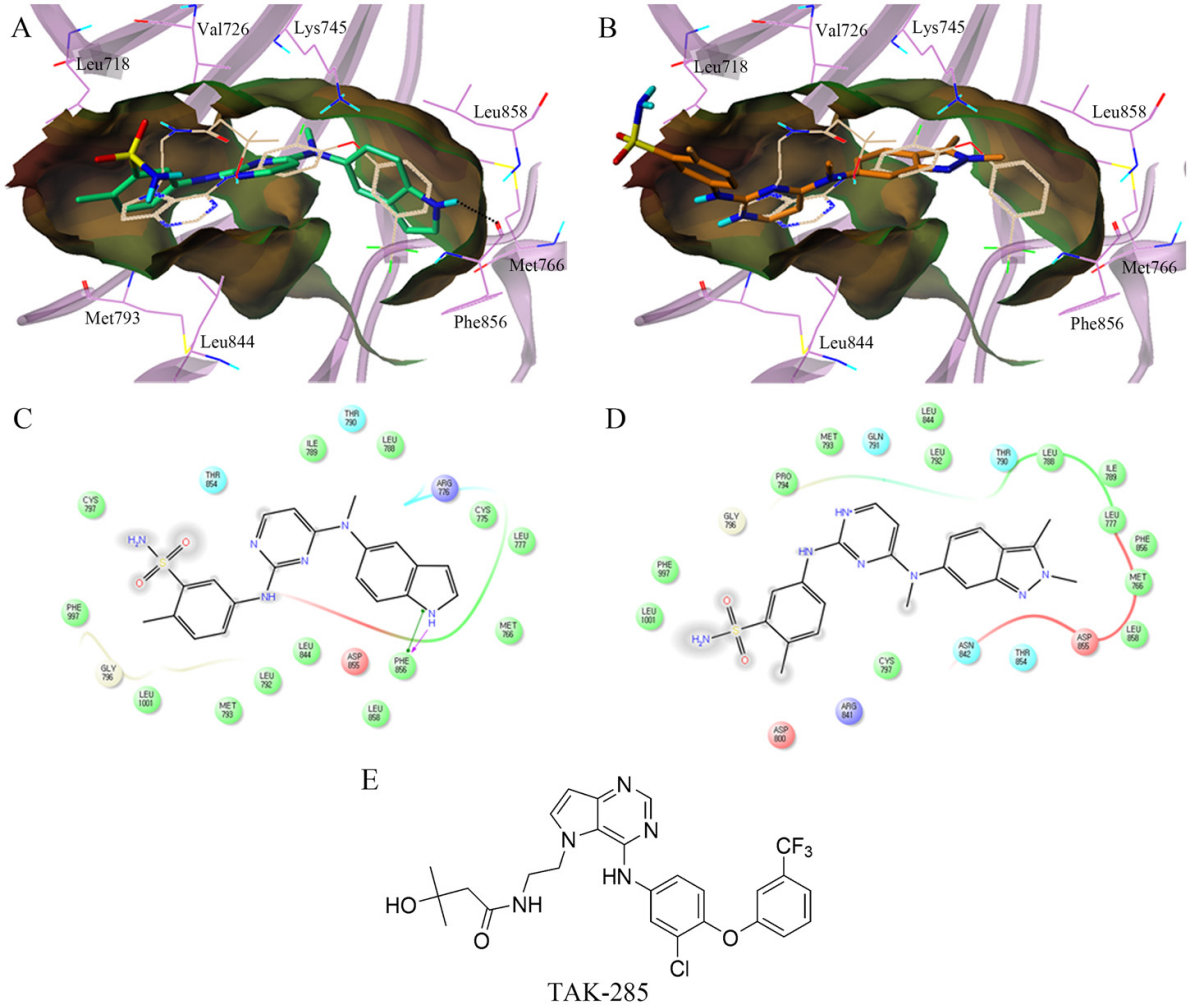
Predicted docking orientation of MKP101 in the epidermal growth factor receptor (EGFR) kinase domain. The binding poses of (A) **MKP101** (carbon atoms in green) and (B) pazopanib (carbon atoms in orange) in the human EGFR kinase domain were compared. The structure of co-crystallized TAK-285 is shown as a reference (carbon atoms in off-white). Hydrogen bonds are displayed as dashed lines. The lipophilic potential surface of the ATP-binding site of EGFR was created using the MOLCAD implemented in Sybyl-X 2.0. A 2D-interaction diagram of the binding model of (C) **MKP101** and (D) pazopanib was generated, which displayed amino acid residues within 4.0 Å of the ligand. Acidic, hydrophobic, basic, polar, and other residues at the active site are represented by red, green, purple, blue, and gray spheres, respectively. Hydrogen bonds between the ligand and the backbone are shown in dashed pink lines. The π-π stacking interaction is shown with a green line. The docking models show that **MKP101** occupies the ATP-binding site in a manner similar to **TAK-285**, and the indole ring of **MKP101** interacts with the backbone of the Phe856 by hydrogen bonding. However, as expected, pazopanib did not fit well at the ATP binding site. 2D, 2-dimensional; ATP, adenosine triphosphate.

The top-scored binding configurations and a schematic 2-dimensional (2D) representation of **MKP101** and pazopanib co-crystallized with **TAK-285** as a reference are shown in Fig 4. **MKP101** occupies the ATP binding site of the EGFR in a manner similar to **TAK-285**. The indole ring of **MKP101** occupies a lipophilic pocket formed by Met766, Cys775, Leu777, Leu788, and Phe856 in a manner similar to that observed with the 3-trifluoromethylphenyl group of **TAK-285**, and forms a direct hydrogen bond to the backbone of Phe856 (Fig 5A and 5C). The aniline moiety of **MKP101** was docked in the hinge region between the N- and C-lobes, similar to the pyrrolo[3,2-*d*]pyrimidine ring of **TAK-285**, where it participates in hydrophobic interactions with Leu718, Leu844, Leu792, and Met793 (Fig 5A and 5C). The sulfonamide group of **MKP101** was exposed to the solvent in a manner similar to that observed for hydroxymethylbutanamide moiety of **TAK-285**. Pazopanib did not fit as well as **MKP101** and **TAK-285** at the ATP binding site (Fig 5B and 5D). The rigid and bulky dimethylindazole group of pazopanib does not participate in hydrogen bond interaction with Phe856, and cannot fit into the lipophilic pocket of the EGFR. Consequently, the aniline moiety of pazopanib, which did not occupy the hinge region, was exposed to the solvent (Fig 5B and 5D).

**Fig 6:**
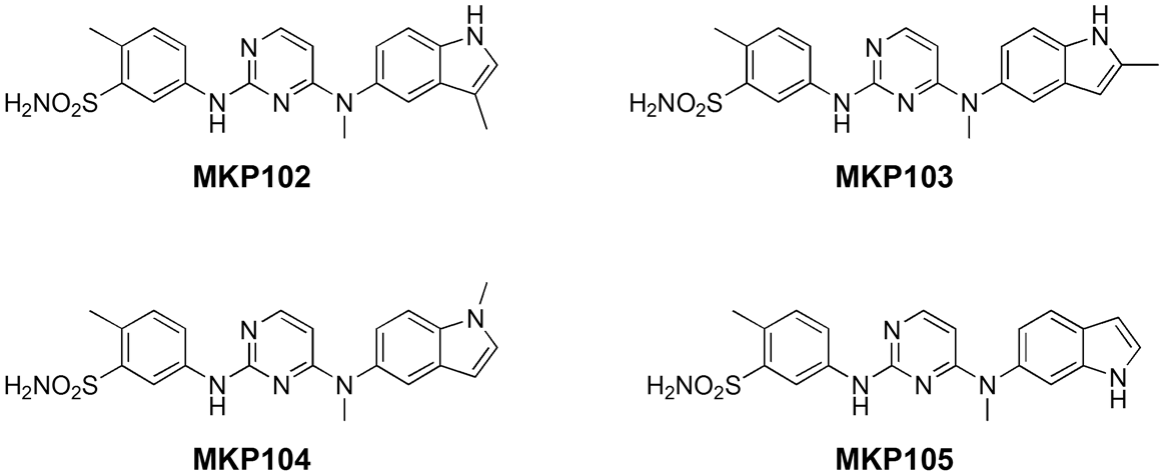
Structures of MKP102, MKP103, MKP104, and MKP105. **MKP102** and **103** are derivatives that possess a methyl group at the C-3 and C-2 positions of the indole ring, respectively. **MKP104** is a derivative with a methyl group on the indole nitrogen. **MKP105** is a regioisomer of **MKP101**.

The indole derivatives of **MKP101** (**MKP102**-**105**) were synthesized to determine the effect of the indole moiety on EGFR activity and to validate the molecular docking model of **MKP101** and EGFR. This model of EGFR-MKP101 showed that there appeared to be a space available at the C3 position of the indole moiety, but the space at the C-2 position appeared to be very limited. Therefore, we introduced a methyl group at the C-2 or C-3 position of the indole moiety, and **MKP102** (a 3-methyl indole) and **MKP103** (a 2-methyl indole) were synthesized (Fig 6). To determine whether the hydrogen bonding interaction of the amine of the indole moiety is essential, the *N*-methylated derivative **MKP104** and its regioisomer **MKP105** were synthesized. The MKP compounds (**MKP102**–**105**) were subjected to an *in vitro* kinase assay for determination of EGFR inhibitory activity (Table 3). As expected based on the docking model, only **MKP102** showed significant activity in the *in vitro* kinase assay while the other MKP compounds showed very poor activity against EGFR. In addition, the MKP compounds were evaluated for anti-proliferative activity in HCC827 cells (Table 3). Only **MKP102** displayed significant anti-proliferative activity (IC_50_, 197 nM) against HCC827 cells in a manner similar to that observed with **MKP101**. The anti-proliferative activity correlated well with the results of the kinase assay for EGFR.

**Table 3:** Activity of MKP compounds for EGFR and HCC827 cells.

	EGFR		HCC827
Compound	% inhibition at 1 μM^a^	IC_50_ (nM)	IC_50_ (nM)^c^
**MKP101**	96 ± 2	43	160
**MKP102**	93 ± 2	55	197
**MKP103**	42± 4	ND^b^	1633
**MKP104**	20 ± 4	ND	2680
**MKP105**	47± 2	ND	1465
**Gefitinib**	100 ± 0	3	10

^a^Data were presented as the mean ± standard deviation (S. D.)

^b^ND: not determined

^c^Viability experiment in the HCC827 cells was performed in triplicate and repeated at least 3 times independently. The curve obtained to determine IC_50_ values were shown in S2 Fig.

**Fig 7:**
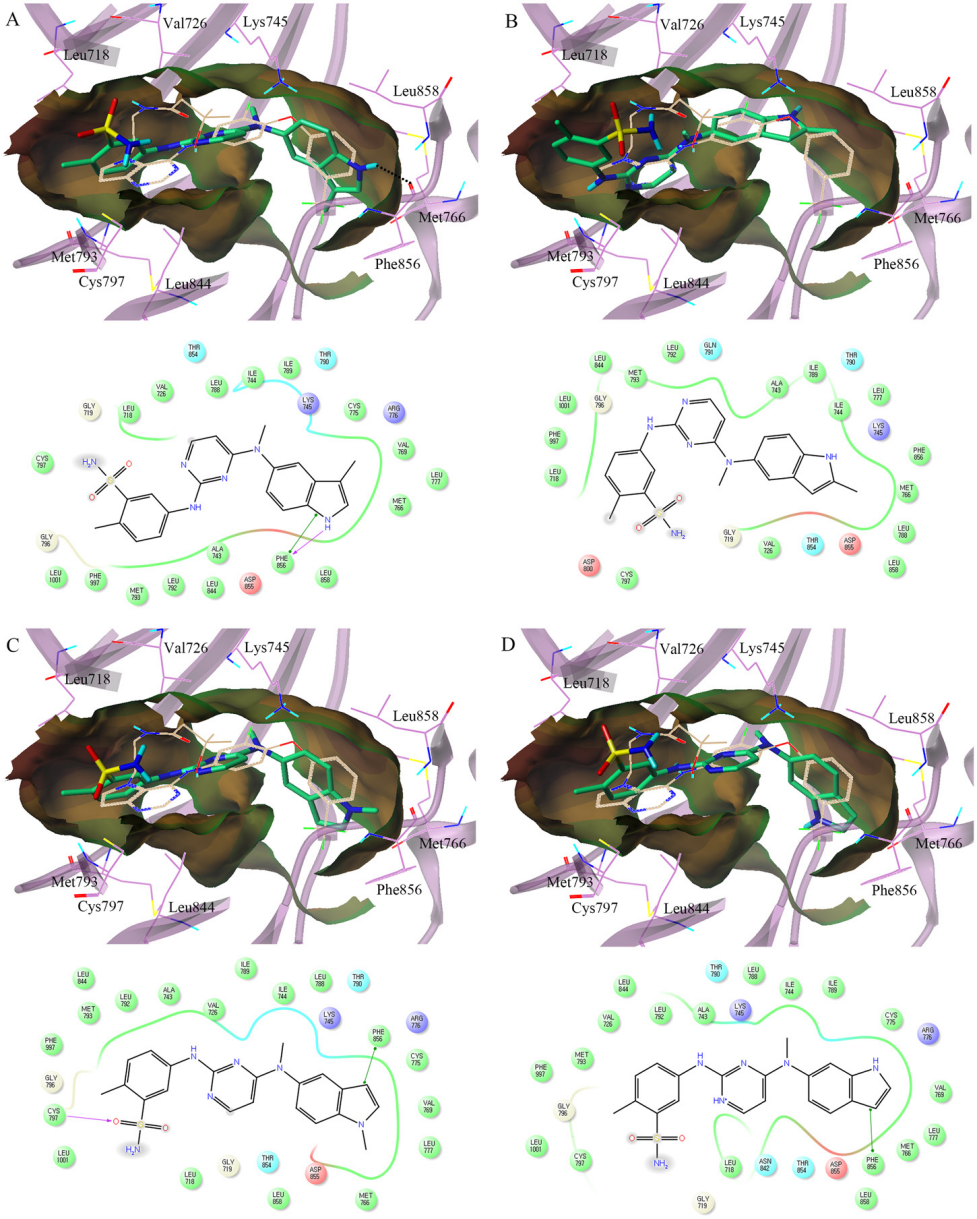
Comparison of the binding configurations of the MKP101 analogues. (A) **MKP102**, (B) **MKP103**, (C) **MKP104**, and (D) **MKP105** (carbon atoms in green) with **TAK-285** (carbon atoms in ivory). Hydrogen bonds are displayed as dashed lines. The lipophilic potential surface of the ATP-binding site of EGFR was created using the MOLCAD implemented in Sybyl-X 2.0. In the 2D-interaction diagram, acidic, hydrophobic, basic, polar, and other residues at the active site are represented by red, green, purple, blue, and gray spheres, respectively. Hydrogen bonds between the ligand and backbone are shown in dashed pink lines. The π-π stacking interaction is shown with a green line. Similar to **MKP101**, the 3-methyl indole moiety of **MKP102** occupies a lipophilic pocket that forms a direct hydrogen bond with the backbone of Phe856. However, **MKP103** the 2-methyl indole derivative, cannot fit into the lipophilic pocket because of the steric hindrance of the methyl group. The *N*-methyl indole **MKP104** and the 6-amino indole **MKP105** lost hydrogen bonding with Phe856 owing to the structural change. ATP, adenosine triphosphate; EGFR, epidermal growth factor receptor; 2D, 2-dimensional.

Further docking studies of the **MKP101** derivatives were carried out to explore their SAR. Fig 7 shows a comparison of the predicted binding mode of the analogs of **MKP101**. **MKP102** showed anti-proliferative activity comparable to that of **MKP101**, and a similar binding configuration. The 3-methyl indole amino moiety of **MKP102** occupies a lipophilic pocket that forms a direct hydrogen bond to the backbone of the Phe856 in the same orientation as the indole ring of **MKP101** (Fig 7). In contrast, **MKP103**, with its 2-methyl indole group, could not fit into the lipophilic pocket (similar to pazopanib), and showed anti-proliferative activity that was dramatically weaker than that shown by **MKP101** (Fig 7). The 5-amino-*N*-methyl indole of **MKP104** also lacked a hydrogen bonding interaction with the Phe856 at the lipophilic pocket and showed a corresponding substantial decrease in inhibitory activity (Fig 7). The binding configuration of the 6-amino indole of **MKP105** was flipped compared to the 5-amino indole analogues. As a result, **MKP105** also lost the hydrogen bond interaction with the Phe856 at the lipophilic pocket, which decreased its inhibitory activity (Fig 7). The docking results showed that a lipophilic pocket with limited space and a hinge region surrounded **MKP101** at the ATP binding site and hydrogen bonding with Phe856 at the lipophilic pocket is particularly important for potency. Taken together, the findings of the docking studies are in agreement with the enzymatic inhibitory activity assays and provide essential information for the design of a potent EGFR inhibitor.

**Fig 8:**
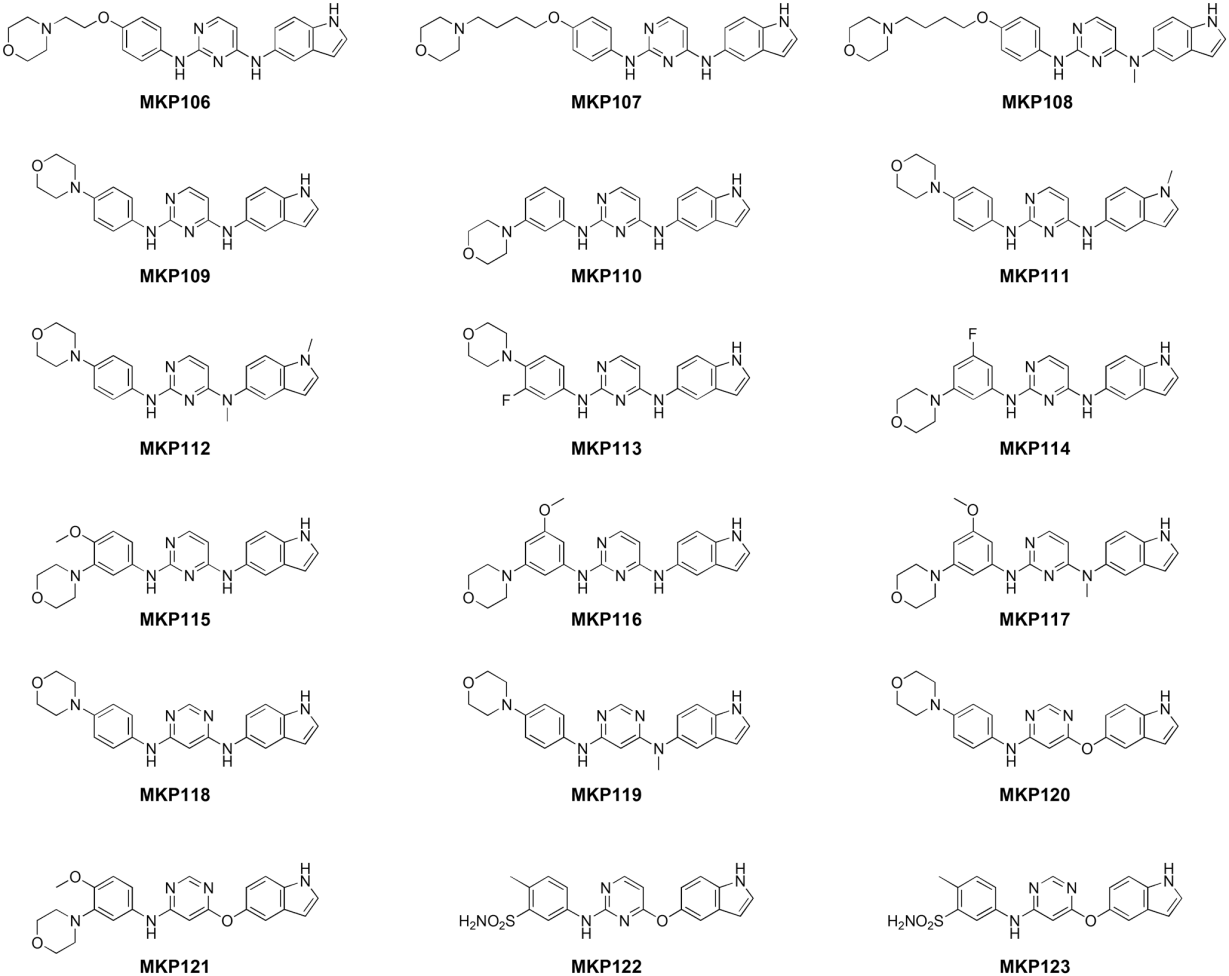
Structures of the indole tethered pyrimidine derivatives.

Next, different indole analogs for aniline moiety on the other side were synthesized to determine their effects on affinity for EGFR, based on the above results (Fig 8). The The SAR study was conducted using easily synthesized morpholine derivatives. As shown in Table 4, the synthesized compounds were evaluated for activity against EGFR and VEGFR-2. Firstly, the morpholinoethoxyaniline compound, **MKP106** showed activity against VEGFR-2 with comparable potency to **MKP101**, as well as activity against EGFR that was weaker than that of **MKP101**. Two-carbon extended compound **MKP107** retained activity for EGFR but its effect for VEGFR-2 slightly decreased, compared to **MKP106**. *N*-methylation of **MKP107** (**MKP108**) greatly improved its activity against VEGFR-2 and maintained its activity against EGFR, indicating that *N*-methylation has a positive effect on the binding with VEGFR-2 without affecting EGFR. In morpholinoaniline derivatives, *meta* substitution of morpholine (**MKP110**) led to slightly improved activity over *para* substitution (**MKP109**) against both EGFR and VEGFR. However, the introduction of fluoride, a strong electron-withdrawing group (**MKP114** and **113**) significantly decreased the activity against EGFR and retained the activity against VEGFR-2. In contrast, the introduction of an electron-donating methoxy group (**MKP116** and **115**) increased the activity against EGFR and VEGFR-2. Unlike **MKP108**, *N*-methylation of **MKP116** (**MKP117**) maintained the activity against EGFR and VEGFR-2 without significant change for VEGFR-2. *N*-methyl indole derivatives **MKP112** and **MKP111** showed poor EGFR activity, as expected from the docking study, clearly demonstrating that the NH group of indole is essential for binding to EGFR. Next, we investigated the SAR associated with changes in substitution from 2,4-disubsitututed to 4,6-disubstituted pyrimidine. **MKP118**, 4,6-dianilino derivative showed excellent activity against EGFR; however, this activity was not as significant as that of **MKP109**. The *N*-methylated compound **MKP119** also showed poor activity against VEGFR-2, but had a high affinity for EGFR. An *O*-bridge was introduced between indole and pyrimidine, replacing the *N*-bridge provided by aniline. Interestingly, the arylether **MKP120** showed excellent concurrent inhibition of EGFR and VEGFR-2 with IC_50_ values of 10 and 32 nM, respectively. **MKP121** also exhibited high activity against EGFR and VEGFR-2. These studies were also conducted with arylether derivatives of **MKP101**. The 2,4-disubstituted pyrimidine **MKP122** showed potent activity against VEGFR-2 but poor activity against EGFR. However, the 4,6-disubstituted pyrimidine **MKP123** showed potent concurrent inhibition of EGFR and VEGFR-2, with IC_50_ values of 18 and 45 nM, respectively. Taken together, these results suggest that the 4-indolyloxy-6-anilinopyrimidines could be a key structural requirement for significant inhibitory activity against EGFR and VEGFR-2. Selected compounds were screened against some angiokinases to determine the effects of structural modification on anti-angiokinase activity. The kinase profiles of these compounds are presented in Table 5. As mentioned previously, **MKP101** effectively inhibited key angiokinases. The replacement of methylated nitrogen with oxygen, as in **MKP122**, reduced activity against EGFR and FGFR. The 4-indolyloxy pyrimidines including **MKP121** and **MKP123**, which is a regioisomer of **MKP122**, retained high potency for EGFRs and VEGFRs. However, their activity against cKIT slightly decreased while that against FGFR and PDGFR markedly decreased. Differences in selectivity for angiokinases were observed between the *N*- and *O*-bridge, and the 2,4- and 4,6-disubstituted pyrimidines. To elucidate the difference, we carried out further molecular docking studies based on the docking model of EGFR-**MKP101**.

**Table 4:** Half-maximal inhibitory concentration (IC_50_) values of pyrimidine derivatives MKP106–123 against epidermal growth factor receptor (EGFR) and vascular endothelial growth factor receptor (VEGFR)-2.

	IC_50_ (nM)	
Chemicals	EGFR	VEGFR-2
**MKP106**	210	20
**MKP107**	178	130
**MKP108**	200	26
**MKP109**	240	50
**MKP110**	180	30
**MKP111**	2240	1574
**MKP112**	10120	360
**MKP113**	1630	30
**MKP114**	610	41
**MKP115**	56	10
**MKP116**	41	20
**MKP117**	33	17
**MKP118**	7	1200
**MKP119**	35	>3000
**MKP120**	10	32
**MKP121**	31	30
**MKP122**	2042	20
**MKP123**	18	45

**Table 5:** Kinase profiles of selected compounds.

	% inhibition at 1 μM^a^			
	MKP101	MKP122	MKP123	MKP121
cKit(h)	99 ± 0	99 ± 1	53 ± 9	70± 3
EGFR(h)	96 ± 2	35 ± 2	100 ± 0	94± 2
EGFR(L858R)(h)	96 ± 1	66 ± 0	99 ± 1	98± 0
FGFR1 (h)	98 ± 1	83 ± 0	20 ± 6	29 ± 5
FGFR3 (h)	96 ± 0	65 ± 4	21 ± 2	13 ± 4
VEGFR-1 (h)	99 ± 0	100 ± 0	99 ± 1	99 ± 2
VEGFR-2 (h)	94 ± 2	96 ± 0	97 ± 0	96 ± 0
VEGFR-3 (h)	98 ± 0	100 ± 0	100 ± 0	98 ± 0
PDGFRα (h)	93 ± 0	87 ± 1	30 ± 3	37 ± 1
PDGFRβ (h)	95 ± 1	92 ± 0	44 ± 3	48 ± 0

^a^Data were presented as the mean ± standard deviation (S. D.)

**Fig 9:**
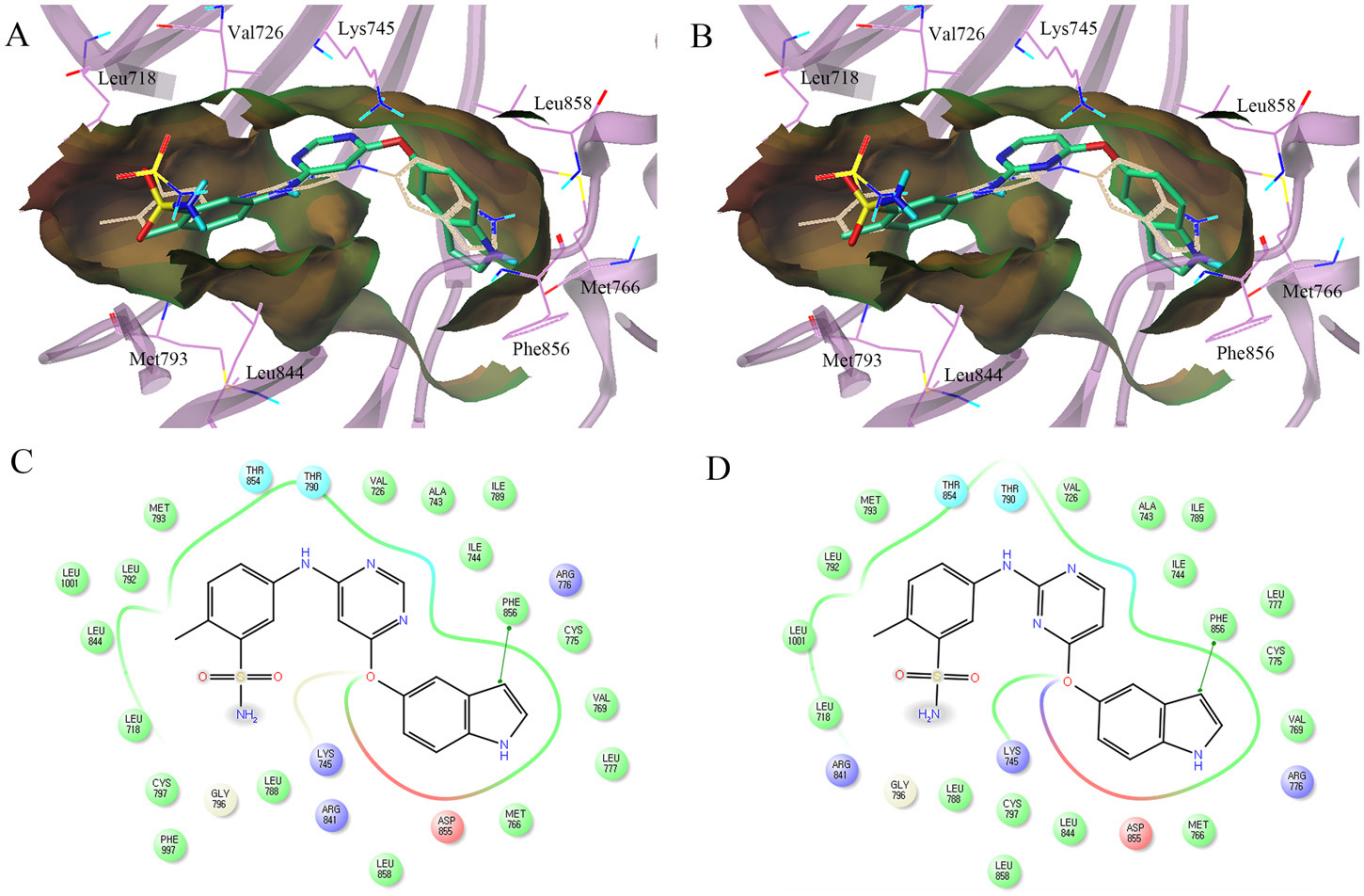
Comparison of the binding poses of MKP123 and MKP122 with MKP101. Binding poses of (A) **MKP123** (carbon atoms in green) and (B) **MKP122** (carbon atoms in green) with **MKP101** (carbon atoms in ivory). The lipophilic potential surface of the ATP-binding site of EGFR was created using the MOLCAD implemented in Sybyl-X 2.0. 2D-interaction diagram of the binding model of (C) **MKP123** and (D) **MKP122** displaying the amino acid residues within 4.0Å of the ligand. Acidic, hydrophobic, basic, polar, and other residues at the active site are represented by red, green, purple, blue, and gray spheres, respectively. The hydrogen bond between the ligand and backbone is shown in dashed pink lines. The π-π stacking interaction is shown with a green line. Unlike **MKP101**, the indole rings of **MKP123** and **MKP122** occupy a lipophilic pocket without a hydrogen bond with the backbone of Phe856, and their aniline moiety was docked in the hinge region. ATP, adenosine triphosphate; EGFR, epidermal growth factor receptor.

The binding models of **MKP122** and **MKP123** were generated and compared with the reference model, **MKP101** (Fig 9). **MKP122** and **MKP123**, which had flexible ether linkages, showed a binding position similar to **MKP101**. The indole rings of **MKP123** and **MKP122** were deeply placed into lipophilic pockets without a hydrogen bond with the backbone of Phe856 and their aniline moieties were docked in the hinge region. However, the calculated docking scores of **MKP123** were lower than those of **MKP122** (-9.6 and -8.1 kcal/mol, respectively) and therefore, more favorable. Apparently, this is owing to the difference in the electrostatic and van der Waals energies created by 6-indolyloxy-pyrimidine of **MKP123** and 4-indolyloxy pyrimidine of **MKP122**. **MKP123** showed a more favorable docking score (-9.6 kcal/mol) compared to the score that was calculated for **MKP101** (-8.9 kcal/mol). The rank order of the docking scores of **MKP123**, **MKP101**, and **MKP122** was consistent with the results of the experimental EGFR inhibition assays.

In conclusion, the indole derivative of pazopanib, **MKP101**, exhibited significant inhibitory activity against EGFR while retaining potency against other angiokinases that was equivalent to that of pazopanib. The activities of **MKP101** were demonstrated in the HUVEC cells and EGFR TKI-sensitive NSCLC cells, in addition to a kinase assay. The molecular docking study provided insight into the differences in the binding modes of pazopanib and **MKP101** to the EGFR. The docking model was validated by synthesizing **MKP101** derivatives and evaluating them for EGFR inhibitory activity and effects against EGFR-dependent cancer cells. The results of the studies of the **MKP101** derivatives supported the binding mode that was suggested by **MKP101** and EGFR. Our data suggest that further derivatization studies should be focused on determining appropriate substituents at the C-3 position of the indole or bioisosteres corresponding to 5-amino indole, which suggests that there should be a hydrogen bonding donor at the *para*-position of the amino group. Further SAR studies showed that the 2-aniline moiety of 2,4-dianilinopyrimidine did not markedly affect EGFR activity except fluoro anilines. The position of substituents on the pyrimidine ring such as 2,4- or 4,6-disubstituted pyrimidines and the type of bridge, such as a ether, secondary or tertiary amine between the indole moiety and pyrimidine significantly affected the kinase activities including those of EGFR and VEGFR-2. Multi-target drugs with low affinity could be more efficacious than potent single target drugs [3]. Therefore, we believe that further optimization of **MKP101** or its derivatives could yield a molecule that acts as a multi-targeted therapeutic agent for EGFR-dependent cancers. The development of anti-angiogenic agents that inhibit EGFR could be an alternative to combination regimens of anti-angiogenic agents and EGFR TKIs. Our findings provide a rationale for the design and development of pyrimidine-derived molecules that inhibit EGFR and other angiokinases.

## Supporting Information

S1 Appendix Synthetic procedures and characterization of MKP compounds. (PDF)

S1 Fig IC_50_ graphs of MKP compounds against various kinases. (PDF)

S2 Fig IC_50_ graphs of MKP101-105 against HCC827. (PDF)

S3 Fig NMR spectra for MKP101-123. (PDF)
