# Supplementary material for: Structure-Activity Relationship of Indole-Tethered Pyrimidine Derivatives that Concurrently Inhibit Epidermal Growth Factor Receptor and Other Angiokinases
Source: PLoS One. 2015 Sep 24;10(9):e0138823. doi: 10.1371/journal.pone.0138823 (PMC4581874; doi:10.1371/journal.pone.0138823)
Supplement: S1 Appendix — (PDF) [file pone.0138823.s001.pdf]

S1 Appendix Synthetic procedures and characterization of MKP compounds

General method Unless otherwise noted, all reagents and solvents were purchased from commercial suppliers and used without further purification. Reactions were monitored by thin-layer chromatography using Merck TLC Silica gel 60 F_{254} 250 μm plates. Flash column chromatography was performed using silica gel (ZEOCHEM, ZEOprep 60, 40-63 μm). Microwave reactions were performed using Anton Paar Monowave 300. Melting points (mp's) were determined using Büchi melting point B-540 apparatus. Infrared (IR) spectra were recorded on Thermo Scientific Nicolet 6700 with ATR sampling unit (wavenumbers in cm^{-1}). ^{1}H and ^{13}C NMR spectra were recorded on Varian Gemini 2000 (^{1}H, 300 MHz) or Varian VNS (^{1}H, 600 MHz; ^{13}C, 150 MHz). Chemical shifts were provided in units parts per million (ppm, *δ*) downfield from tetramethylsilane (TMS, internal standard) with coupling constants in hertz (Hz). All ^{13}C NMR spectra were recorded in the proton-decoupled mode. High resolution mass spectra (HRMS) were measured with Thermo Ultimate 3000/Q Exactive LC/MS system. All final compounds were purified up to >95 % purity. The purities were determined using HPLC-PDA system (pump : Waters 1525, detector : Waters 2998) equipped with C18 column (SunFire C18 5μm, 4.6 × 150 mm, Waters), using mobile phase (A) water containing 0.1 % formic acid and (B) acetonitrile. Elution condition : 0-1 min 95 % (A) and 5 %(B); 1-13 min 95-0 % (A) and 5-100 % (B); 13-15min 100 %. The flow rate was 1.2 mL/min and the injection volume was 20 μL. The system was operated at room temperature. Peaks were detected at 254 nm.

1a : *N*-(2-Chloropyrimidin-4-yl)-1*H*-indol-5-amine

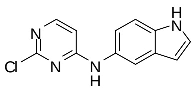

1a

A mixture of 5-aminoindole (396 mg, 3.0 mmol), 2,4-dichloropyrimidine (536 mg, 3.6 mmol), and triethylamine (418 μL, 3.0 mmol) in isopropanol (20 mL) was stirred for 1 hour at room temperature. The reaction mixture was concentrated in vacuo, and the crude product was purified by flash column chromatography (MeCN : $CHCl_3$ = 1 : 10) to afford **1a** (405 mg, 1.66 mmol, 55 %) as white solid; mp 196–198 °C; IR (ATR) *v* = 3235, 3103, 2929, 1599, 1529, 1485, 1432, 1398, 1376, 1250, 1228, 1198, 1158, 1101, 992, 876, 766, 728, 661 cm^{-1}; ^{1}H NMR (300 MHz, Acetone-*d*$_6$) : *δ* 10.34 (s, 1H), 8.81 (s, 1H), 8.04 (d, *J* = 5.7 Hz, 1H), 7.73 (s, 1H), 7.46 (d, *J* = 8.7 Hz, 1H), 7.39 (t, *J* = 3.0 Hz, 1H), 7.19 (d, *J* = 8.7 Hz, 1H), 6.61 (d, *J* = 5.7 Hz, 1H), 6.48–6.50 (m, 1H); ^{13}C NMR (150 MHz, Acetone-*d*$_6$) : *δ* 164.3, 161.3, 158.0, 135.2, 130.8, 129.4, 126.9, 118.9, 115.6, 112.7, 104.1, 102.6; HRMS (ESI): exact mass calcd for $C_{12}H_{10}ClN_4$ $[M+H]^+$, 245.0589, found 245.0594.

1b : *N*-(2-Chloropyrimidin-4-yl)-3-methyl-1*H*-indol-5-amine

1b

A solution of 3-methyl-1*H*-indol-5-amine (88 mg, 0.60 mmol) and 2,4-dichloropyrimidine (107 mg, 0.72 mmol) in 2-propanol (10 mL) were added triethylamine (84 μL, 0.60 mmol). The mixture was stirred at room temperature for 1 hour. and evaporated. The residue was purified by flash column chromatography (DCM : MeOH = 50 : 1) to give 1**b** as a white solid (97 mg, 0.375 mmol, 63 %); ^{1}H NMR (600 MHz, $CDCl_3$) : *δ* 8.07 (s, 1H), 8.02 (d, *J* = 5.9 Hz, 1H), 7.45 (s, 1H), 7.38 (d, *J* = 8.46 Hz, 1H), 7.11 (s, 1H), 7.04–7.06 (m, 2H), 6.43 (d, *J* = 5.9 Hz, 1H), 2.32 (d, *J* = 1.0 Hz, 3H); ^{13}C NMR (150 MHz, $CDCl_3$): *δ* 164.2, 160.7, 157.8, 135.0, 129.0, 128.3, 123.3, 119.8, 115.8, 112.1, 112.0, 101.6, 9.6; HRMS (ESI): exact mass calcd for $C_{13}H_{12}ClN_4$ $[M+H]^+$, 259.0745, found 259.0751.

1c : *N*-(2-Chloropyrimidin-4-yl)-2-methyl-1*H*-indol-5-amine

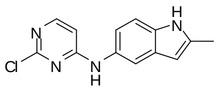

1c

A mixture of 5-amino-2-methylindole (88 mg, 0.6 mmol), 2,4-dichloropyrimidine (107 mg, 0.72 mmol), and triethylamine (84 µL, 0.6 mmol) in isopropanol (5 mL) was stirred for 1 hour at room temperature. The reaction mixture was concentrated in vacuo, and the crude product was purified by flash column chromatography (MeOH : DCM : Hexane = 1 : 20 : 5) to afford **1c** (90 mg, 0.35 mmol, 58 %) as white solid; ^{1}H NMR (600 MHz, DMSO-*d*$_6$) : *δ* 10.94 (s, 1H), 9.75 (s, 1H), 8.03 (d, *J* = 6.0 Hz, 1H), 7.49 (s, 1H), 7.26 (d, *J* = 9.0 Hz, 1H), 7.01 (s, 1H), 6.58 (s, 1H), 6.11 (s, 1H), 2.37 (s, 3H); ^{13}C NMR (150 MHz, DMSO-*d*$_6$) : *δ* 162.1, 159.5, 156.6, 136.6, 133.5, 129.3, 128.7, 115.6, 112.3, 110.6, 105.0, 99.1, 13.3; HRMS (ESI): exact mass calcd for $C_{13}H_{12}ClN_4$ [M+H]$^+$, 259.0745, found 259.0745.

1d : *N*-(2-Chloropyrimidin-4-yl)-1-methyl-1*H*-indol-5-amine

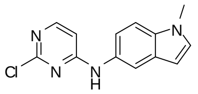

1d

A mixture of 1-methyl-1*H*-5-aminoindole (132 mg, 0.90 mmol), 2,4-dichloropyrimidine (134 mg, 0.90 mmol), and triethylamine (376 µL, 2.70 mmol) in isopropanol (9 mL) was stirred for overnight at room temperature. The resulting solid was to afford **1d** (169 mg, 0.66 mmol, 73 %) as brown solid; mp 186–188 °C; IR (ATR) *v* = 3201, 3057, 2935, 1591, 1530, 1497, 1412, 1378, 1242, 1191, 1159, 992, 807, 760, 728, 639 cm^{-1}; ^{1}H NMR (300 MHz, Acetone-*d*$_6$) : *δ* 8.84 (s, 1H), 8.04 (d, *J* = 6.0 Hz, 1H), 7.73 (s, 1H), 7.41–7.46 (m, 2H), 7.27 (d, *J* = 3.0 Hz, 1H), 6.61 (d, *J* = 6.0 Hz, 1H), 6.44 (d, *J* = 3.3 Hz, 1H), 3.85 (s, 3H); ^{13}C NMR (150 MHz, DMSO-*d*$_6$): *δ* 162.2, 159.6, 156.7, 133.9, 130.5, 130.0, 128.1, 117.0, 113.6, 110.0, 104.8, 100.3, 32.5; HRMS (ESI): exact mass calcd for $C_{13}H_{12}ClN_4$ [M+H]$^+$, 259.0745, found 259.0743.

1e : 5-(2-Chloropyrimidin-4-yloxy)-1*H*-indole

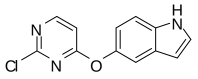

1e

A mixture of 5-Hydroxyindole (266 mg, 2.0 mmol), 2,4-dichloropyrimidine (387 mg, 2.6 mmol), and DBU (598 µL, 4.0 mmol) in Acetonitrile (3.4 mL) was stirred for 1 hour at room temperature. The mixture was diluted with EtOAc, washed with water and brine, dried over $MgSO_4$ anhydrous, and concentrated in vacuo. The crude product was purified by flash column chromatography (MeCN : $CHCl_3$ = 1 : 20) to afford **1e** (183 mg, 0.743 mmol, 37 %) as white solid; mp 173–175 °C; IR (ATR) *v* = 3236, 3109, 2919, 1575, 1548, 1489, 1438, 1345, 1227, 1211, 1190, 1128, 990, 965, 829, 759, 730, 673 cm^{-1}; ^{1}H NMR (600 MHz, Acetone-*d*$_6$) : *δ* 10.45 (s, 1H), 8.49 (d, *J* = 5.7 Hz, 1H), 7.52 (d, *J* = 8.7 Hz, 1H), 7.44 (t, *J* = 2.7 Hz, 1H), 7.42 (d, *J* = 2.4 Hz, 1H), 6.97 (dd, *J* = 8.7, 2.4 Hz, 1H), 6.90 (d, *J* = 5.7 Hz, 1H), 6.52–6.54 (m, 1H); ^{13}C NMR (150 MHz, Acetone-*d*$_6$): *δ* 172.7, 161.6, 160.9, 146.4, 135.2, 129.6, 127.5, 115.9, 113.2, 112.9, 107.4, 102.8; HRMS (ESI) : exact mass calcd for $C_{12}H_9ClN_3O$ [M+H]$^+$, 246.0429, found 246.0428.

2a : *N*-(2-Chloropyrimidin-4-yl)-*N*-methyl-1*H*-indol-5-amine

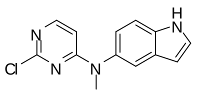

2a

To a mixture of **1a** (300 mg, 1.23 mmol) and sodium hydride (60 % dispersed in mineral oil, 59 mg, 1.47 mmol) in DMF (3 mL), was added iodomethane (115 µL, 1.84 mmol) dropwise at 0 °C. The mixture was stirred for 1 hour at 0 °C. the reaction mixture was diluted with EtOAc, washed with water and brine, dried over $MgSO_4$ anhydrous, and concentrated in vacuo. The crude product was purified by flash chromatography on silica gel (EtOAc : Hexane = 1:3) to afford **2a** (240 mg, 0.929 mmol, 76 %); ^{1}H NMR (600 MHz, $CDCl_3$) : *δ* 8.89 (s, 1H), 7.79 (d, *J* = 6.0 Hz, 1H), 7.49 (d, *J* = 8.4 Hz, 1H), 7.47 (d, *J* = 1.8 Hz, 1H), 7.33 (t, *J* = 3.0 Hz, 1H), 6.98 (dd, *J* = 8.4, 1.8 Hz, 1H), 6.58–6.59 (m, 1H), 6.08 (d, *J* = 6.0 Hz, 1H), 3.52 (s, 3H); ^{13}C NMR (150 MHz, $CDCl_3$) *δ* 164.1, 160.5, 155.4, 135.8, 135.0, 128.9, 126.1, 120.5, 118.9, 112.8, 103.6, 102.9, 38.8; HRMS (ESI) : exact mass

calcd for $C_{13}H_{12}ClN_4$ $[M+H]^+$, 259.0745, found 259.0749.

2b : *N*-(2-Chloropyrimidin-4-yl)-*N*,3-dimethyl-1*H*-indol-5-amine

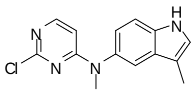

2b

A solution of **1b** (90 mg, 0.35 mmol) and cesium carbonate (114 mg, 0.35 mmol) in dry DMF (2 mL) was added iodomethane (22 µL, 0.35 mmol) in a dropwise manner and stirred at room temperature for 12 hours and filtered. The filtrate was concentrated and purified by flash chromatography on silica gel (DCM : MeCN = 20 : 1) to give **2b** as yellow oil (74 mg, 77 %); ^{1}H NMR (600 MHz, $CDCl_3$) : δ 8.47 (s, 1H), 7.78 (d, *J* = 6.1 Hz, 1H), 7.43 (d, *J* = 8.5 Hz, 1H), 7.40 (d, *J* = 1.9 Hz, 1H), 7.80 (s, 1H), 6.97 (dd, *J* = 8.5, 1.9 Hz, 1H), 6.08 (d, *J* = 6.1 Hz, 1H), 3.53 (s, 3H), 2.32 (s, 3H); ^{13}C NMR (150 MHz, $CDCl_3$) δ 164.23, 160.5, 155.5, 135.3, 135.3, 129.3, 123.5, 120.4, 117.1, 112.7, 112.1, 103.7, 38.8, 9.6; HRMS (ESI): exact mass calcd for $C_{14}H_{14}ClN_4$ $[M+H]^+$, 273.0902, found 273.0913.

2c : *N*-(2-Chloropyrimidin-4-yl)-*N*,2-dimethyl-1*H*-indol-5-amine

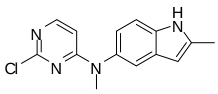

2c

To a mixture of **1c** (113 mg, 0.44 mmol), and cesium carbonate (700 mg, 2.15 mmol) in DMF (2 mL), was added iodomethane (27 µL, 0.44 mmol) at room temperature. The mixture was stirred for 1.5 hours at room temperature. The reaction mixture was diluted with EtOAc, washed with water and brine, dried over $MgSO_4$ anhydrous, and concentrated in vacuo. The crude product was purified by flash column chromatography (MeCN : $CHCl_3$ = 1 : 40) to afford **2c** (74 mg, 0.27 mmol, 62 %); IR (ATR) *v* = 3198, 2926, 1595, 1527, 1500, 1362, 1229, 1182, 981, 818, 766, 661 cm^{-1}; ^{1}H NMR (600 MHz, $CDCl_3$) : δ 8.47 (s, 1H), 7.77 (d, *J* = 6.1 Hz, 1H), 7.36 (d, *J* = 8.4 Hz, 1H), 7.32 (d, *J* = 2.1 Hz, 1H), 6.88 (dd, *J* = 8.4, 2.1 Hz, 1H), 6.24–6.25 (m, 1H), 6.06 (d, *J* = 6.1 Hz, 1H), 3.50 (s, 3H), 2.48 (s, 3H); ^{13}C NMR (150 MHz, $CDCl_3$) δ 164.2, 160.5, 155.4, 137.2, 135.7, 135.2, 130.1, 119.4, 117.7, 111.8, 103.6, 100.7, 38.8, 13.8; HRMS (ESI) : exact mass calcd for $C_{14}H_{14}ClN_4$ $[M+H]^+$, 273.0902, found 273.0900.

2d : *N*-(2-Chloropyrimidin-4-yl)-*N*,1-dimethyl-1*H*-indol-5-amine

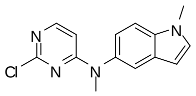

2d

To a mixture of **1a** (50 mg, 0.20 mmol) and sodium hydride (60 % dispersed in mineral oil, 41 mg, 1.02 mmol) in DMF (1 mL), was added iodomethane (38 µL, 0.61 mmol) dropwise at 0 °C. The mixture was stirred for 2 hour at room temperature. the reaction mixture was diluted with EtOAc, washed with water and brine, dried over $MgSO_4$ anhydrous, and concentrated in vacuo. The crude product was purified by flash chromatography on silica gel ($CHCl_3$: Acetonitrile = 40: 1) to afford **2d** (60 mg, 0.195 mmol, 96 %); IR (ATR) *v* = 2928, 1592, 1501, 1362, 1230, 1197, 1175, 1063, 976, 901, 811, 764, 732, 663 cm^{-1}; ^{1}H NMR (600 MHz, $CDCl_3$) : δ 7.78 (d, *J* = 6.0 Hz, 1H), 7.45 (d, *J* = 1.8 Hz, 1H), 7.40 (d, *J* = 8.4 Hz, 1H), 7.15 (d, *J* = 3.0 Hz, 1H), 7.02 (dd, *J* = 8.4, 1.8 Hz, 1H), 6.51 (dd, *J* = 3.0, 0.6 Hz, 1H), 6.04 (d, *J* = 6.0 Hz, 1H), 3.84 (s, 3H), 3.52 (s, 3H); . ^{13}C NMR (150 MHz, $CDCl_3$) δ 164.2, 160.6, 155.6, 135.8, 134.6, 130.5, 129.4, 120.2, 119.1, 110.9, 103.5, 101.4, 38.8, 33.1; HRMS (ESI) : exact mass calcd for $C_{14}H_{14}ClN_4$ $[M+H]^+$, 273.0902, found 273.0909.

MKP101 : 5-(4-((1*H*-Indol-5-yl)(methyl)amino)pyrimidin-2-ylamino)-2-methylbenzenesulfonamide

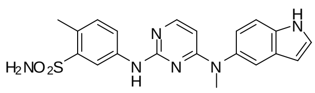
MKP101

A mixture of 5-amino-2-methylbenzenesulfonamide (14 mg, 0.077 mmol) and *N*-(2-chloropyrimidin-4-yl)-*N*-methyl-1H-indol-5-amine (**2a**) (20 mg, 0.077 mmol) in 1-butanol (1.5 mL) was stirred at 200 °C for 30 minutes using microwave reactor. The reaction vial was cooled to room temperature, and solvent was removed in vacuo. The crude product was purified by flash column chromatography (MeOH : DCM = 1 : 20) to afford MKP101 (20 mg, 0.049 mmol, 64 %); mp 142–144 °C; IR (ATR) *v* = 3365, 2929, 1600, 1577, 1550, 1519, 1427, 1404, 1365, 1317, 1235, 1161, 1065, 981, 894, 825, 797, 733, 606 cm^{-1}; ^{1}H NMR (600 MHz, CD_3OD, Acetone-d_6) : *δ* 8.64 (s, 1H), 7.67 (d, *J* = 6.0 Hz, 1H), 7.63 (dd, *J* = 8.4, 2.4 Hz, 1H), 7.48 (d, *J* = 8.4 Hz, 1H), 7.45 (d, *J* = 2.4 Hz, 1H), 7.33 (d, *J* = 2.4 Hz, 1H), 7.19 (s, 1H), 6.97 (dd, *J* = 8.4, 2.4 Hz, 1H), 6.49 (dd, *J* = 3.0, 0.6 Hz, 1H), 5.69 (d, *J* = 6.0 Hz, 1H), 3.55 (s, 3H), 2.60 (s, 3H); ^{13}C NMR (150 MHz, CD_3OD, Acetone-d_6) *δ* 163.6, 159.2, 154.0, 141.3, 138.9, 136.3, 135.1, 132.1, 128.9, 128.4, 125.9, 122.1, 120.1, 118.4, 118.0, 112.2, 101.5, 96.9, 38.2, 18.3; HRMS (ESI) : exact mass calcd for $C_{20}H_{21}N_6O_2S$ $[M+H]^+$, 409.1441, found 409.1450; Purity 99. 8 %.

MKP102 : 2-Methyl-5-(4-(methyl(3-methyl-1*H*-indol-5-yl)amino)pyrimidin-2-ylamino)benzenesulfonamide

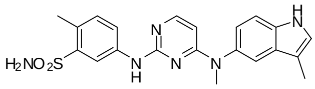
MKP102

MKP102 was prepared according to the procedure described for **MKP101**, using 5-amino-2-methylbenzenesulfonamide and *N*-(2-chloropyrimidin-4-yl)-*N*,3-dimethyl-1*H*-indol-5-amine (**2b**) (70 % yield); mp 336–338 °C; IR (ATR) *v* = 3294, 3188, 3073, 1645, 1607, 1534, 1488, 1458, 1386, 1345, 1241, 1222, 1169, 920, 820, 804, 796, 751, 666, 593 cm^{-1}; ^{1}H NMR (600 MHz, DMSO-d_6) : *δ* 11.12 (s, 1H), 11.05 (s, 1H), 8.46 (s, 1H), 7.82 (s, 1H), 7.68 (s, 1H), 7.53 (d, *J* = 2.0 Hz, 1H), 7.50 (d, *J* = 8.5 Hz, 1H), 7.43 (s, 3H), 7.26–7.27 (m, 1H), 7.05 (dd, *J* = 8.5, 2.0 Hz, 1H), 5.78 (s, 1H), 3.59 (s,z 3H), 2.58 (s, 3H), 2.26 (s, 3H); ^{13}C NMR (150 MHz, DMSO-d_6) : *δ* 162.3, 151.2, 142.6, 142.3, 135.4, 135.2, 133.3, 132.6, 131.2, 128.5, 124.7, 123.6, 119.4, 118.8, 116.0, 112.8, 110.0, 97.4, 40.0, 19.2, 9.4; HRMS (ESI): exact mass calcd for $C_{21}H_{23}N_6O_2S$ $[M+H]^+$, 423.1598, found 423.1617; Purity 99.5 %.

MKP103 : 2-Methyl-5-(4-(methyl(2-methyl-1*H*-indol-5-yl)amino)pyrimidin-2-ylamino)benzenesulfonamide

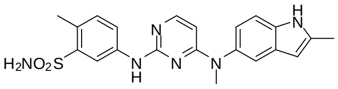
MKP103

MKP103 was prepared according to the procedure described for **MKP101**, using 5-amino-2-methylbenzenesulfonamide and *N*-(2-chloropyrimidin-4-yl)-*N*,2-dimethyl-1*H*-indol-5-amine (**2c**) (54 % yield); mp 202–204 °C; IR (ATR) *v* = 3343, 3268, 3095, 2924, 1616, 1579, 1550, 1424, 1365, 1311, 1235, 1167, 1148, 1063, 1033, 879, 797, 610 cm^{-1}; ^{1}H NMR (600 MHz, CD_3CN) : *δ* 9.24 (s, 1H), 8.58 (s, 1H), 7.88 (s, 1H), 7.73 (d, *J* = 6.0 Hz, 1H), 7.68 (dd, *J* = 8.4, 2.4 Hz, 1H), 7.38 (d, *J* = 8.4 Hz, 1H), 7.32 (d, *J* = 2.4 Hz, 1H), 7.20 (d, *J* = 8.4 Hz, 1H), 6.92 (dd, *J* = 8.4, 2.4 Hz, 1H), 6.17–6.18 (m, 1H), 5.67 (d, *J* = 6.0 Hz, 1H), 5.66 (s, 2H), 3.49 (s, 3H), 2.55 (s, 3H), 2.42 (s, 3H); ^{13}C NMR (150 MHz, CD_3CN) : *δ* 164.6, 160.4, 156.1, 142.2, 140.4, 138.5, 137.7, 136.2, 133.6, 131.0, 129.1, 123.1, 120.6, 118.7, 118.6, 112.6, 100.9, 98.2, 39.3, 19.7, 13.7; HRMS (ESI) : exact mass calcd for $C_{21}H_{23}N_6O_2S$ $[M+H]^+$, 423.1598, found 423.1621; Purity 98.0 %.

MKP104 : 2-Methyl-5-(4-(methyl(1-methyl-1*H*-indol-5-yl)amino)pyrimidin-2-ylamino)benzenesulfonamide

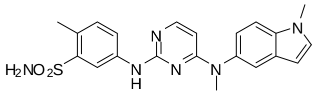
MKP104

MKP104 was prepared according to the procedure described for **MKP101**, using 5-amino-2-methylbenzenesulfonamide and *N*-(2-chloropyrimidin-4-yl)-*N*,1-dimethyl-1H-indol-5-amine (**2d**) (82 % yield); mp 274–276 °C; IR (ATR) *v* = 3334, 3261, 3090, 3036, 2942, 1617, 1618, 1582, 1549, 1525, 1420, 1364, 1330, 1309, 1257, 1237, 1167, 1136, 980, 794, 713, 610 cm^{-1}; ^{1}H NMR (600 MHz, DMSO-d_6) : *δ* 9.35 (s, 1H), 8.63 (s, 1H), 7.77 (d, *J* = 6.0 Hz, 1H), 7.73 (dd, *J* = 8.4, 2.4 Hz, 1H), 7.54 (d, *J* =

8.4 Hz, 1H), 7.49 (d, *J* = 2.4 Hz, 1H), 7.41 (d, *J* = 3.0 Hz, 1H), 7.23 (s, 2H), 7.17 (d, *J* = 7.8 Hz, 1H), 7.06 (dd, *J* = 8.4, 2.4 Hz, 1H), 6.46 (dd, *J* = 3.0, 0.6 Hz, 1H), 5.60 (d, *J* = 6.0 Hz, 1H), 3.83 (s, 3H), 3.49 (s, 3H), 2.51 (s, 3H); ^{13}C NMR (150 MHz, DMSO-d_6) : δ 163.4, 159.8, 155.8, 142.3, 139.8, 136.7, 135.6, 132.5, 131.4, 129.2, 127.5, 121.8, 120.8, 119.2, 117.7, 111.5, 101.1, 97.0, 39.0, 33.1, 19.6; HRMS (ESI) : exact mass calcd for $C_{21}H_{23}N_6O_2S$ [M+H]$^+$, 423.1598, found 423.1615; Purity 99.9 %.

MKP106 : *N*^{4}-(1*H*-Indol-5-yl)-*N*^{2}-(4-(2-morpholinoethoxy)phenyl)pyrimidine-2,4-diamine

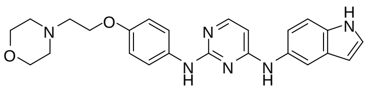

MKP 106

MKP106 was prepared according to the procedure described for **MKP101**, using 4-(2-morpholinoethoxy)aniline and *N*-(2-chloropyrimidin-4-yl)-1*H*-indol-5-amine (**1a**) (16 % yield); mp 80–82 °C; IR (ATR) *v* = 3277, 2923, 2865, 1585, 1510, 1419, 1342, 1230, 1148, 1114, 1034, 980, 953, 795, 762, 728 cm^{-1}; ^{1}H NMR (600 MHz, $CDCl_3$) : δ 8.35 (s, 1H), 7.93 (d, *J* = 5.4 Hz, 1H), 7.58 (s, 1H), 7.46 (d, *J* = 9.0 Hz, 2H), 7.37 (d, *J* = 8.4 Hz, 1H), 7.25 (t, *J* = 3.0 Hz, 1H), 7.09 (dd, *J* = 8.4, 1.8 Hz, 1H), 7.02 (s, 1H), 6.86 (d, *J* = 9.0 Hz, 2H), 6.77 (s, 1H), 6.53–6.54 (m, 1H), 6.03 (d, *J* = 5.4 Hz, 1H), 4.10 (t, *J* = 6.0 Hz, 2H), 3.74 (t, *J* = 4.8 Hz, 4H), 2.80 (t, *J* = 6.0 Hz, 1H), 2.58 (t, *J* = 4.8 Hz, 4H); ^{13}C NMR (150 MHz, $CDCl_3$) : δ 162.8, 160.2, 156.7, 154.4, 133.9, 133.2, 130.4, 128.4, 125.4, 121.9, 119.7, 116.5, 115.0, 111.6, 102.8, 95.5, 67.0, 66.1, 57.7, 54.1; HRMS (ESI) : exact mass calcd for $C_{24}H_{27}N_6O_2$ [M+H]$^+$, 431.2190, found 431.2180; Purity 99.9 %.

MKP107 : *N*^{4}-(1*H*-Indol-5-yl)-*N*^{2}-(4-(4-morpholinobutoxy)phenyl)pyrimidine-2,4-diamine

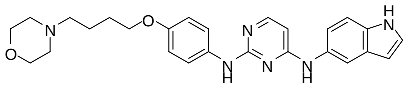

MKP 107

MKP107 was prepared according to the procedure described for **MKP101**, using 4-(4-morpholinobutoxy)aniline and *N*-(2-chloropyrimidin-4-yl)-1*H*-indol-5-amine (**1a**) (10 % yield); mp 74–76 °C; IR (ATR) *v* = 3276, 2947, 2865, 1582, 1509, 1419, 1341, 1231, 1114, 1067, 1034, 980, 795, 760, 728 cm^{-1}; ^{1}H NMR (600 MHz, $CDCl_3$) : δ 8.30 (s, 1H), 7.93(d, *J* = 6.0 Hz, 1H), 7.59 (s, 1H), 7.46 (d, *J* = 9.0 Hz, 2H), 7.38 (d, *J* = 8.4 Hz, 1H), 7.11 (dd, *J* = 8.4, 1.8 Hz, 1H), 6.91 (s, 1H), 6.85 (d, *J* = 9.0 Hz, 2H), 6.70 (s, 1H), 6.54–6.55 (m, 1H), 6.03 (d, *J* = 6.0 Hz, 1H), 3.97 (t, *J* = 6.6 Hz, 2H), 3.72 (t, *J* = 4.8 Hz, 4H), 2.33–2.47 (m, 6H), 1.78–1.83 (m, 2H), 1.65–1.70 (m, 2H); ^{13}C NMR (150 MHz, $CDCl_3$) : δ 162.8, 160.3, 156.8, 154.7, 133.8, 132.9, 130.5, 128.4, 125.4, 121.9, 119.7, 116.5, 114.8, 111.6, 102.8, 95.5, 68.0, 67.0, 58.7, 53.7, 27.3, 23.1 HRMS (ESI) : exact mass calcd for $C_{26}H_{31}N_6O_2$ [M+H]$^+$, 459.2503, found 459.2491; Purity 97.3 %.

MKP108 : *N*^{4}-(1*H*-Indol-5-yl)-*N*^{4}-methyl-*N*^{2}-(4-(4-morpholinobutoxy)phenyl)pyrimidine-2,4-diamine

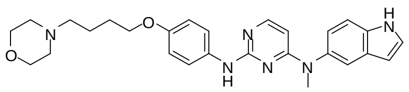

MKP 108

MKP108 was prepared according to the procedure described for **MKP101**, using 4-(4-morpholinobutoxy)aniline and *N*-(2-chloropyrimidin-4-yl)-*N*-methyl-1*H*-indol-5-amine (**2a**) (32 % yield); mp 68–70 °C; IR (ATR) *v* = 3273, 3033, 2925, 2585, 1590, 1558, 1511, 1476, 1423, 1398, 1364, 1315, 1243, 1117, 1067, 977, 866, 828, 795, 734 cm^{-1}; ^{1}H NMR (600 MHz, $CDCl_3$) : δ 8.73 (s, 1H), 7.72 (d, *J* = 6.1 Hz, 1H), 7.48–7.53 (m, 3H), 7.42 (d, *J* = 8.5 Hz, 1H), 7.21 (s, 1H), 7.02 (dd, *J* = 8.5, 2.0 Hz, 1H), 6.83 (d, *J* = 8.4 Hz, 2H), 6.54–6.47 (m, 1H), 5.68 (d, *J* = 6.1 Hz, 1H), 3.96 (t, *J* = 6.3 Hz, 2H), 3.73 (t, *J* = 4.7 Hz, 4H), 3.51 (s, 3H), 2.36–2.50 (m, 6H), 1.75–1.84 (m, 2H), 1.63–1.72 (m, 2H); ^{13}C NMR (150 MHz, $CDCl_3$) : δ 163.6, 159.6, 154.7, 154.3, 137.0, 134.6, 133.5, 128.7, 125.6, 121.4, 121.3, 119.3, 114.7, 122.2, 102.8, 96.8, 68.0, 67.0, 58.7, 53.7, 38.6, 27.3, 23.1; HRMS (ESI) : exact mass calcd for $C_{27}H_{33}N_6O_2$ [M+H]$^+$, 473.2659, found 473.2649; Purity 98.5 %.

MKP109 : N^4-(1*H*-Indol-5-yl)-N^2-(4-morpholinophenyl)pyrimidine-2,4-diamine

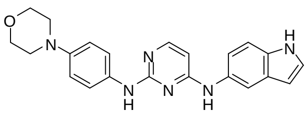

MKP 109

MKP109 was prepared according to the procedure described for **MKP101**, using 4-morpholinoaniline and *N*-(2-chloropyrimidin-4-yl)-1*H*-indol-5-amine (**1a**) (8 % yield); mp 95–97 °C; IR (ATR) v = 3278, 2961, 2857, 1595, 1516, 1419, 1343, 1229, 1116, 1067, 980, 928, 756, 761, 729, 636 cm^{-1}; ^{1}H NMR (600 MHz, $CDCl_3$) : δ 8.38 (s, 1H), 7.92 (d, *J* = 6.0 Hz, 1H), 7.58 (s, 1H), 7.47 (d, *J* = 9.0 Hz, 2H), 7.36 (d, *J* = 8.4 Hz, 1H), 7.24 (t, *J* = 3.0 Hz, 1H), 7.12 (s, 1H), 7.07 (dd, *J* = 8.4, 1.8 Hz, 1H), 6.87 (d, *J* = 9.0 Hz, 2H), 6.86 (s, 1H), 6.52–6.53 (m, 1H), 6.02 (d, *J* = 6.0 Hz, 1H), 3.86 (t, *J* = 4.8 Hz, 4H), 3.09 (t, *J* = 4.8 Hz, 4H); ^{13}C NMR (150 MHz, $CDCl_3$) : δ 162.8, 160.2, 156.6, 147.0, 133.9, 132.8, 130.4, 128.4, 125.4, 121.6, 119.7, 116.7, 116.5, 111.6, 102.8, 95.4, 67.0, 50.2; HRMS (ESI) : exact mass calcd for $C_{22}H_{23}N_6O$ $[M+H]^+$, 387.1928, found 387.1915; Purity 95.8 %.

MKP110 : N^4-(1*H*-Indol-5-yl)-N^2-(3-morpholinophenyl)pyrimidine-2,4-diamine

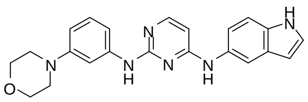

MKP 110

MKP110 was prepared according to the procedure described for **MKP101**, using 3-morpholinoaniline and *N*-(2-chloropyrimidin-4-yl)-1*H*-indol-5-amine (**1a**) (53 % yield); mp 98–100 °C; IR (ATR) v = 3392, 2923, 2857, 1595, 1496, 1424, 1341, 1251, 1200, 1067, 1033, 996, 978, 893, 798, 728, 692 cm^{-1}; ^{1}H NMR (300 MHz, CD_3OD) : δ 7.81–7.84 (m, 1H), 7.71 (s, 1H), 7.31–7.37 (m, 2H), 7.23 (t, *J* = 3.0 Hz, 1H), 7.15 (dd, *J* = 8.4, 2.4 Hz, 1H), 6.99–7.13 (m, 2H), 6.56 (dd, *J* = 8.1, 2.1 Hz, 1H), 6.38–6.40 (m, 1H), 6.09 (d, *J* = 8.7 Hz, 1H), 3.59 (t, *J* = 4.8 Hz, 4H), 2.90 (t, *J* = 4.8 Hz, 4H); ^{13}C NMR (150 MHz, $CDCl_3$) : δ 162.8, 160.1, 156.8, 151.9, 140.9, 133.9, 130.3, 129.3, 128.4, 125.5, 119.7, 116.5, 111.7, 111.6, 109.7, 107.0, 102.7, 96.0, 66.9, 49.3; HRMS (ESI) : exact mass calcd for $C_{22}H_{23}N_6O$ $[M+H]^+$, 387.1928, found 387.1916; Purity 99.9 %.

MKP111 : N^4-(1-Methyl-1*H*-indol-5-yl)-N^2-(4-morpholinophenyl)pyrimidine-2,4-diamine

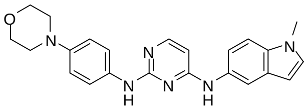

MKP 111

MKP111 was prepared according to the procedure described for **MKP101**, using 4-morpholinoaniline and *N*-(2-chloropyrimidin-4-yl)-1-methyl-1*H*-indol-5-amine (**1d**) (81 %); mp 181–183 °C; IR (ATR) v = 3245, 3098, 2954, 1656, 1606, 1526, 1450, 1329, 1308, 1254, 1225, 1111, 1069, 1049, 1023, 1005, 927, 826, 802, 758, 726 cm^{-1}; ^{1}H NMR (600 MHz, DMSO-d_6) : δ 9.86 (s, 1H), 9.46 (s, 1H), 7.95 (s, 1H), 7.89 (d, *J* = 6.0 Hz, 1H), 7.50 (d, *J* = 9.0 Hz, 2H), 7.38 (d, *J* = 8.4 Hz, 1H), 7.32 (d, 3.0 Hz, 1H), 7.25 (d, *J* = 8.4, 1.8 Hz, 1H), 6.86 (d, *J* = 9.0 Hz, 2H), 6.35 (d, *J* = 3.0 Hz, 1H), 6.25 (d, *J* = 6.0 Hz, 1H), 3.77 (s, 3H), 3.74 (t, *J* = 4.8 Hz, 4H), 3.05 (t, *J* = 4.8 Hz, 4H); ^{13}C NMR (150 MHz, DMSO-d_6) : δ 160.9, 156.9, 150.0, 146.9, 133.5, 131.3, 130.9, 130.2, 128.0, 122.0, 116.5, 115.4, 113.1, 109.6, 100.3, 97.9, 66.2, 49.1, 32.6; HRMS (ESI) : exact mass calcd for $C_{23}H_{25}N_6O$ $[M+H]^+$, 401.2084, found 401.2074; Purity 96.5 %.

MKP112 : N^4-Methyl-N^4-(1-methyl-1*H*-indol-5-yl)-N^2-(4-morpholinophenyl)pyrimidine-2,4-diamine

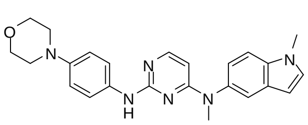

MKP 112

MKP112 was prepared according to the procedure described for **MKP101**, using 4-morpholinoaniline and *N*-(2-chloropyrimidin-4-yl)-*N*,1-dimethyl-1*H*-indol-5-amine (**2d**); mp 199–201 °C; IR (ATR) v = 3263, 3186, 2961, 1617, 1581, 1558, 1513, 1420, 1395, 1362, 1255, 1233, 1123, 977, 930, 815, 727 cm^{-1}; ^{1}H NMR (600 MHz, $CDCl_3$) : δ 7.70 (d, *J* = 6.0 Hz, 1H), 7.55 (d, *J* = 9.0 Hz, 2H), 7.47 (d, *J* = 1.8 Hz, 1H), 7.41 (s, 1H), 7.36 (d, *J* = 8.4 Hz, 1H), 7.12(d, *J* = 3.0 Hz, 1H), 7.05 (dd, *J* = 8.4, 1.8 Hz, 1H), 6.88 (d, *J* = 9.0 Hz, 2H), 6.49 (d, *J* = 3.0 Hz, 1Hz), 5.64 (d, *J* = 6.0 Hz, 1H), 3.86 (t, *J* = 4.8 Hz, 4H), 3.82 (s, 3H), 3.52 (s, 3H), 3.10 (t, *J* = 4.8 Hz, 4H); ^{13}C NMR (150 MHz, $CDCl_3$) : δ 163.5, 159.3, 154.2,

146.6, 136.7, 135.5, 133.4, 130.1, 129.2, 120.9, 120.8, 119.4, 116.6, 110.4, 101.3, 96.7, 67.0, 50.3, 38.6, 33.0; HRMS (ESI) : exact mass calcd for $C_{24}H_{27}N_6O$ $[M+H]^+$, 415.2241, found 415.2228; Purity 96.4 %.

MKP113 : *N*[2]-(3-Fluoro-4-morpholinophenyl)-*N*[4]-(1*H*-indol-5-yl)pyrimidine-2,4-diamine

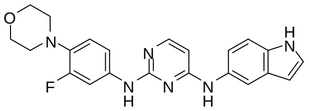

MKP 113

MKP113 was prepared according to the procedure described for **MKP101**, using 3-fluoro-4-morpholinoaniline and *N*-(2-chloropyrimidin-4-yl)-1*H*-indol-5-amine (**1a**) (48 % yield); mp 191–193 °C; IR (ATR) v = 3416, 3370, 3272, 3090, 2965, 2920, 2867, 2823, 1608, 1519, 1479, 1418, 1348, 1250, 1222, 1159, 1119, 1093, 1069, 1049, 979, 933, 925, 918, 892, 866, 800, 758, 736, 658, 626 cm^{-1}; ^1H NMR (600 MHz, $CDCl_3$) : δ 8.88 (s, 1H), 7.94 (d, J = 6.0 Hz, 1H), 7.55–7.59 (m, 3H), 7.35 (d, J = 9.0 Hz, 1H), 7.21–7.23 (m, 2H), 7.06–7.08 (m, 2H), 6.82 (t, J = 9.0 Hz, 1H), 6.50–6.51 (m, 1H), 6.05 (d, J = 6.0 Hz, 1H), 3.84 (t, J = 4.8 Hz, 4H), 3.00 (t, J = 4.8 Hz, 4H); ^{13}C NMR (150 MHz, $CDCl_3$) : δ 162.8, 159.8, 156.6, 155.7 (J_{C-F} = 243.0 Hz), 135.8 (J_{C-F} = 10.8 Hz), 134.4 (J_{C-F} = 9.4 Hz), 134.0, 130.1, 128.4, 125.6, 119.6, 118.9, 116.5, 115.2 (J_{C-F} = 2.9 Hz), 111.8, 108.4 (J_{C-F} = 25.4 Hz), 102.5, 96.0, 67.1, 51.3; HRMS (ESI) : exact mass calcd for $C_{22}H_{22}FN_6O$ $[M+H]^+$, 405.1834, found 405.1826; Purity 99.9 %.

MKP114 : *N*[2]-(3-Fluoro-5-morpholinophenyl)-*N*[4]-(1*H*-indol-5-yl)pyrimidine-2,4-diamine

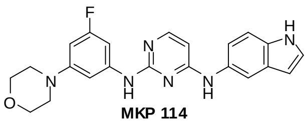

MKP 114

MKP114 was prepared according to the procedure described for **MKP101**, using 3-fluoro-5-morpholinoaniline and *N*-(2-chloropyrimidin-4-yl)-1*H*-indol-5-amine (**1a**) (49 % yield); mp 103–105 °C; IR (ATR) v = 3292, 3117, 2962, 2922, 2857, 1597, 1541, 1488, 1428, 1343, 1269, 1247, 1202, 1140, 1118, 1068, 1007, 981, 880, 800, 761, 729, 679 cm^{-1}; ^1H NMR (600 MHz, $CDCl_3$) : δ 8.31 (s, 1H), 7.96 (d, J = 5.9 Hz, 1H), 7.58 (d, J = 1.8 Hz, 1H), 7.39 (d, J = 8.5 Hz, 1H), 7.23–7.27 (m, 2H), 7.12 (dd, J = 8.5, 1.8 Hz, 2H), 6.87 (s, 1H), 6.78 (t, J = 1.8 Hz, 1H), 6.55 (ddd, J = 3.0, 2.0, 0.9 Hz, 1H), 6.24 (dt, J = 11.8, 2.2 Hz, 1H), 6.08 (d, J = 5.9 Hz, 1H), 3.78 (t, J = 4.8 Hz, 4H), 3.09 (t, J = 4.8 Hz, 4H); ^{13}C NMR (150 MHz, $CDCl_3$) : δ 164.1 (J_{C-F} = 238.3 Hz), 162.7, 159.6, 156.6, 152.8 (J_{C-F} = 11.9 Hz), 141.9 (J_{C-F} = 14.0 Hz), 134.0, 130.2, 128.4, 125.4, 119.8, 116.6, 111.7, 102.8, 101.2, 98.0 (J_{C-F} = 35.0 Hz), 96.4, 96.3 (J_{C-F} = 25.6 Hz), 66.7, 48.8; HRMS (ESI) : exact mass calcd for $C_{22}H_{22}FN_6O$ $[M+H]^+$, 405.1834, found 405.1823; Purity 98.5 %.

MKP115 : *N*[4]-(1*H*-Indol-5-yl)-*N*[2]-(4-methoxy-3-morpholinophenyl)pyrimidine-2,4-diamine

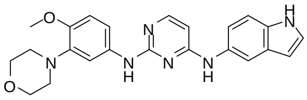

MKP 115

MKP115 was prepared according to the procedure described for **MKP101**, using 4-methoxy-3-morpholinoaniline and *N*-(2-chloropyrimidin-4-yl)-1*H*-indol-5-amine (**1a**) (69 % yield); IR (ATR) v = 3276, 2955, 2833, 1590, 1508, 1422, 1342, 1233, 1194, 1114, 1055, 1026, 979, 927, 891, 798, 758, 668 cm^{-1}; ^1H NMR (600 MHz, $CDCl_3$) : δ 8.50 (s, 1H), 7.92 (d, J = 6.0 Hz, 1H), 7.56 (d, J = 1.8 Hz, 1H), 7.34 (d, J = 8.4 Hz, 1H), 7.28 (s, 1H), 7.20–7.22 (m, 2H), 7.08 (d, J = 2.4 Hz, 1H), 7.07 (dd, J = 8.4, 1.8 Hz, 1H), 7.00 (s, 1H), 6.51–6.52 (m, 1H), 6.02 (d, J = 6.0 Hz, 1H), 3.83 (t, J = 4..8 Hz, 4H), 3.82 (s, 3H), 3.00 (t, J = 4.2 Hz, 4H); ^{13}C NMR (150 MHz, $CDCl_3$) : δ 162.9, 160.2, 156.5, 148.0, 141.2, 133.9, 133.4, 130.3, 128.4, 125.5, 119.7, 116.6, 114.9, 111.8, 111.7, 111.5, 102.7, 95.5, 67.2, 55.7, 51.0; HRMS (ESI) : exact mass calcd for $C_{23}H_{25}N_6O_2$ $[M+H]^+$, 417.2033, found 417.2020; Purity 99.4 %.

MKP116 : *N*[4]-(1*H*-Indol-5-yl)-*N*[2]-(3-methoxy-5-morpholinophenyl)pyrimidine-2,4-diamine

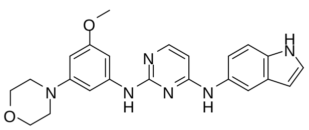

MKP 116

MKP116 was prepared according to the procedure described for **MKP101**, using 3-methoxy-5-morpholinoaniline and *N*-(2-chloropyrimidin-4-yl)-1*H*-indol-5-amine (**1a**) (65 % yield); IR (ATR) *v* = 3293, 2962, 2838, 1592, 1428, 1341, 1268, 1200, 1162, 1117, 1066, 1003, 978, 876, 799, 758 cm^{-1}; ^1H NMR (600 MHz, $CDCl_3$) *: δ* 8.54 (s, 1H), 7.94 (d, *J* = 6.0 Hz, 1H), 7.53 (d, *J* = 1.2 Hz, 1H), 7.51 (s, 1H), 7.31 (d, *J* = 8.4 Hz, 1H), 7.20 (t, *J* = 3.0 Hz, 1H), 7.18 (s, 1H), 7.05 (dd, *J* = 8.4, 1.8 Hz, 1H), 6.84 (t, *J* = 1.8 Hz, 1H), 6.73 (t, *J* = 1.8 Hz, 1H), 6.49–6,50 (m, 1H), 6.11 (t, *J* = 1.8 Hz, 1H), 6.30 (d, *J* = 6.0 Hz, 1H), 3.74 (t, *J* = 4.8 Hz, 4H), 3.70 (s, 3H), 3.04 (t, *J* = 4.8 H, 4H); ^{13}C NMR (150 MHz, $CDCl_3$) : *δ* 162.8, 160.8, 159.9, 156.5, 152.8, 141.7, 133.9, 130.2, 128.4, 125.5, 119.7, 116.5, 111.7, 102.7, 99.8, 97.2, 96.3, 96.0, 66.8, 55.2, 49.2; HRMS (ESI) : exact mass calcd for $C_{23}H_{25}N_6O_2$ $[M+H]^+$, 417.2033, found 417.2016; Purity 97.9 %.

MKP117 : *N*[4]-(1*H*-Indol-5-yl)-*N*[2]-(3-methoxy-5-morpholinophenyl)-*N*[4]-methylpyrimidine-2,4-diamine

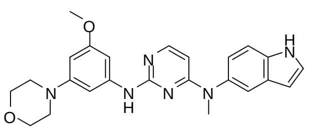

MKP 117

MKP117 was prepared according to the procedure described for **MKP101**, using 3-methoxy-5-morpholinoaniline and *N*-(2-chloropyrimidin-4-yl)-*N*-methyl-1*H*-indol-5-amine (**2a**) (67 % yield); ^1H NMR (600 MHz, $CDCl_3$) : *δ* 8.59 (s, 1H), 7.72 (d, *J* = 6.0 Hz, 1H), 7.60 (s, 1H), 7.50 (d, *J* = 1.8 Hz, 1H), 7.44 (d, *J* = 9.0 Hz, 1H), 2.8 (t, *J* = 3.0 Hz, 1H), 7.01–7.03 (m, 2H), 6.85 (t, *J* = 1.8 Hz, 1H), 6.56–6.57 (m, 1H), 6.15 (t, *J* = 1.8 Hz, 1H), 5.69 (d, *J* = 6.0 Hz, 1H), 3.83 (t, *J* = 4.8 Hz, 4H), 3.79 (s, 3H), 3.55 (s, 3H), 3.16 (t, *J* = 4.8 Hz, 4H); ^{13}C NMR (150 MHz, $CDCl_3$) : *δ* 163.5, 160.9, 159.2, 154.1, 152.8, 142.0, 136.9, 134.7, 128.7, 125.7, 121.3, 119.2, 112.4, 102.9, 99.5, 97.2, 96.7, 96.4, 66.9, 55.3, 49.5, 38.8; HRMS (ESI) : exact mass calcd for $C_{24}H_{27}N_6O_2$ $[M+H]^+$, 431.2190, found 431.2181; Purity 97.2 %.

MKP122 : 5-(4-(1*H*-Indol-5-yloxy)pyrimidin-2-ylamino)-2-methylbenzenesulfonamide

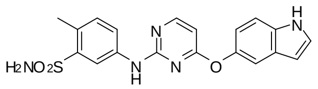

MKP 122

MKP122 was prepared according to the procedure described for **MKP101**, using 5-amino-2-methylbenzenesulfonamide and 5-(2-chloropyrimidin-4-yloxy)-1*H*-indole (**1e**) (50 % yield); IR (ATR) *v* = 3259, 3098, 2928, 1589, 1527, 1489, 1445, 1420, 1327, 1292, 1242, 1163, 1025, 1005, 950, 895, 824, 765, 733, 609 cm^{-1}; ^1H NMR (600 MHz, DMSO-d_6) : *δ* 11.23 (s, 1H), 9.69 (s, 1H), 8.30 (d, *J* = 5.7 Hz, 1H), 8.15 (s, 1H), 7.69 (dd, *J* = 8.3, 2.4 Hz, 1H), 7.47 (d, *J* = 8.6 Hz, 1H), 7.44 (t, *J* = 2.8 Hz, 1H), 7.37 (d, *J* = 2.3 Hz, 1H), 7.24 (s, 2H), 6.87–6.96 (m, 2H), 6.46 (ddd, *J* = 2.9, 1.9, 0.8 Hz, 1H), 6.29 (d, *J* = 5.7 Hz, 1H), 2.45 (s, 3H); ^{13}C NMR (150 MHz, DMSO-d_6) : *δ* 170.4, 159.6, 159.5, 145.2, 141.7, 138.2, 133.6, 131.8, 128.0, 127.8, 126.7, 121.7, 117.8, 115.3, 112.1, 112.0, 101.2, 98.1, 19.0; HRMS (ESI) : exact mass calcd for $C_{19}H_{18}N_5O_3S$ $[M+H]^+$, 396.1125, found 396.1115; Purity 99.9 %.

3 : *N*-(2-Chloropyrimidin-4-yl)-1*H*-indol-6-amine [1]

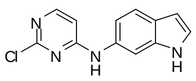

3

A mixture of 6-aminoindole (297 mg, 2.25 mmol), 2,4-dichloropyrimidine (218 mg, 1.46 mmol), methanol (1 mL), and water (3 mL) was stirred for 2 hours at room temperature. The reaction mixture was diluted with water, acidified with 2N-HCl, and sonicated. The resulting solid was filtered, washed with water and dried to give **3** (247 mg, 67 %) as white solid; mp 223–225 °C; ^1H NMR (300 MHz, DMSO-d_6) : *δ* 11.12 (s, 1H), 9.93 (s, 1H), 8.08 (d, *J* = 6.0 Hz, 1H), 7.77 (br s, 1H), 7.51 (d, *J* = 8.4 Hz, 1H), 7.31 (t, *J* = 2.7 Hz, 1H), 7.03 (d, *J* = 8.4 Hz, 1H), 6.70 (d, *J* = 6.0 Hz, 1H), 6.40 (s, 1H); ^{13}C NMR (150 MHz, DMSO-d_6) : *δ* 161.8, 159.6, 135.9, 132.2, 125.5, 124.6, 120.2, 113.9, 105.4, 104.2, 101.1; HRMS (ESI): exact mass calcd for $C_{12}H_{10}ClN_4$ $[M+H]^+$, 245.0589, found

245.0594.

4 : *N*-(2-Chloropyrimidin-4-yl)-*N*-methyl-1*H*-indol-6-amine

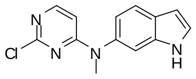

4

To a mixture of **3** (50 mg, 0.2 mmol) and sodium hydride (60 % dispersed in mineral oil, 8 mg, 0.2 mmol) in DMF (1 mL), was added iodomethane (13 μL, 0.2 mmol) dropwise at -10 °C. The mixture was stirred for 2 hour at -10 °C. the reaction mixture was diluted with EtOAc, washed with water and brine, dried over $MgSO_4$ anhydrous, and concentrated in vacuo. The crude product was purified by flash chromatography on silica gel (EtOAc : Hexane = 1:2) to afford **4** (42 mg, 0.162 mmol, 81 %); ^{1}H NMR (600 MHz, $CDCl_3$) : *δ* 8.77 (s, 1H), 7.79 (d, *J* = 6.0 Hz, 1H), 7.71 (d, *J* = 7.8 Hz, 1H), 7.33 (dd, *J* = 3.2, 2.5 Hz, 1H), 7.23–7.24 (m, 1H), 6.92 (dd, *J* = 8.4, 1.8 Hz, 1H), 6.60–6.61 (m, 1H), 6.12 (d, *J* = 6.0 Hz, 1H), 3.52 (s, 3H); ^{13}C NMR (150 MHz, $CDCl_3$) *δ* 164.0, 160.5, 155.6, 137.8, 136.2, 127.5, 126.0, 122.4, 118.5, 109.5, 103.7, 102.7, 38.7; HRMS (ESI) : exact mass calcd for $C_{13}H_{12}ClN_4$ $[M+H]^+$, 259.0745, found 259.0748.

MKP105 : 5-(4-((1*H*-Indol-6-yl)(methyl)amino)pyrimidin-2-ylamino)-2-methylbenzenesulfonamide

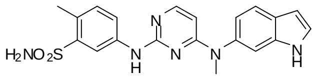

MKP 105

MKP105 was prepared according to the procedure described for **MKP101**, using 5-amino-2-methylbenzenesulfonamide and *N*-(2-chloropyrimidin-4-yl)-*N*-methyl-1*H*-indol-6-amine. (**4**) (58 % yield); mp 139–141 °C; IR (ATR) *v* = 3354, 2931, 1579, 1549, 1510, 1426, 1404, 1318, 1253, 1234, 1160, 1063, 981, 934, 896, 797, 766, 730, 661, 606 cm^{-1}; ^{1}H NMR (600 MHz, CD_3OD) : *δ* 8.61 (s, 1H), 7.68 (d, *J* = 6.0 Hz, 1H), 7.63 (d, *J* = 8.4 Hz, 1H), 7.59 (dd, *J* = 8.4, 2.4 Hz, 1H), 7.31 (d, *J* = 3.0 Hz, 1H), 7.28–7.29 (m, 1H), 7.17 (d, *J* = 8.4 Hz, 1H), 6.89 (dd, *J* = 8.4, 1.8 Hz, 1H), 6.50 (dd, *J* = 3.0, 0.6 Hz, 1H), 5.74 (d, *J* = 6.0 Hz, 1H), 3.56 (s, 3H), 2.59 (s, 3H); ^{13}C NMR (150 MHz, CD_3OD) : *δ* 165.0, 160.6, 155.4, 142.7, 140.3, 139.7, 138.1, 133.5, 130.0, 128.8, 127.2, 123.6, 122.5, 119.5, 119.3, 111.0, 102.6, 98.3, 39.6, 19.7; HRMS (ESI) : exact mass calcd for $C_{20}H_{21}N_6O_2S$ $[M+H]^+$, 409.1458, found 409.1457; Purity 97.5 %.

5a : *N*-(6-Chloropyrimidin-4-yl)-1*H*-indol-5-amine

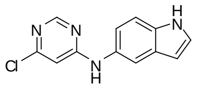

5a

A mixture of 4,6-dichloropyrimidine (500 mg, 3.36 mmol), 5-aminoindole (444 mg, 3.36 mmol), and trimethylamine (468 μL, 3.36 mmol), in isopropanol (10 mL) was stirred for 2 hours at room temperature. The mixture was diluted with EtOAc, washed by water and brine, dried over $MgSO_4$ anhydrous, and concentrated in vacuo. The crude product was purified by flash column chromatography (MeOH : DCM = 1 : 40) to afford **5a** (800 mg, 3.27 mmol, 97 %) as white solid; mp 232–234 °C; IR (ATR) *v* = 3224, 3086, 2991, 1512, 1524, 1484, 1461, 1442, 1394, 1344, 1314, 1225, 1155, 1098, 998, 762, 745, 728, 663 cm^{-1}; ^{1}H NMR (600 MHz, DMSO-*d_6*) : *δ* 11.12 (s, 1H), 9.69 (s, 1H), 8.39 (d, *J* = 0.6 Hz, 1H), 7.74 (s, 1H), 7.41 (d, *J* = 8.4 Hz, 1H), 7.37 (t, *J* = 2.6 Hz, 1H), 7.14 (d, *J* = 6.0 Hz, 1H), 6.64 (s, 1H), 6.44 (t, *J* = 2.1 Hz, 1H); ^{13}C NMR (150 MHz, DMSO-*d_6*) : *δ* 162.2, 158.6, 157.8, 133.3, 130.0, 127.8, 126.2, 117.0, 113.6, 111.7, 103.4, 101.2; HRMS (ESI): exact mass calcd for $C_{12}H_{10}ClN_4$ $[M+H]^+$, 245.0589, found 245.0589.

5b : 5-(6-Chloropyrimidin-4-yloxy)-1*H*-indole

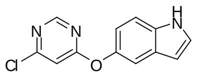

5b

A mixture of 4,6-dichloropyrimidine (536 mg, 3.6 mmol), 5-hydroxyindole (400 mg, 3.0 mmol), and 1,8-Diazabicyclo[5.4.0]undec-7-ene (897 μL, 6.0 mmol) in acetonitrile (5 mL) was stirred for 1 hour

at room temperature. The mixture was diluted with EtOAc, washed by water and brine, dried over $MgSO_4$ anhydrous, and concentrated in vacuo. The crude product was purified by flash column chromatography (MeOH : DCM = 1 : 40) to afford **5b** (684 mg, 2.78 mmol, 93 %) as white solid; mp 140–142 °C; IR (ATR) *v* = 3362, 2924, 2856, 1568, 1541, 1483, 1456, 1382, 1135, 1210, 1085, 986, 970, 881, 845, 804, 756, 723, 627 cm^{-1}; ^1H NMR (600 MHz, $CDCl_3$) : δ 8.60 (d, *J* = 0.6 Hz, 1H), 8.54 (s, 1H), 7.40 (d, *J* = 8.7 Hz, 1H), 7.39 (d, *J* = 2.4 Hz, 1H), 7.25 (t, *J* = 3.0 Hz, 1H), 6.94 (dd, *J* = 8.7, 2.4 Hz, 1H), 6.84 (d, *J* = 0.6 Hz, 1H), 6.55–6.56 (m, 1H); ^1H NMR (600 MHz, $CDCl_3$) : δ 171.5, 161.9, 158.6, 145.6, 133.9, 128.5, 126.1, 115.5, 112.5, 112.2, 107.3, 103.0; HRMS (ESI) : exact mass calcd for $C_{12}H_9ClN_3O$ $[M+H]^+$, 246.0429, found 246.0428.

6 : *N*-(6-Chloropyrimidin-4-yl)-*N*-methyl-1*H*-indol-5-amine

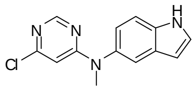

6

To a mixture of **5a** (122 mg, 0.5 mmol) and sodium hydride (60 % dispersed in mineral oil, 22 mg, 0.55 mmol) in DMF (1.5 mL), was added iodomethane (34 μL, 0.55 mmol) dropwise at 0 °C. The mixture was stirred for 5 hour at 0 °C. the reaction mixture was diluted with DCM, washed with water and brine, dried over $MgSO_4$ anhydrous, and concentrated in vacuo. The crude product was purified by flash chromatography on silica gel (EtOAc : Hexane = 1:2) to afford **6** (82 mg, 0.317 mmol, 63 %); ^1H NMR (600 MHz, $CDCl_3$) δ 9.13 (s, 1H), 8.48 (s, 1H), 7.48–7.50 (m, 2H), 7.32 (t, *J* = 3.0 Hz, 1H), 6.99 (dd, *J* = 8.6, 2.0 Hz, 1H), 6.59 (ddd, *J* = 3.0, 2.0, 0.8 Hz, 1H), 6.21 (s, 1H), 3.53 (s, 3H); ^{13}C NMR (150 MHz, $CDCl_3$) δ 163.8, 158.6, 158.0, 135.7, 135.0, 129.0, 126.1, 120.5, 118.9, 1113.0, 103.4, 102.8, 39.0; HRMS (ESI) : exact mass calcd for $C_{13}H_{12}ClN_4$ $[M+H]^+$, 259.0745, found 259.0743.

MKP118 : N^4-(1*H*-Indol-5-yl)-N^6-(4-morpholinophenyl)pyrimidine-4,6-diamine

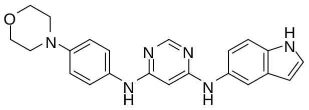

MKP 118

MKP118 was prepared according to the procedure described for **MKP101**, using 4-morpholinoaniline and *N*-(6-chloropyrimidin-4-yl)-1*H*-indol-5-amine (**5a**) (22 % yield); mp 299-301 °C; IR (ATR) *v* = 3340, 3234, 2854, 1736, 1596, 1555, 1512, 1480, 1439, 1331, 1307, 1271, 1204, 1116, 1066, 986, 922, 825, 763, 734 cm^{-1}; ^1H NMR (300 MHz, DMSO-d_6) δ : 11.00 (s, 1H), 8.72 (s, 2H), 8.13 (s, 1H), 7.61 (d, *J* = 1.5 Hz, 1H), 7.28–7.37 (m, 4H), 7.06 (dd, *J* = 8.6, 1.9 Hz. 1H), 6.88 (d, *J* = 9.0 Hz, 2H), 6.35–6.40 (m, 1H), 5.92 (s, 1H), 3.73 (t, *J* = 4.8 Hz, 4H), 3.02 (t, *J* = 4.8 Hz, 4H); ^{13}C NMR (150 MHz, DMSO-d_6) δ : 161.6, 160.9, 157.7, 146.5, 132.8, 132.7, 131.6, 127.8, 125.8, 121.6, 117.3, 115.8, 113.1, 111.4, 100.9, 83.4, 66.1, 49.2; HRMS (ESI) : exact mass calcd for $C_{22}H_{23}N_6O$ $[M+H]^+$, 387.1928, found 387.1912; Purity 99.9 %.

MKP119 : N^4-(1*H*-Indol-5-yl)-N^4-methyl-N^6-(4-morpholinophenyl)pyrimidine-4,6-diamine

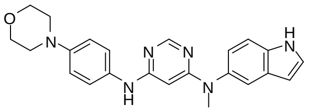

MKP 119

MKP119 was prepared according to the procedure described for **MKP101**, using 4-morpholinoaniline and *N*-(6-chloropyrimidin-4-yl)-*N*-methyl-1*H*-indol-5-amine (**6**) (51 % yield); mp 177–179 °C; IR (ATR) *v* = 3236, 3081, 2954, 1596, 1560, 1516, 1491, 1448, 1403, 1275, 1230, 1178, 1117, 985, 926, 820, 772, 741 cm^{-1}; ^1H NMR (600 MHz, $CDCl_3$) : δ 8.43 (s, 1H), 8.31 (d, *J* = 0.9 Hz, 1H), 7.48 (d, *J* = 2.0 Hz, 1H), 7.39 (d, *J* = 8.5 Hz, 1H), 7.24 (t, *J* = 3.0 Hz, 1H), 7.06 (d, *J* = 9.0 Hz, 2H), 7.02 (dd, *J* = 8.5, 2.0 Hz, 1H), 6.75 (d, *J* = 9.0 Hz, 2H), 6.59 (s, 1H), 6.53–6.55 (m, 1H), 5.56 (d, *J* = 1.0 Hz, 1H), 3.82 (t, *J* = 4.8 Hz, 4H), 3.48 (s, 3H), 3.02 (t, *J* = 4.8 Hz, 4H); ^{13}C NMR (150 MHz, $CDCl_3$) : δ 163.7, 160.3, 157.7, 147.9, 137.5, 134.4, 131.6, 128.7, 125.4, 123.1, 121.3, 119.0, 116.6, 112.1, 102.9, 83.6, 66.9, 49.8, 38.7; HRMS (ESI) : exact mass calcd for $C_{23}H_{25}N_6O$ $[M+H]^+$, 402.2084, found 402.2075; Purity 99.9 %.

MKP120 : 6-(1*H*-Indol-5-yloxy)-*N*-(4-morpholinophenyl)pyrimidin-4-amine

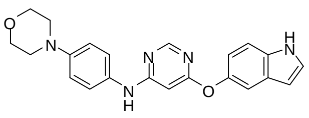
MKP 120

MKP120 was prepared according to the procedure described for **MKP101**, using 4-morpholinoaniline and 5-(6-chloropyrimidin-4-yloxy)-1*H*-indole (**5b**) (35 % yield); mp 78–80 °C; IR (ATR) *v* = 3255, 2963, 2856, 1594, 1515, 1456, 1419, 1342, 1293, 1263, 1169, 1120, 1049, 1024, 1002, 982, 927, 821, 761, 731 cm^{-1}; ^{1}H NMR (600 MHz, $CDCl_3$) : *δ* 8.72 (s, 1H), 8.30 (d, *J* = 0.6 Hz, 1H), 7.53 (s, 1H), 7.34 (d, *J* = 2.4 Hz, 1H), 7.31 (d, *J* = 8.7 Hz, 1H), 7.19 (d, *J* = 9.0 Hz, 2H), 7.17 (t, *J* = 3.0 Hz, 1H), 6.91 (dd, *J* = 8.7, 2.4 Hz, 1H), 6.87 (d, *J* = 9.0 Hz, 2H), 6.48–6.49 (m, 1H), 6.03 (d, *J* = 0.6 Hz, 1H), 3.85 (t, *J* = 4.8 Hz, 4H), 3.10 (t, *J* = 4.8 Hz, 4H); ^{13}C NMR (150 MHz, $CDCl_3$) : *δ* 171.3, 163.7, 158.5, 148.9, 146.3, 133.6, 130.4, 128.4, 125.7, 124.4, 116.5, 116.0, 112.5, 111.8, 102.7, 86.6, 66.8, 49.5; HRMS (ESI) : exact mass calcd for $C_{22}H_{22}N_5O_2$ $[M+H]^+$, 388.1768, found 388.1758; Purity 98.8 %.

MKP121 : 6-(1*H*-Indol-5-yloxy)-*N*-(4-methoxy-3-morpholinophenyl)pyrimidin-4-amine

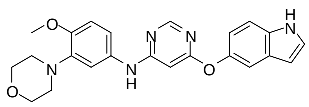
MKP 121

MKP121 was prepared according to the procedure described for **MKP101**, using 4-methoxy-3-morpholinoaniline and 5-(6-chloropyrimidin-4-yloxy)-1*H*-indole (**5b**) (67 % yield); mp 147–149 °C; IR (ATR) *v* = 3235, 3055, 2966, 2865, 2835, 1607, 1579, 1508, 1457, 1410, 1252, 1199, 1179, 1134, 1108, 1055, 1015, 986, 889, 869, 830, 762, 731 cm^{-1}; ^{1}H NMR (600 MHz, $CDCl_3$) *: δ* 8.35 (s, 1H), 8.35 (d, *J* = 0.6 Hz, 1H), 7.37 (d, *J* = 9.0 Hz, 1H), 7.36 (d, *J* = 2.4 Hz, 1H), 7.23 (t, *J* = 3.0 Hz, 1H), 6.95 (dd, *J* = 9.0, 2.4 Hz, 1H), 6.94 (s, 1H), 6.85 (dd, *J* = 8.4, 2.4 Hz, 1H), 6.80 (d, *J* = 8.4 Hz, 1H), 6.73 (d, *J* = 2.4 Hz, 1H), 6.51–6.52 (m, 1H), 6.00 (d, *J* = 0.6 Hz, 1H), 3.82–3.84 (m, 7H), 2.88 (t, *J* = 4.5 Hz, 4H); ^{13}C NMR (150 MHz, $CDCl_3$) *: δ* 171.8, 163.6, 158.8, 149.9, 146.4, 141.8, 133.6, 131.1, 128.5, 125.7, 117.8, 116.2, 113.7, 113.6, 112.8, 112.0, 103.1, 86.0, 67.1, 55.7, 50.9; HRMS (ESI) : exact mass calcd for $C_{23}H_{24}N_5O_3$$[M+H]^+$, 418.1874, found 418.1863; Purity 95.6 %.

MKP123 : 5-(6-(1*H*-Indol-5-yloxy)pyrimidin-4-ylamino)-2-methylbenzenesulfonamide

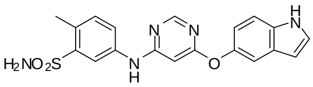
MKP 123

MKP123 was prepared according to the procedure described for **MKP101**, using 5-amino-2-methylbenzenesulfonamide and 5-(6-chloropyrimidin-4-yloxy)-1*H*-indole (**5b**) (51 % yield); mp 113–115 °C; IR (ATR) *v* = 3360, 2927, 2853, 1592, 1470, 1412, 1303, 1257, 1213, 1161, 1064, 1011, 987, 952, 895, 829, 763, 730, 606 cm^{-1}; ^{1}H NMR (300 MHz, CD_3OD) : *δ* 8.31 (d, *J* = 0.9 Hz, 1H), 8.20 (d, *J* = 2.4 Hz, 1H), 7.61 (dd, *J* = 8.2, 2.4 Hz, 1H), 7.46 (dt, *J* = 8.7, 0.7 Hz, 1H), 7.30–7.35 (m, 2H), 7.21–7.27 (m, 1H), 6.91 (dd, *J* = 8.7, 2.3 Hz, 1H), 6.48 (dd, *J* = 3.1, 0.9 Hz, 1H), 5.90 (d, *J* = 0.9 Hz, 1H), 2.58 (s, 3H); ^{13}C NMR (150 MHz, DMSO-d_6) : *δ* 170.8, 162.2, 158.1, 145.4, 142.1, 138.0, 133.7, 132.5, 128.8, 128.2, 126.9, 122.3, 118.1, 115.2, 112.4, 112.0, 101.4, 88.6, 19.1; HRMS (ESI) : exact mass calcd for $C_{19}H_{18}N_5O_3S$ $[M+H]^+$, 396.1125, found 396.1119; Purity 99.9 %.
