# Supplementary material for: Structure-Activity Relationship of Indole-Tethered Pyrimidine Derivatives that Concurrently Inhibit Epidermal Growth Factor Receptor and Other Angiokinases
Source: PLoS One. 2015 Sep 24;10(9):e0138823. doi: 10.1371/journal.pone.0138823 (PMC4581874; doi:10.1371/journal.pone.0138823)

S1 Fig. IC_{50} graphs of MKP compounds against various kinases.

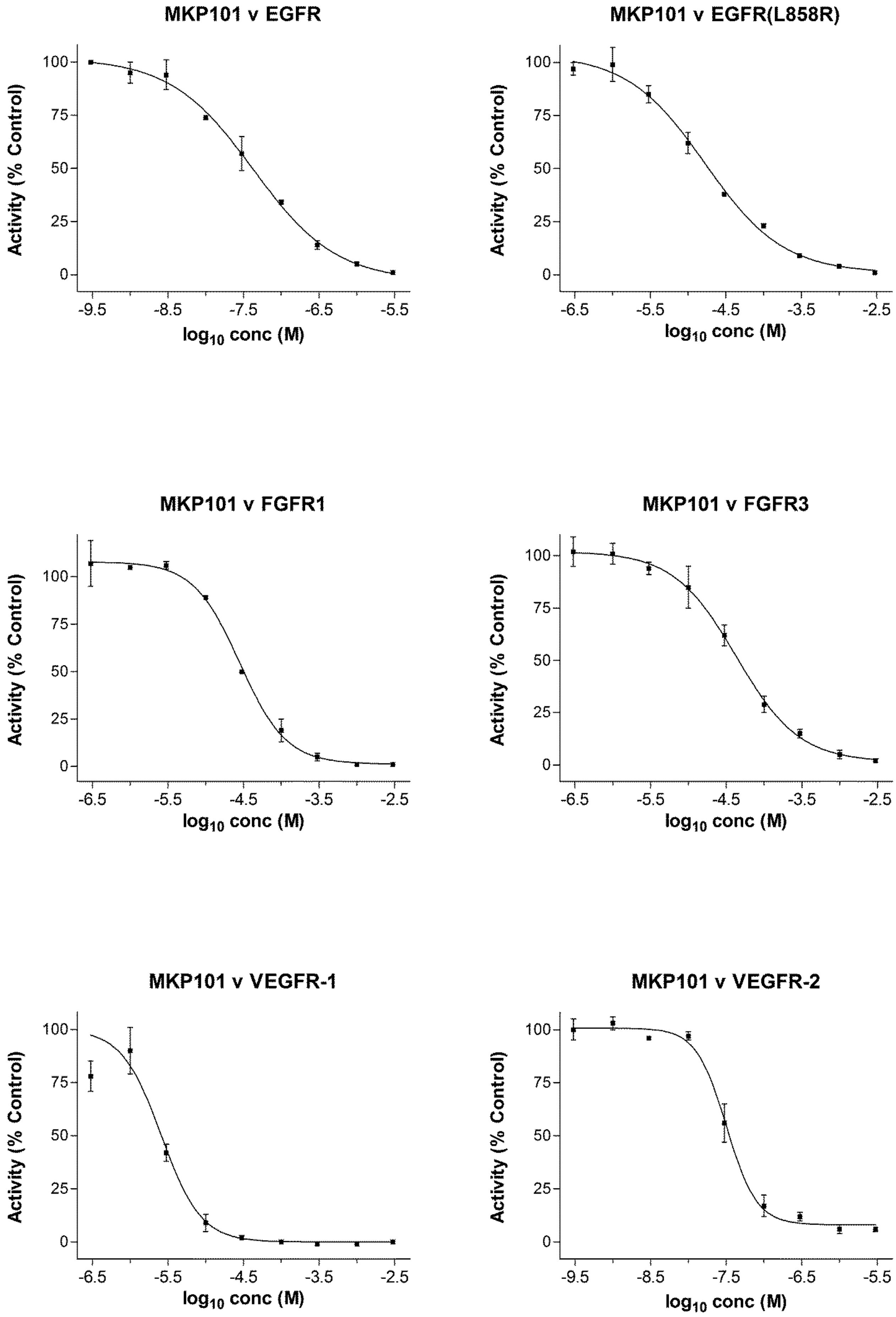

MKP101 v VEGFR-3

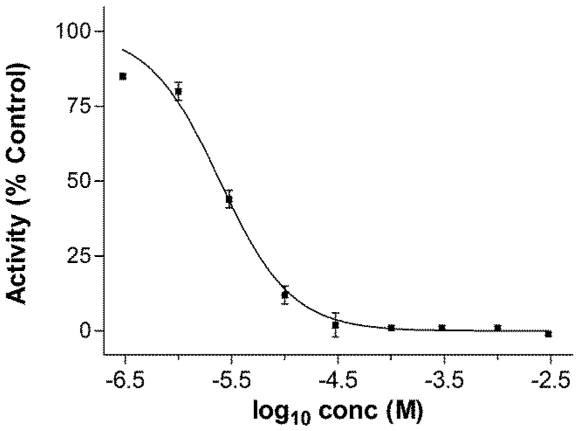

MKP101 v PDGFRα

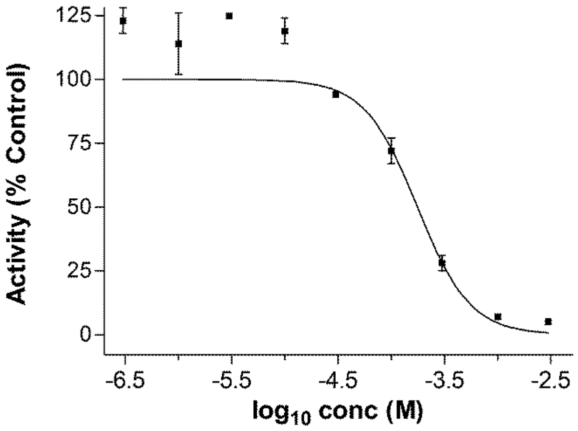

MKP101 v PDGFRβ

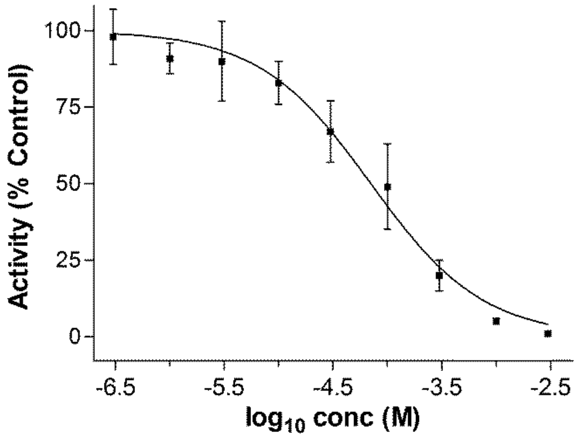

MKP101 v cKit

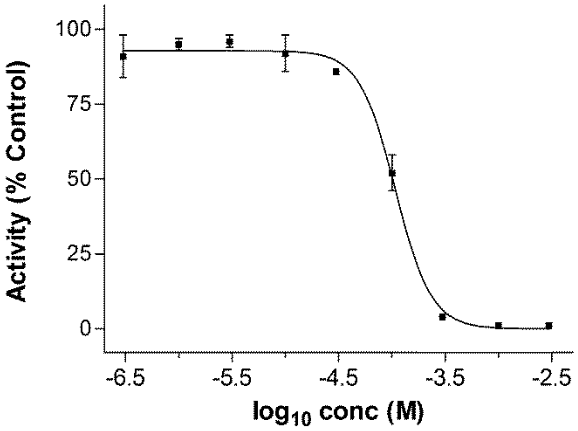

Pazopanib v EGFR

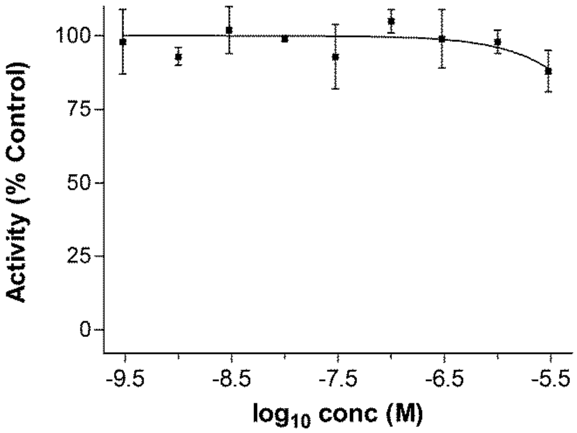

Pazopanib v VEGFR-2

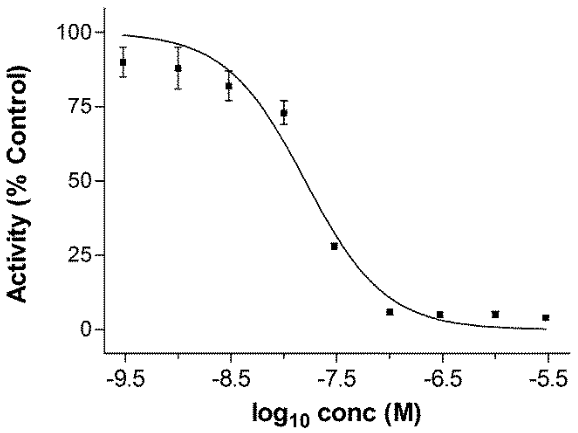

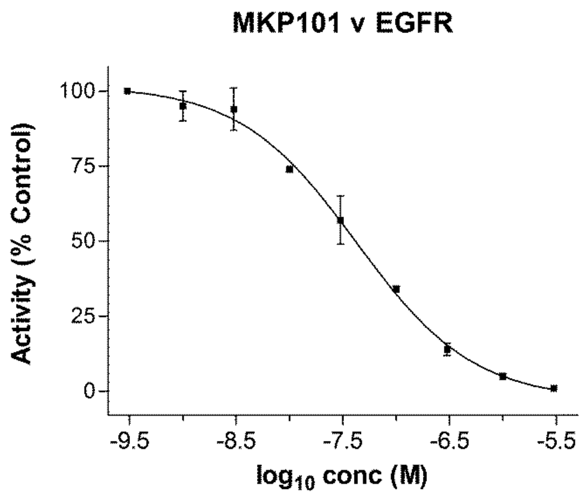
MKP101 v EGFR
Activity (% Control)
100
75
50
25
0
-9.5
-8.5
-7.5
-6.5
-5.5
log10 conc (M)

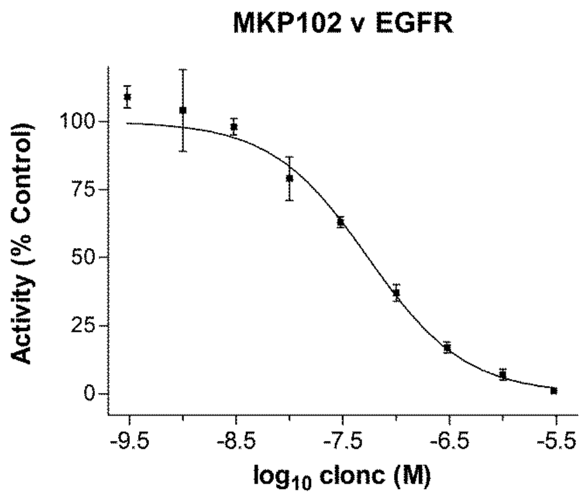
MKP102 v EGFR
Activity (% Control)
100
75
50
25
0
-9.5
-8.5
-7.5
-6.5
-5.5
log10 clonc (M)

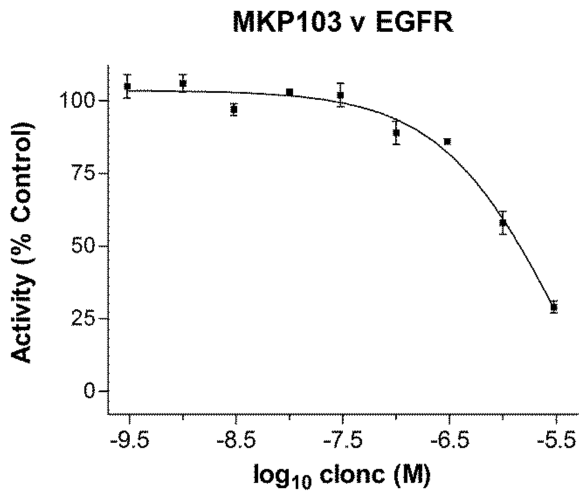
MKP103 v EGFR
Activity (% Control)
100
75
50
25
0
-9.5
-8.5
-7.5
-6.5
-5.5
log10 clonc (M)

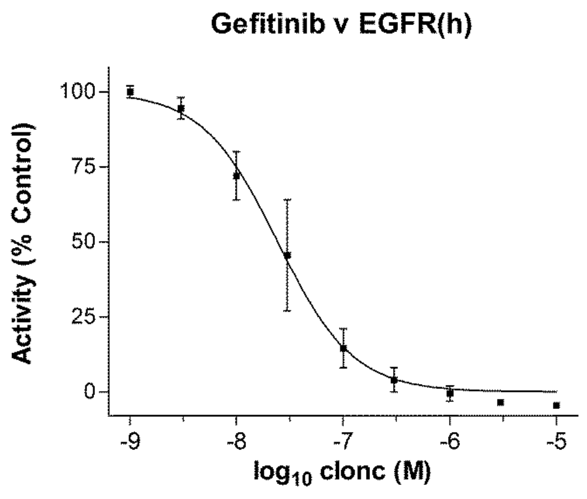
Gefitinib v EGFR(h)
Activity (% Control)
100
75
50
25
0
-9
-8
-7
-6
-5
log10 clonc (M)

MKP106 v EGFR

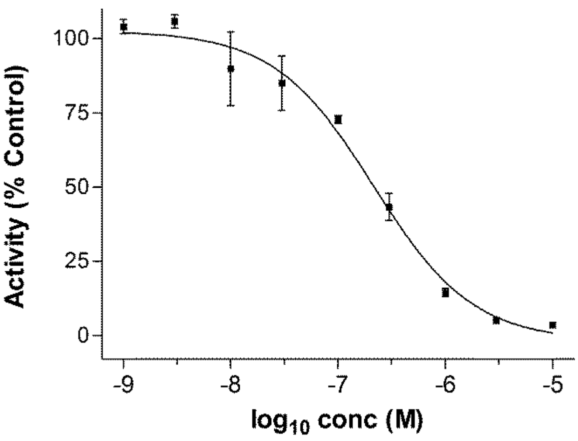

MKP106 v VEGFR-2

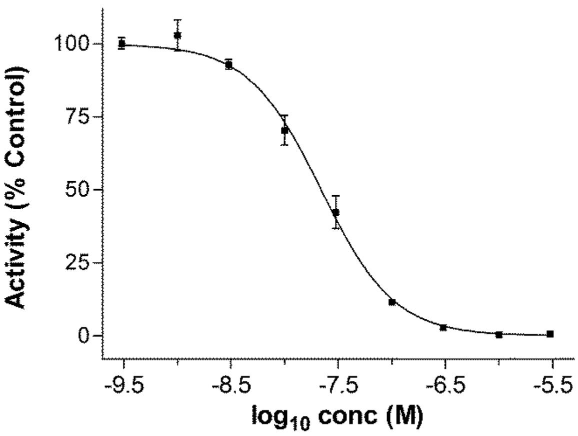

MKP107 v EGFR

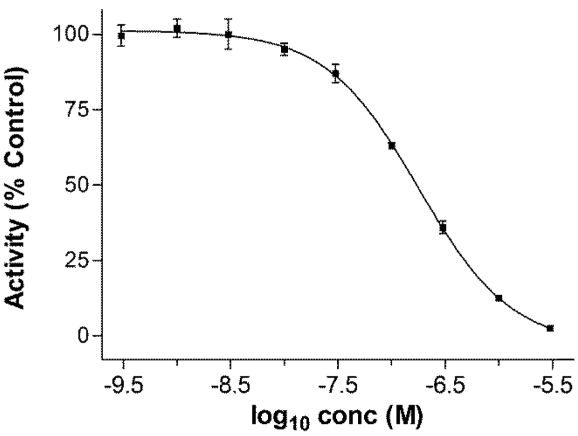

MKP107 v VEGFR-2

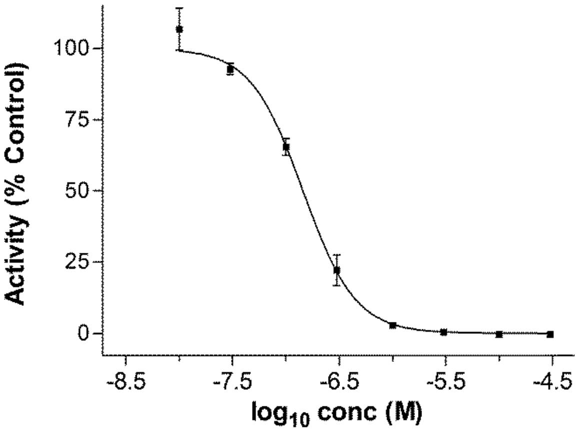

MKP108 v EGFR

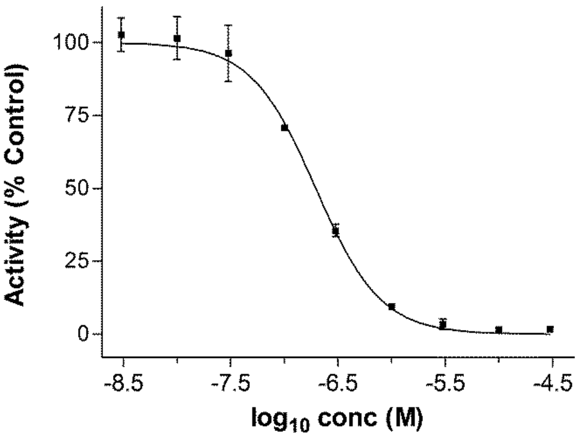

MKP108 v VEGFR-2

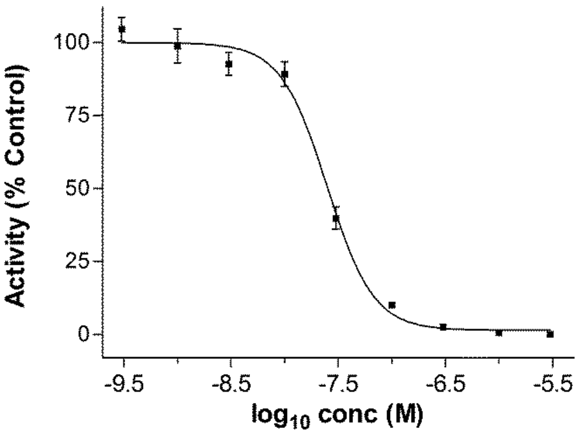

MKP109 v EGFR

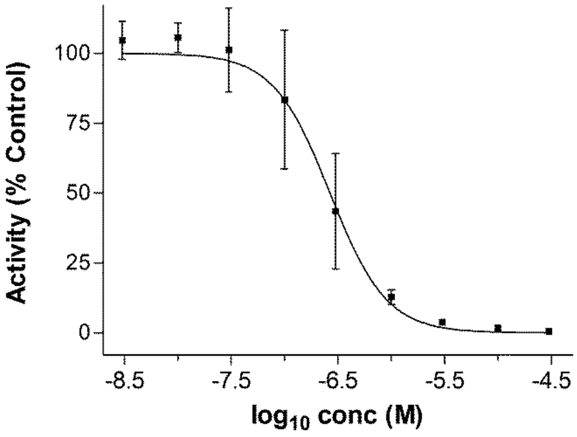

MKP109 v VEGFR-2

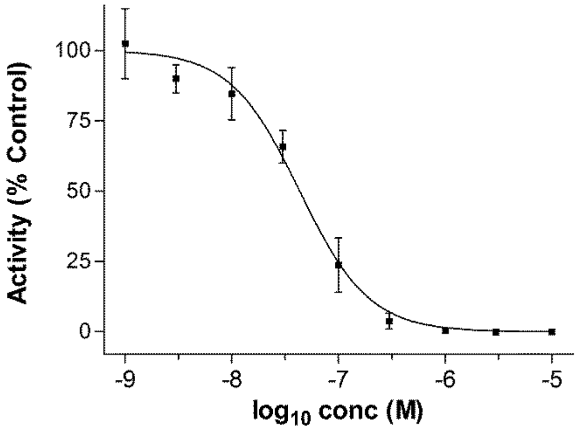

MKP110 v EGFR

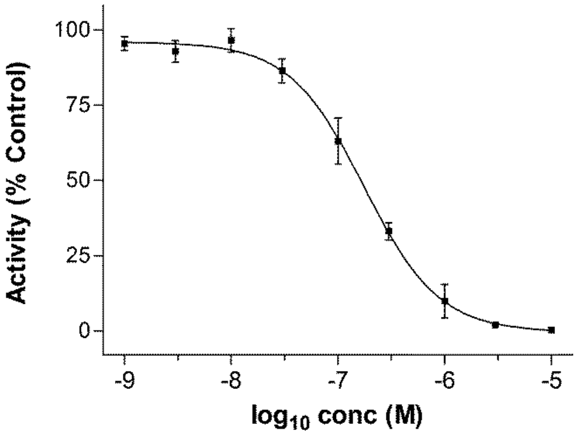

MKP110 v VEGFR-2

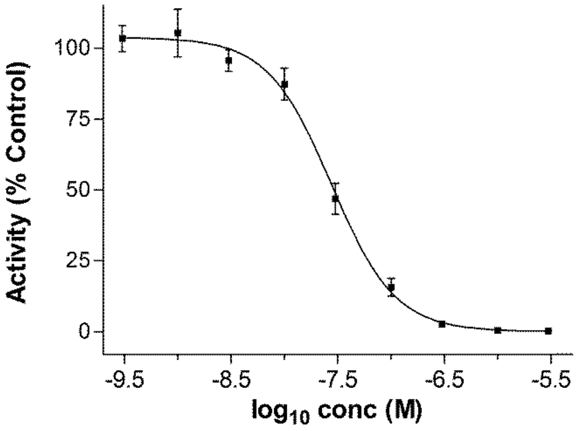

MKP111 v EGFR

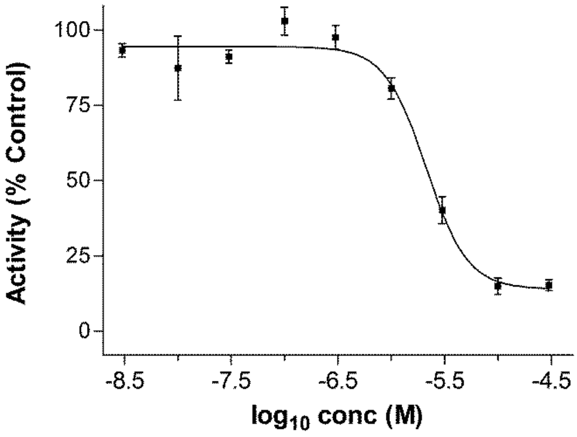

MKP111 v VEGFR-2

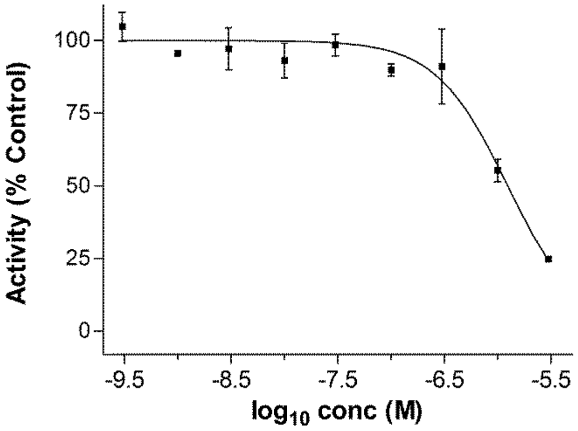

MKP112 v EGFR

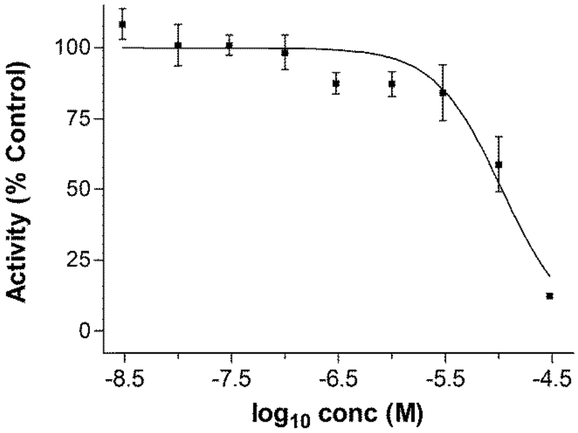

MKP112 v VEGFR-2

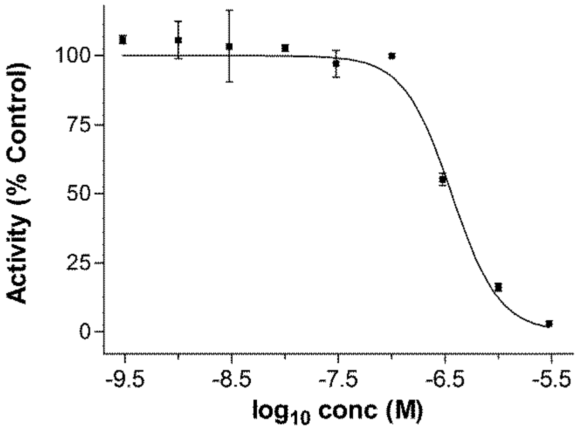

MKP113 v EGFR

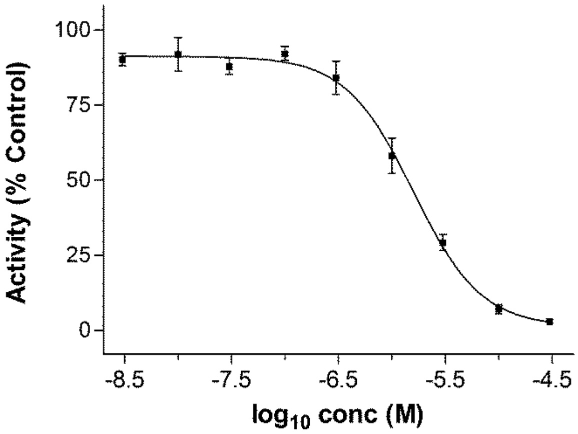

MKP113 v VEGFR-2

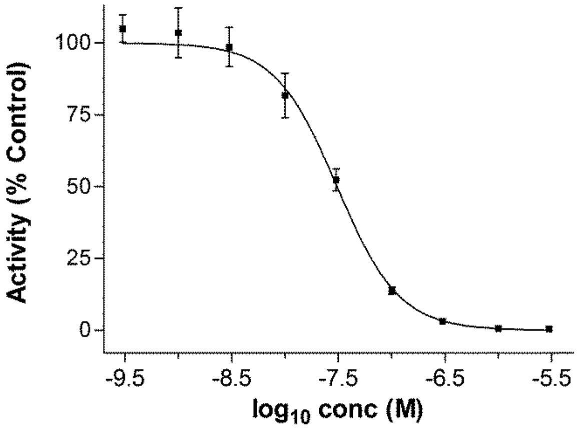

MKP114 v EGFR

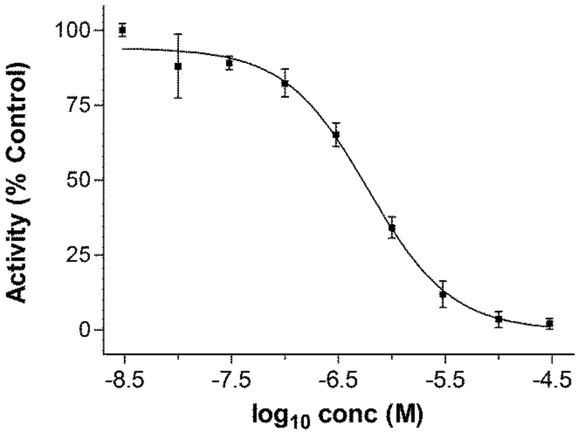

MKP114 v VEGFR-2

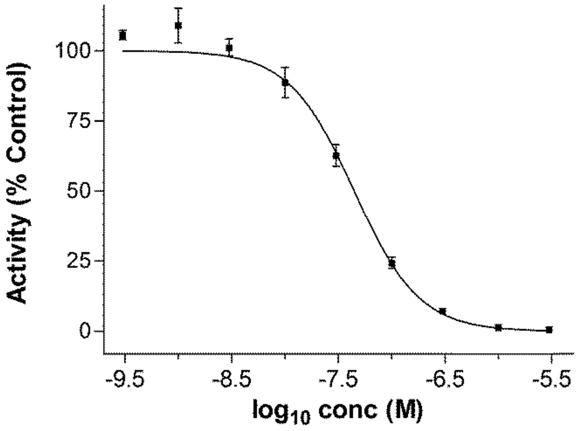

MKP115 v EGFR

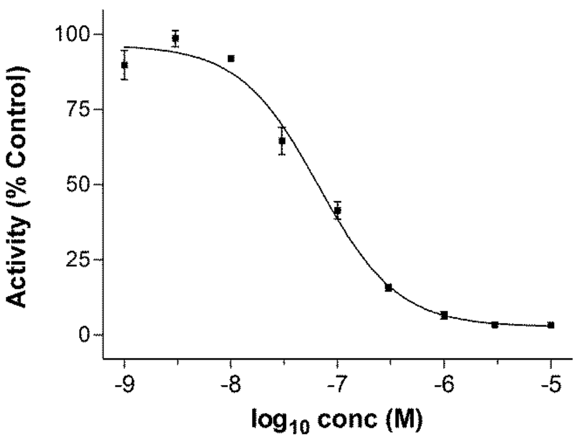

MKP115 v VEGFR-2

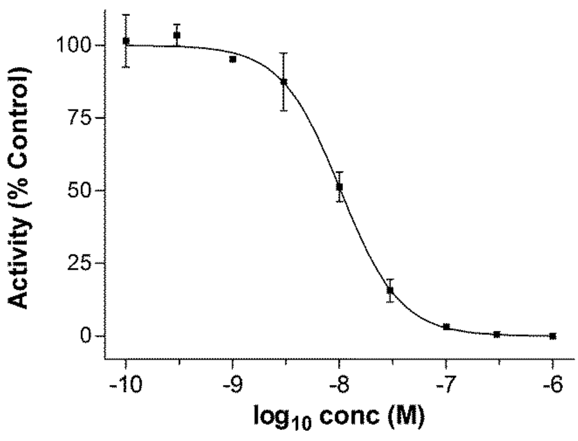

MKP116 v EGFR

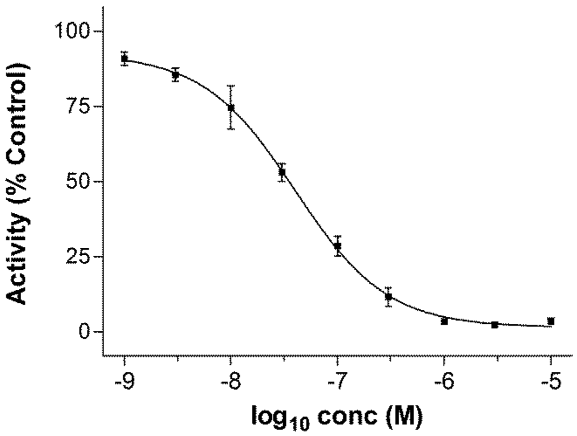

MKP116 v VEGFR-2

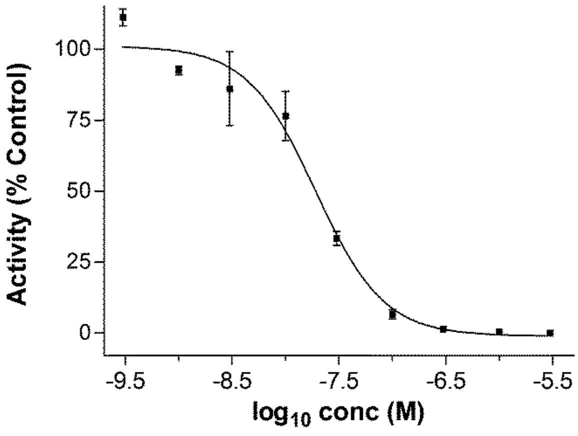

MKP117 v EGFR

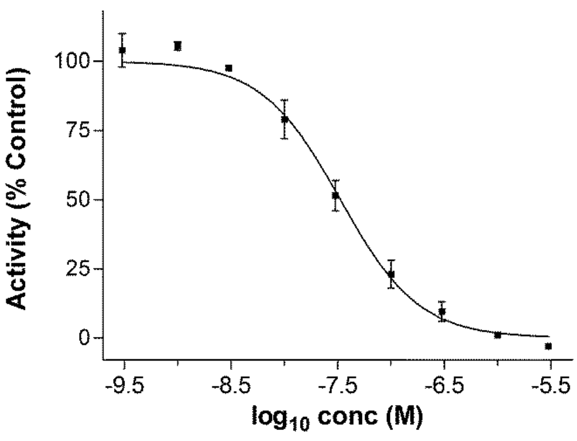

MKP117 v VEGFR-2

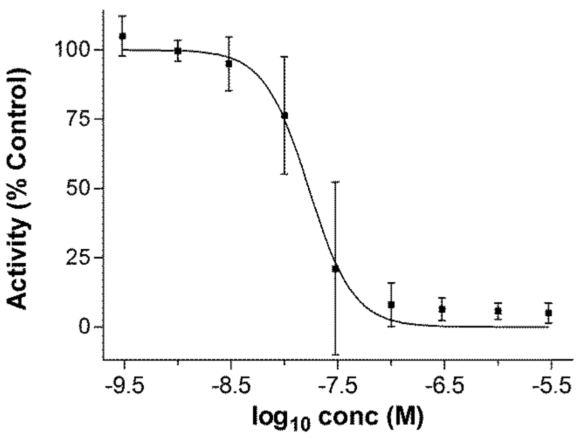

MKP118 v EGFR

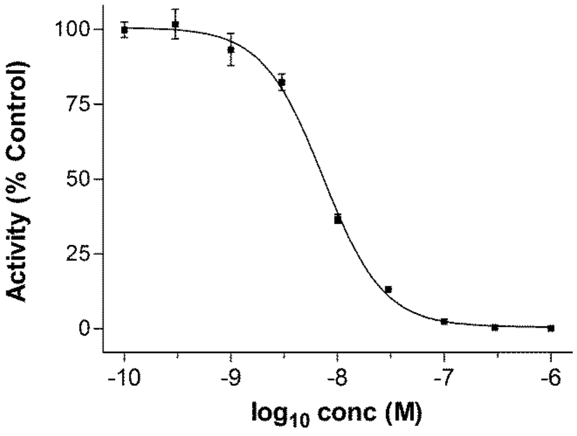

MKP118 v VEGFR-2

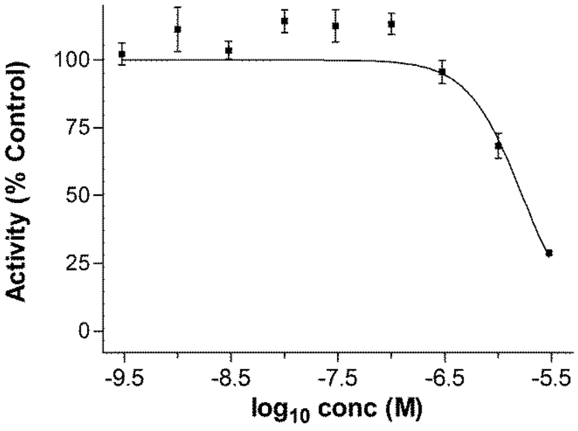

MKP119 v EGFR

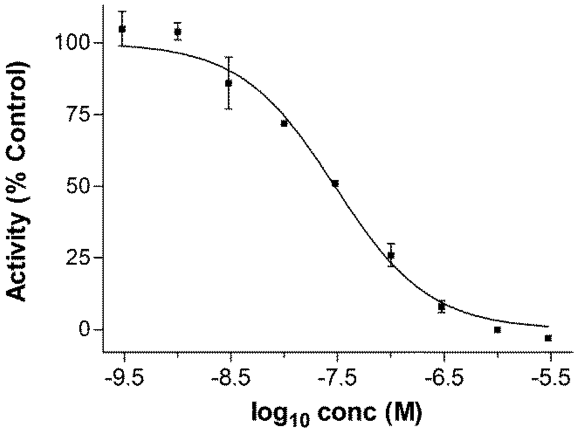

MKP119 v VEGFR-2

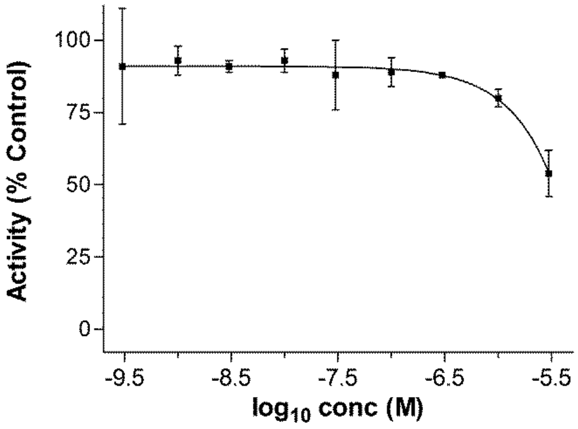

MKP120 v EGFR

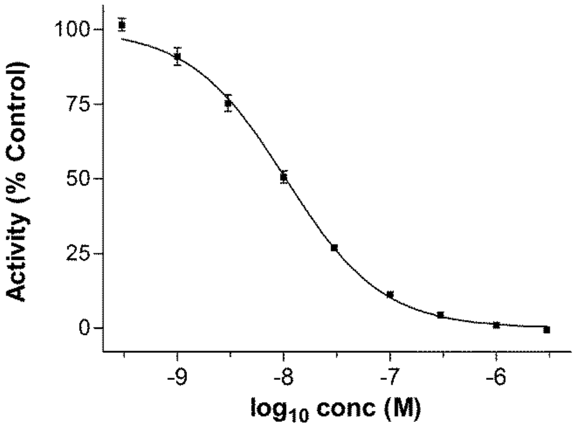

MKP120 v VEGFR-2

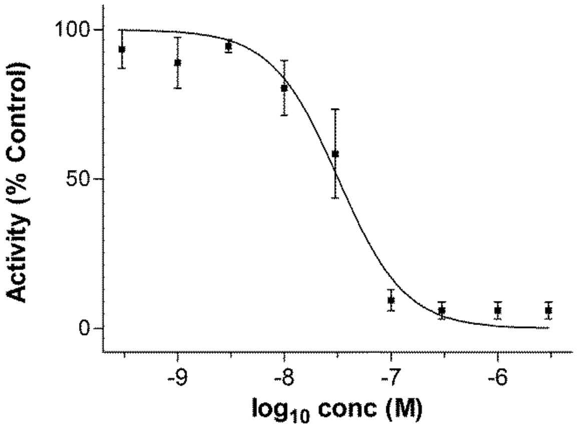

MKP121 v EGFR

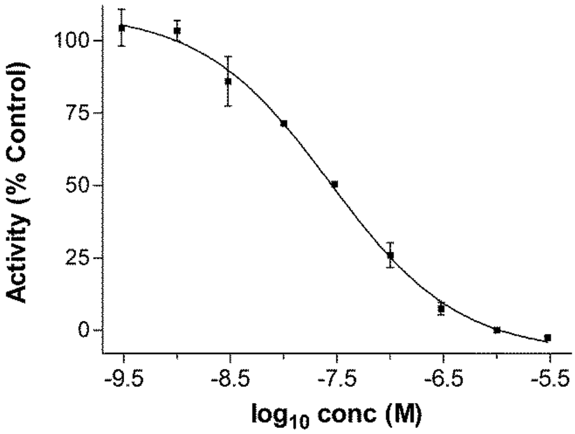

MKP121 v VEGFR-2

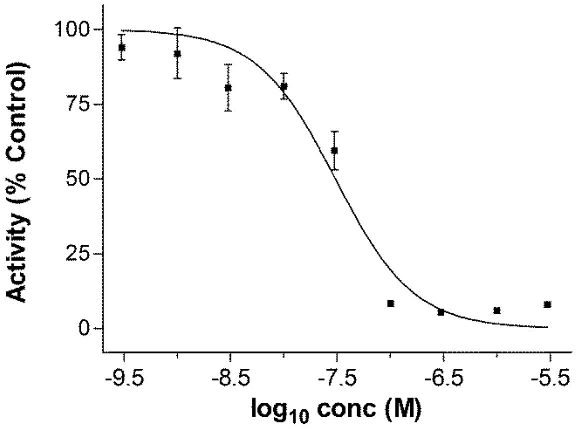

MKP122 v EGFR

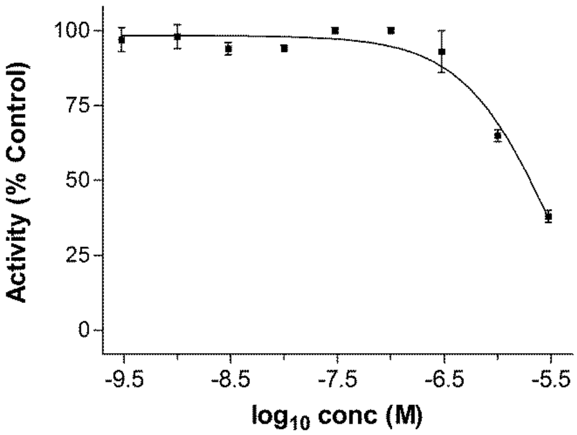

MKP122 v VEGFR-2

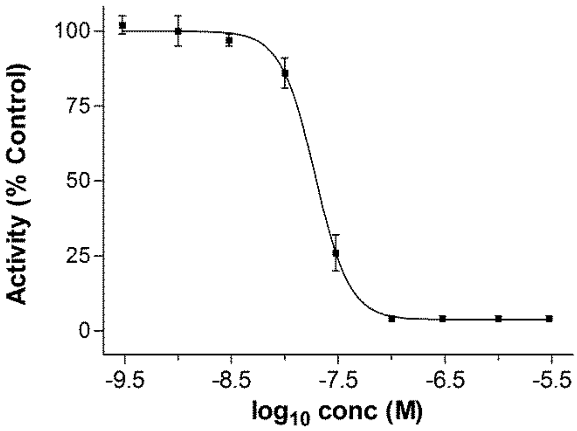

MKP123 v EGFR

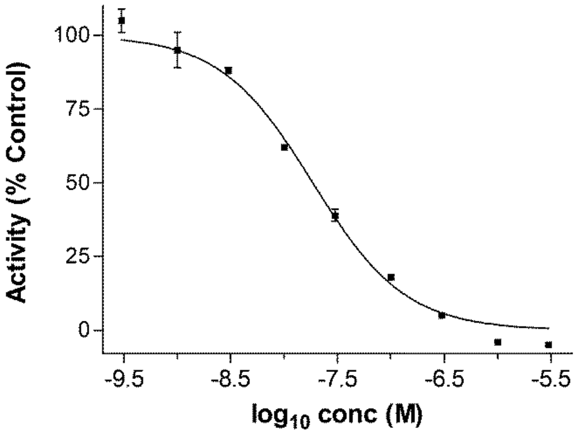

MKP123 v VEGFR-2

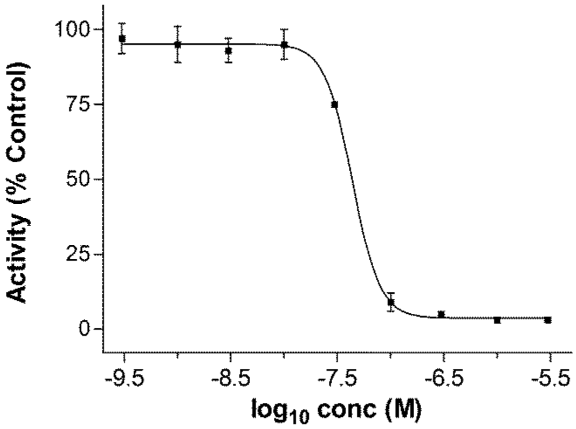

Supplement: S1 Fig — (PDF) [file pone.0138823.s002.pdf]
