# Supplementary figures and images for: Structure-Activity Relationship of Indole-Tethered Pyrimidine Derivatives that Concurrently Inhibit Epidermal Growth Factor Receptor and Other Angiokinases
Source: PLoS One. 2015 Sep 24;10(9):e0138823. doi: 10.1371/journal.pone.0138823 (PMC4581874; doi:10.1371/journal.pone.0138823)

**S2 Fig.** IC<sub>50</sub> graphs of MKP101-105 against HCC827.

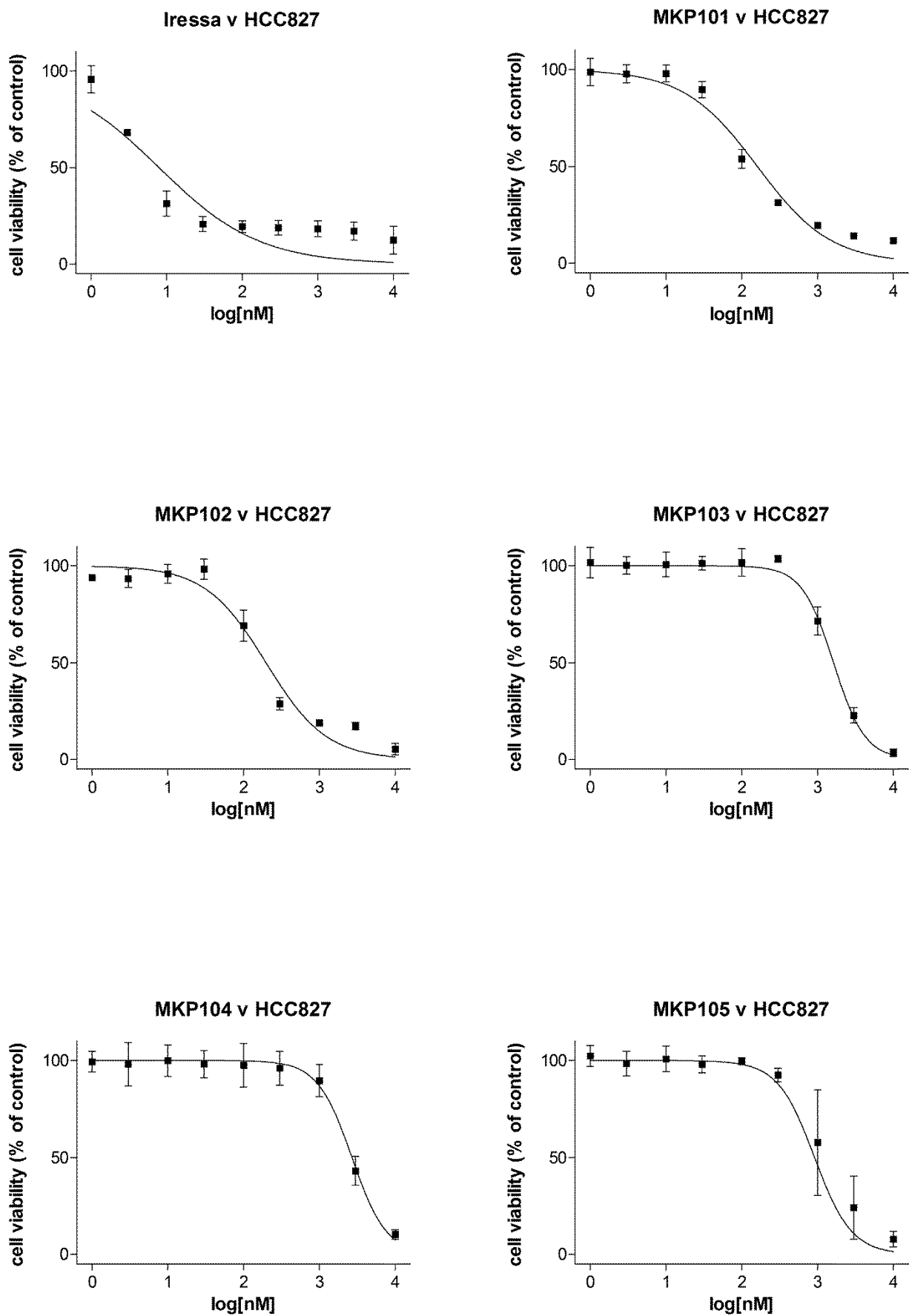

Supplement: S2 Fig — (PDF) [file pone.0138823.s003.pdf]
