# Supplementary material for: Structure-Activity Relationship of Indole-Tethered Pyrimidine Derivatives that Concurrently Inhibit Epidermal Growth Factor Receptor and Other Angiokinases
Source: PLoS One. 2015 Sep 24;10(9):e0138823. doi: 10.1371/journal.pone.0138823 (PMC4581874; doi:10.1371/journal.pone.0138823)
Supplement: S3 Fig — (PDF) [file pone.0138823.s004.pdf]

S3 Fig. NMR spectra for MKP101-123

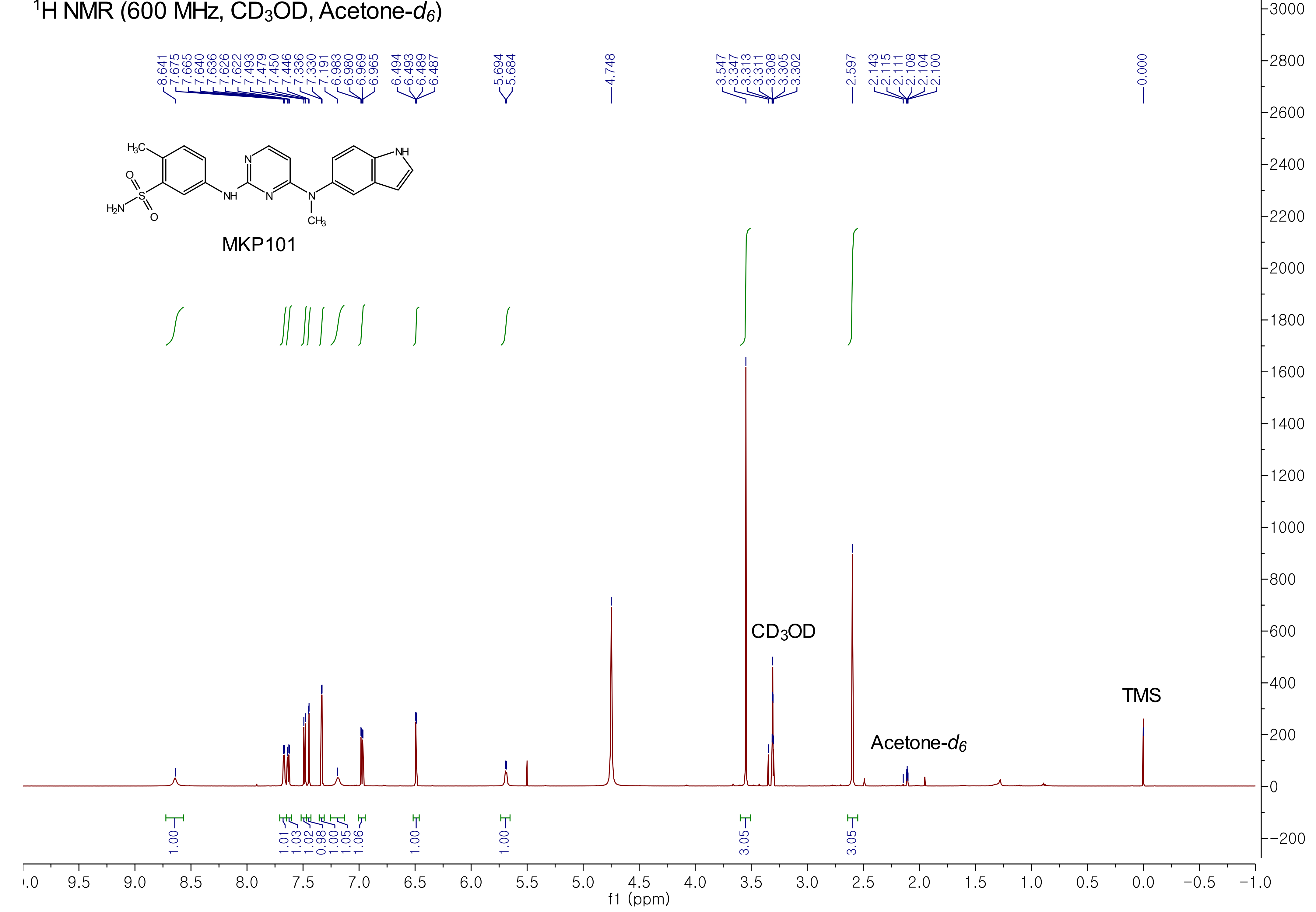

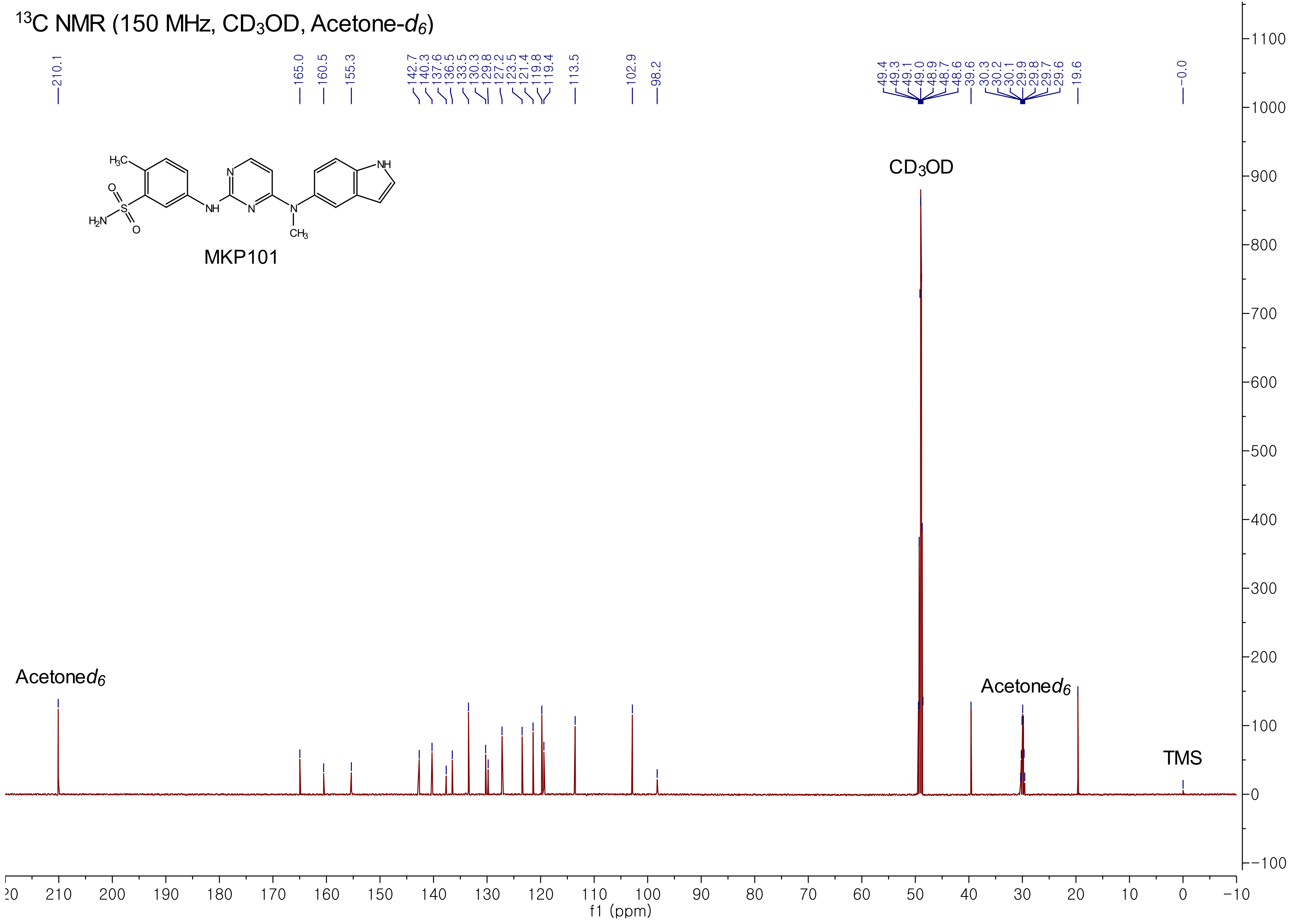
13C NMR (150 MHz, CD3OD, Acetone-d6)
210.1
165.0
160.5
155.3
142.7
140.3
137.6
136.5
133.5
130.3
129.8
127.2
123.5
121.4
119.8
119.4
113.5
102.9
98.2
49.4
49.3
49.1
49.0
48.9
48.7
48.6
39.6
30.3
30.2
30.1
29.9
29.8
29.7
29.6
19.6
-0.0
MKP101
CD3OD
Acetoned6
Acetoned6
TMS
f1 (ppm)

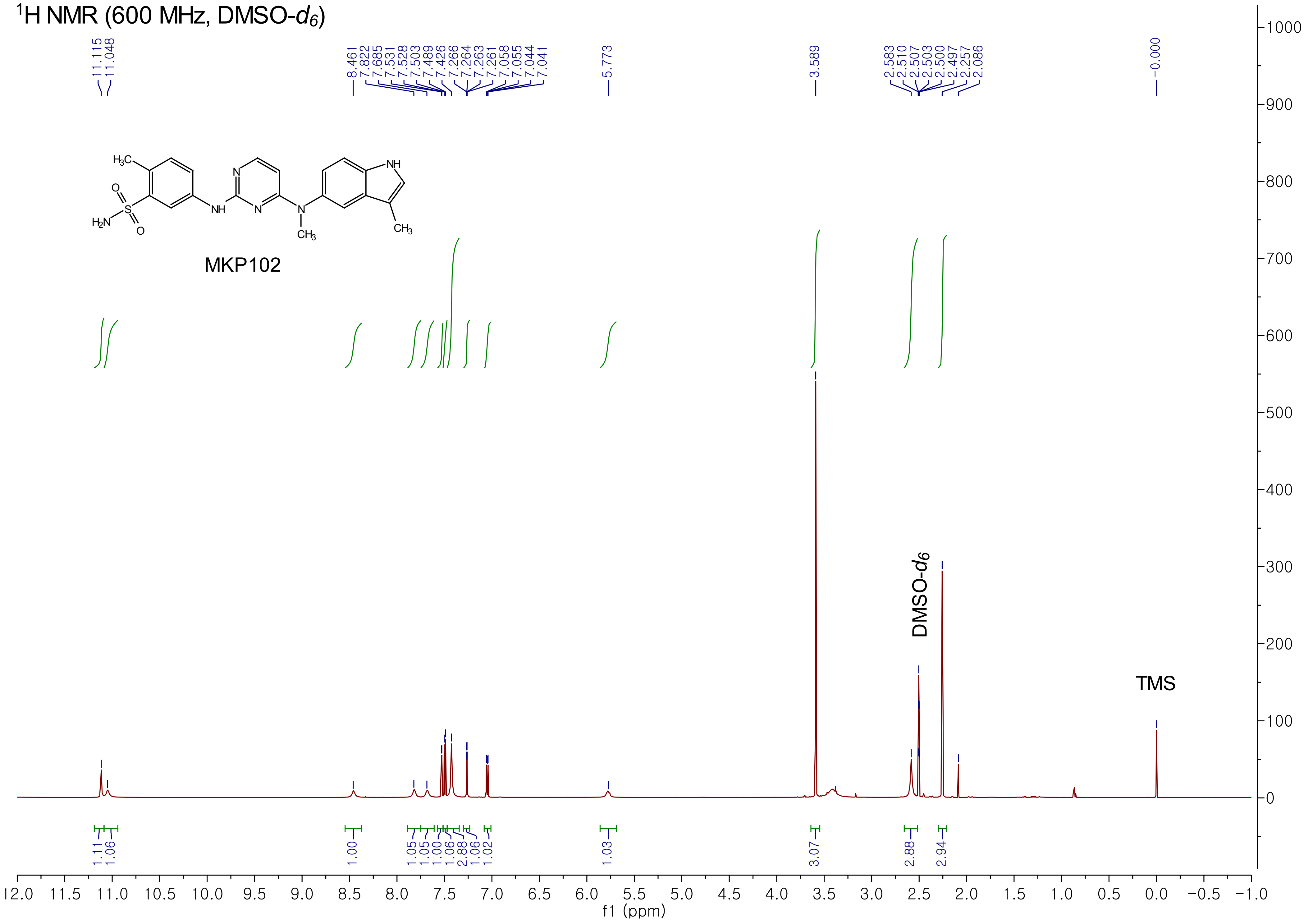

^1H NMR (600 MHz, DMSO-d_6)
MKP102
DMSO-d_6
TMS
f1 (ppm)
11.115
11.048
8.461
7.822
7.685
7.531
7.528
7.503
7.489
7.426
7.266
7.264
7.263
7.261
7.058
7.055
7.044
7.041
5.773
3.589
2.583
2.510
2.507
2.503
2.500
2.497
2.257
2.086
-0.000

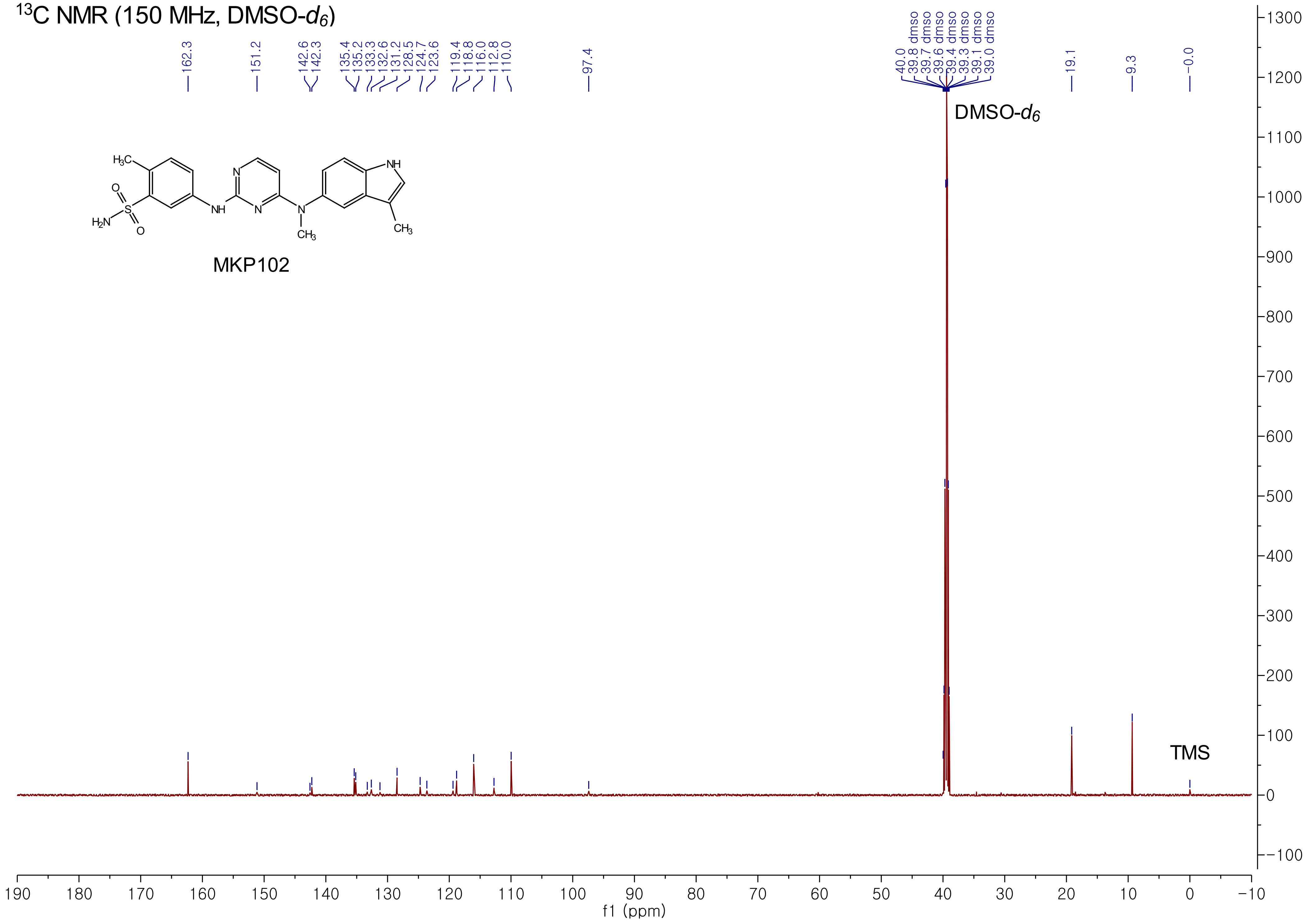

^{13}C NMR (150 MHz, DMSO-d_6)
162.3
151.2
142.6
142.3
135.4
135.2
133.3
132.6
131.2
128.5
124.7
123.6
119.4
118.8
116.0
112.8
110.0
97.4
40.0 dmso
39.8 dmso
39.7 dmso
39.6 dmso
39.4 dmso
39.3 dmso
39.1 dmso
39.0 dmso
19.1
9.3
-0.0
DMSO-d_6
TMS
MKP102
f1 (ppm)

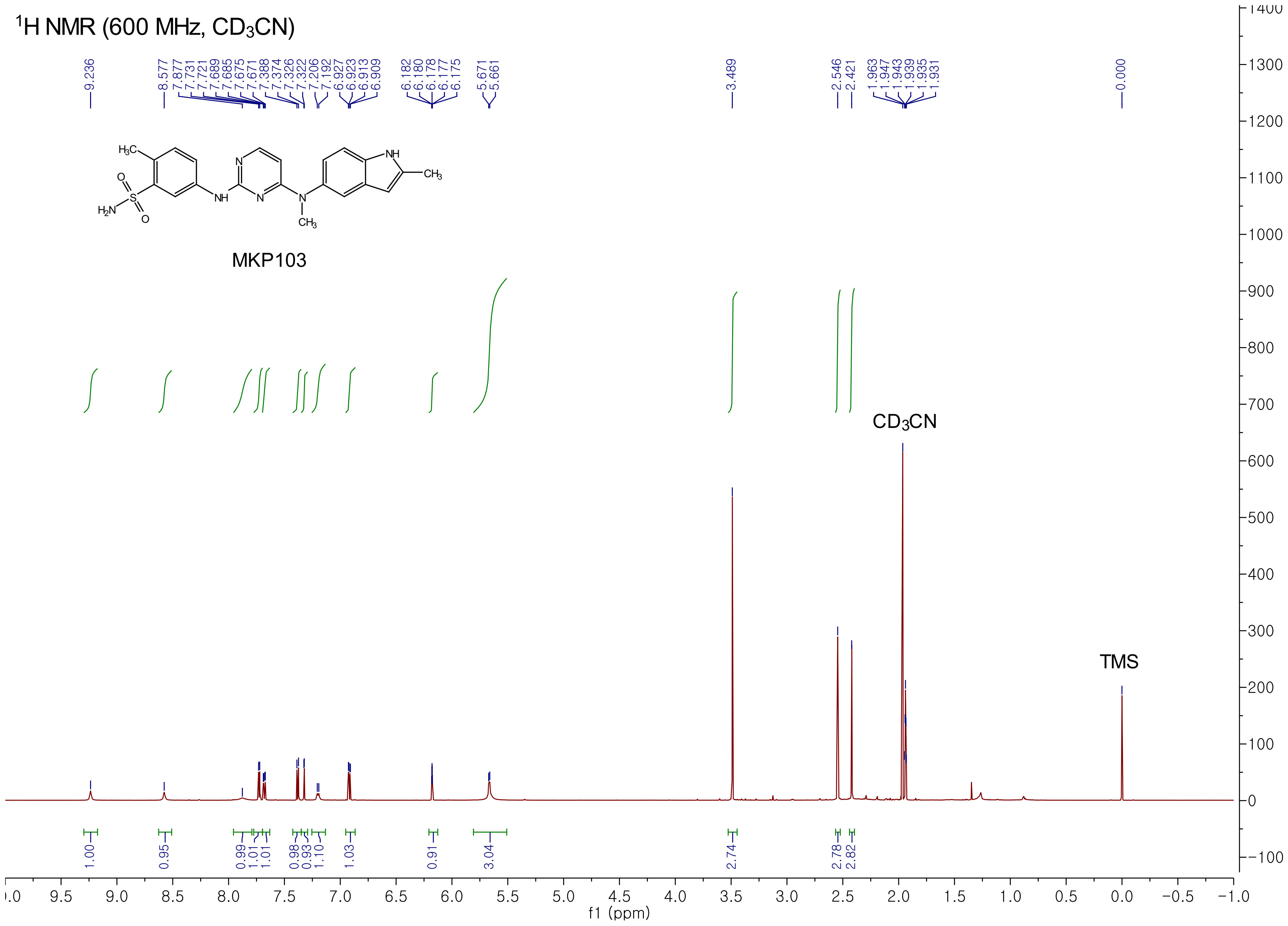

¹H NMR (600 MHz, CD₃CN)
9.236
8.577 7.877 7.731 7.721 7.689 7.685 7.675 7.671 7.388 7.374 7.326 7.322 7.206 7.192 6.927 6.923 6.913 6.909
6.182 6.180 6.178 6.177 6.175
5.671 5.661
3.489
2.546 2.421
1.963 1.947 1.943 1.939 1.935 1.931
0.000
MKP103
CD₃CN
TMS
1.00 0.95 0.99 1.01 1.01 0.98 0.93 1.10 1.03 0.91 3.04 2.74 2.78 2.82
f1 (ppm)

^{13}C NMR (150 MHz, CD_3CN)

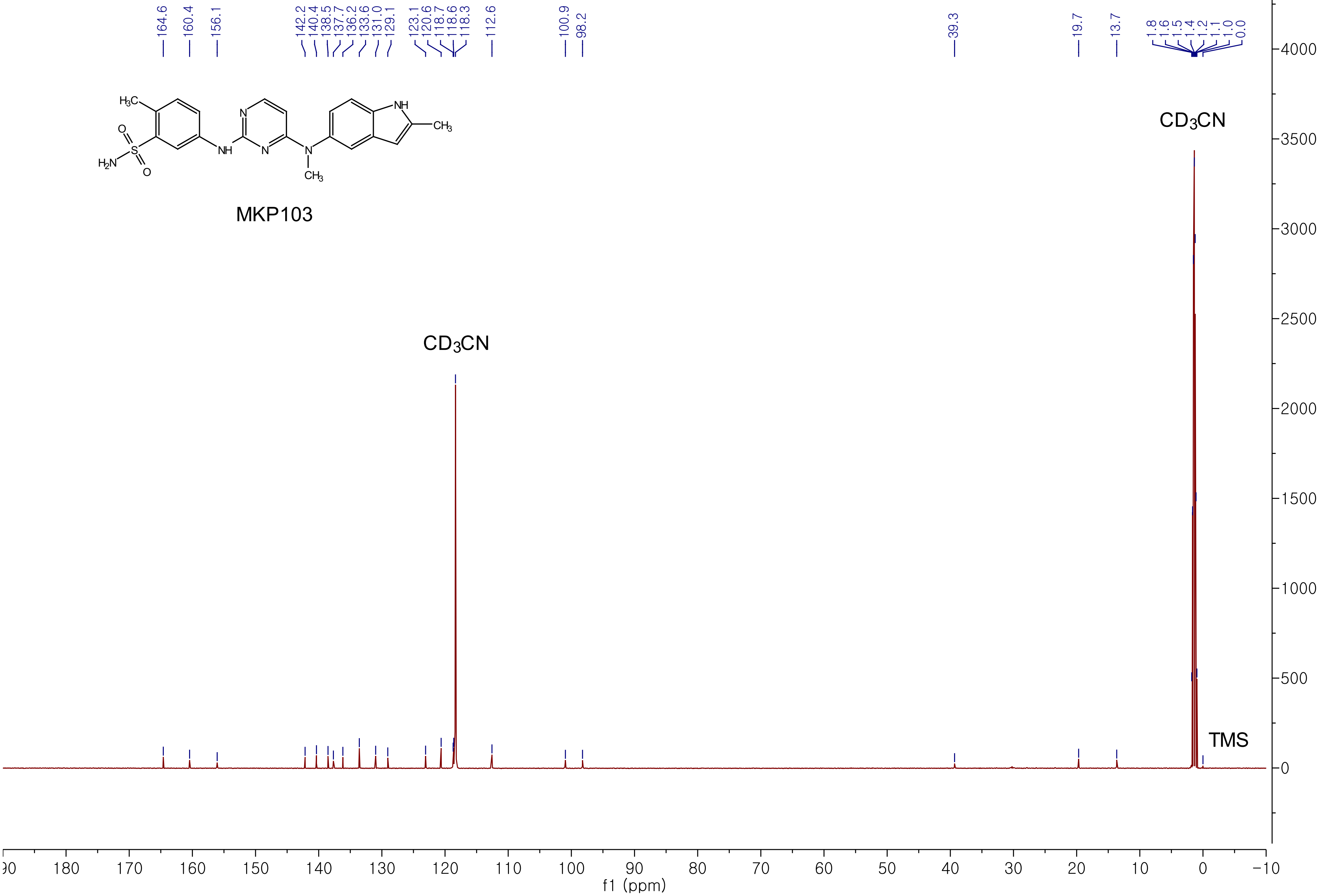

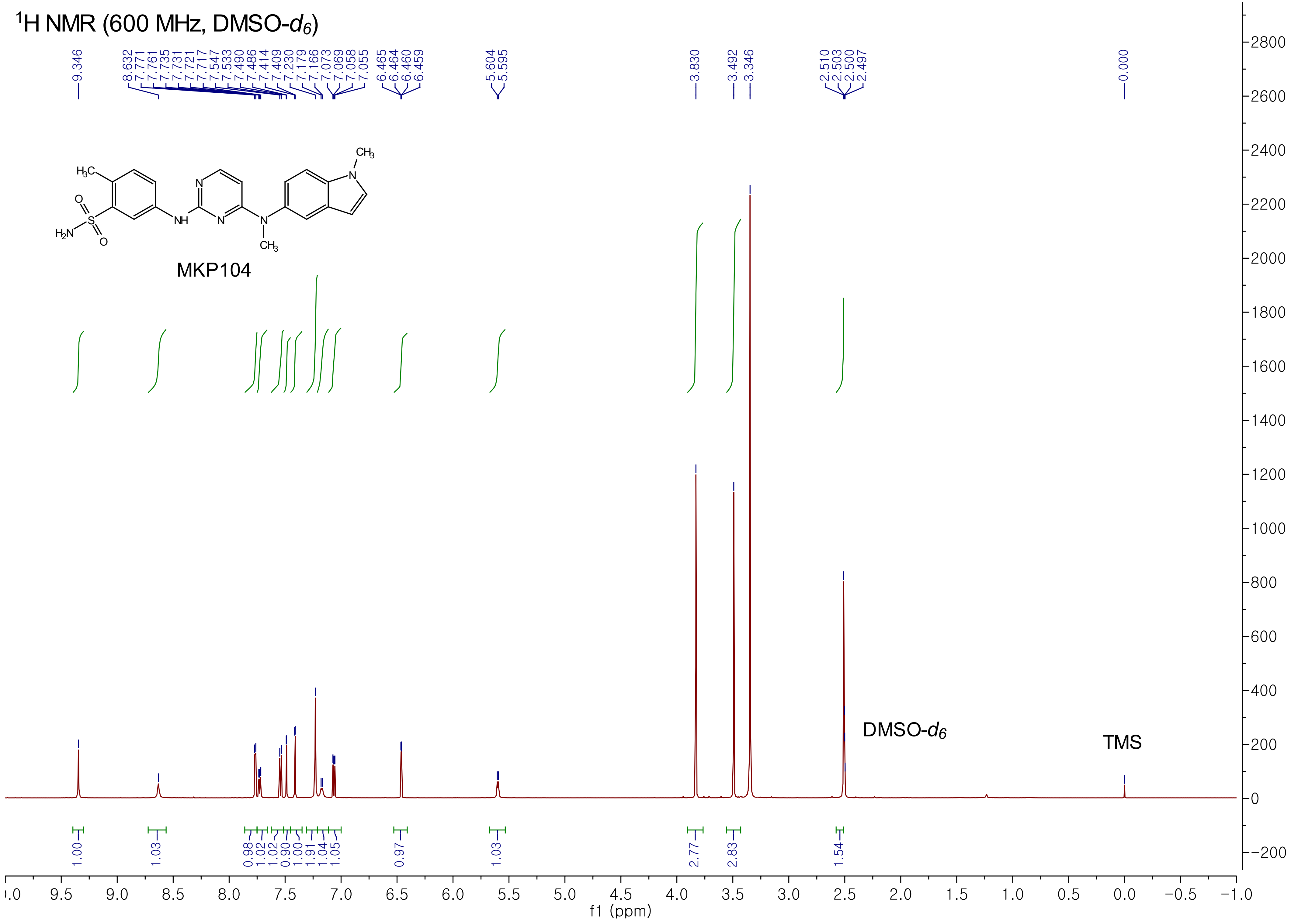

^{1}H NMR (600 MHz, DMSO-d_6)
MKP104
DMSO-d_6
TMS
f1 (ppm)

^{13}C NMR (150 MHz, DMSO-d_6)

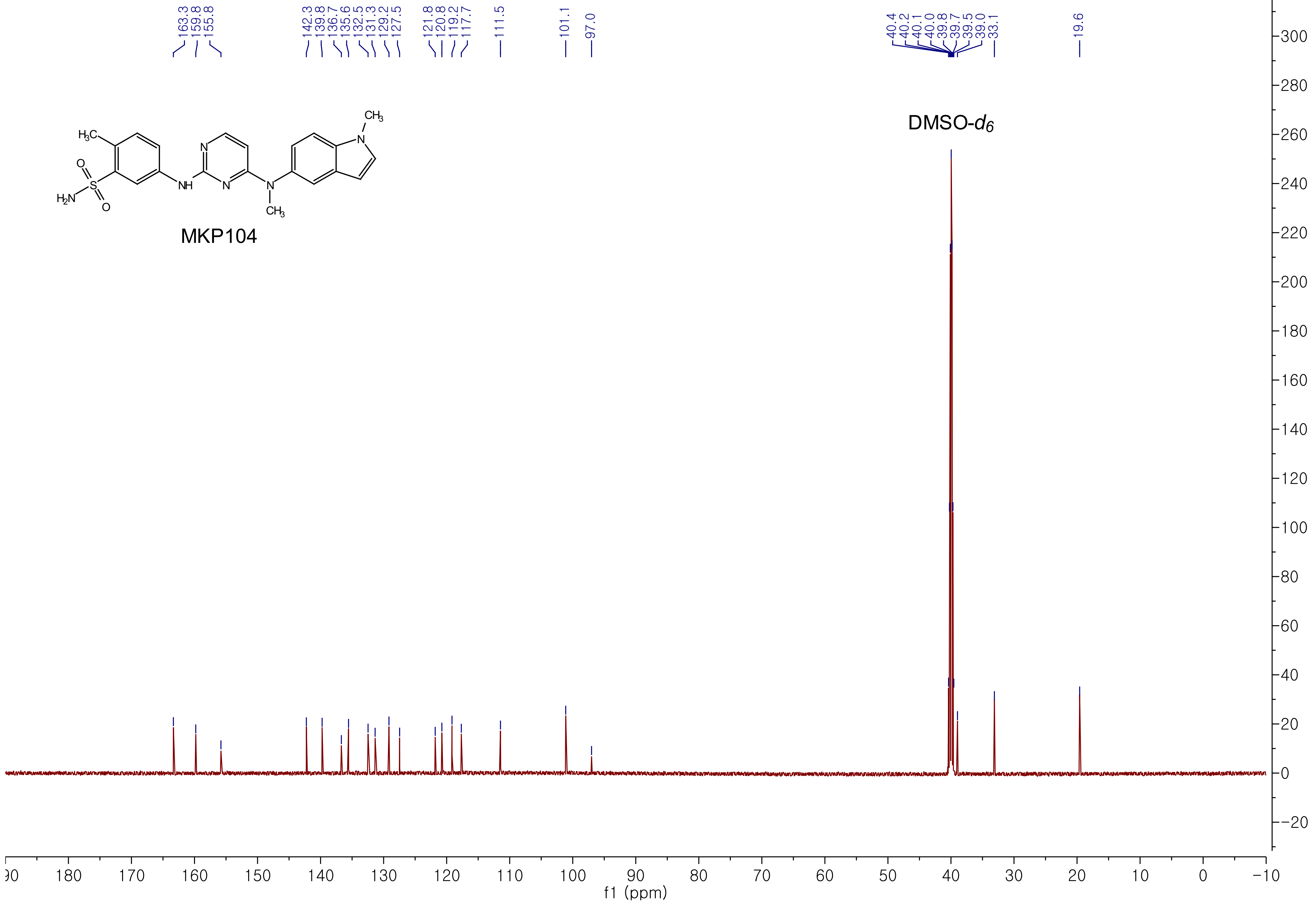

^1H NMR (600 MHz, CD_3OD)

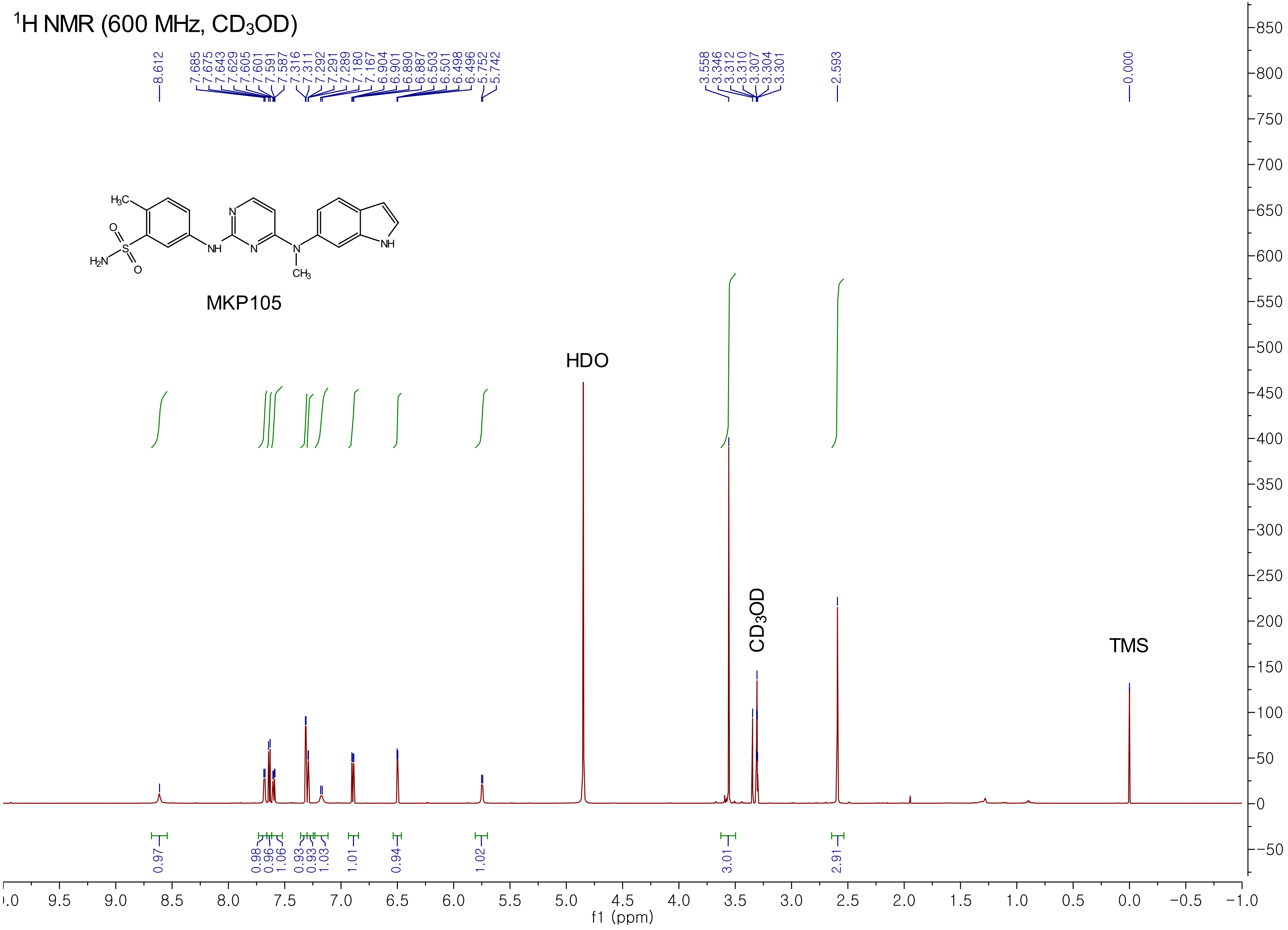

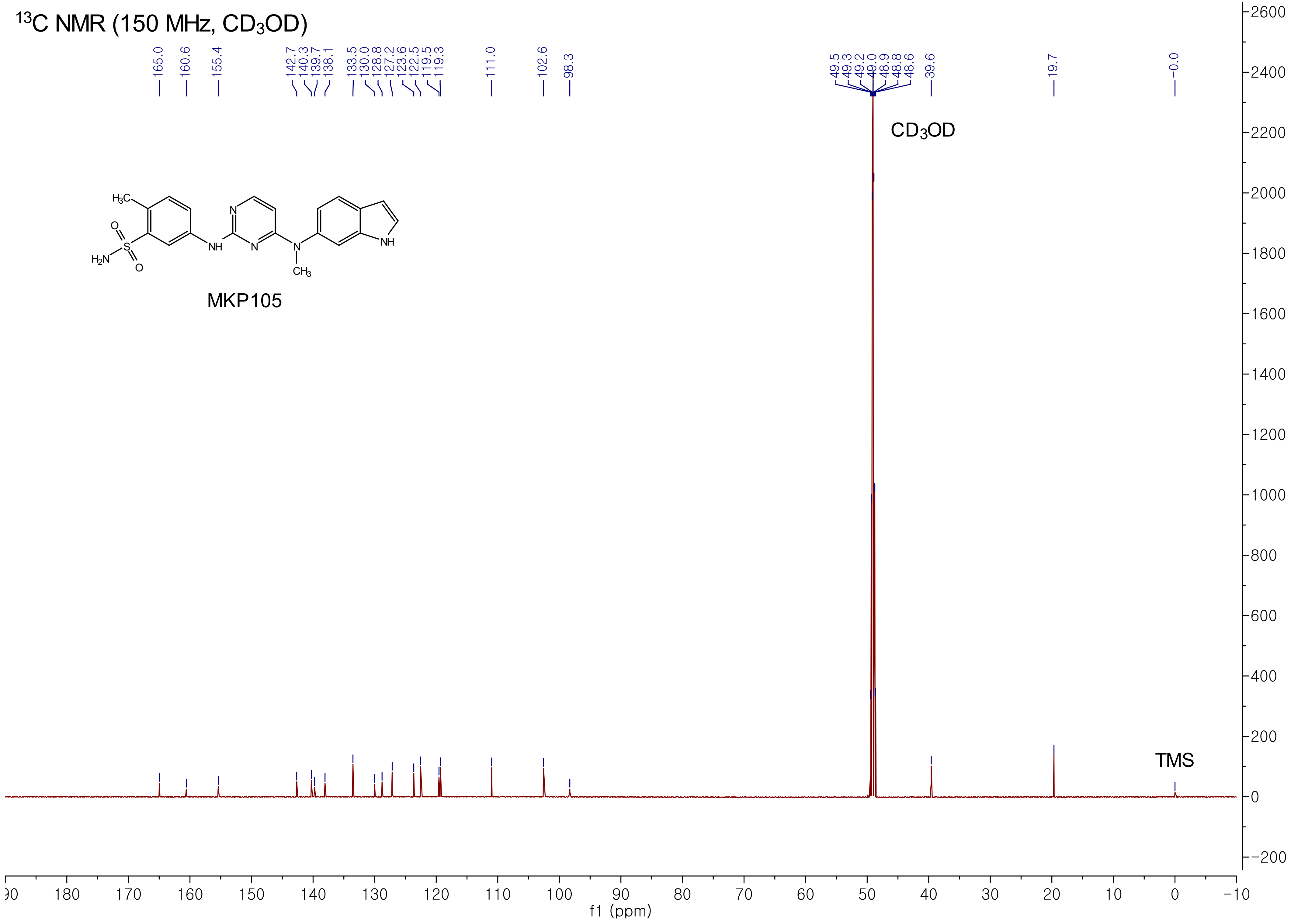

13C NMR (150 MHz, CD3OD)
165.0
160.6
155.4
142.7
140.3
139.7
138.1
133.5
130.0
128.8
127.2
123.6
122.5
119.5
119.3
111.0
102.6
98.3
49.5
49.3
49.2
49.0
48.9
48.8
48.6
39.6
19.7
-0.0
CD3OD
H3C
O
S
H2N
O
NH
N
N
N
CH3
NH
MKP105
TMS
f1 (ppm)

^1H NMR (600 MHz, $CDCl_3$)

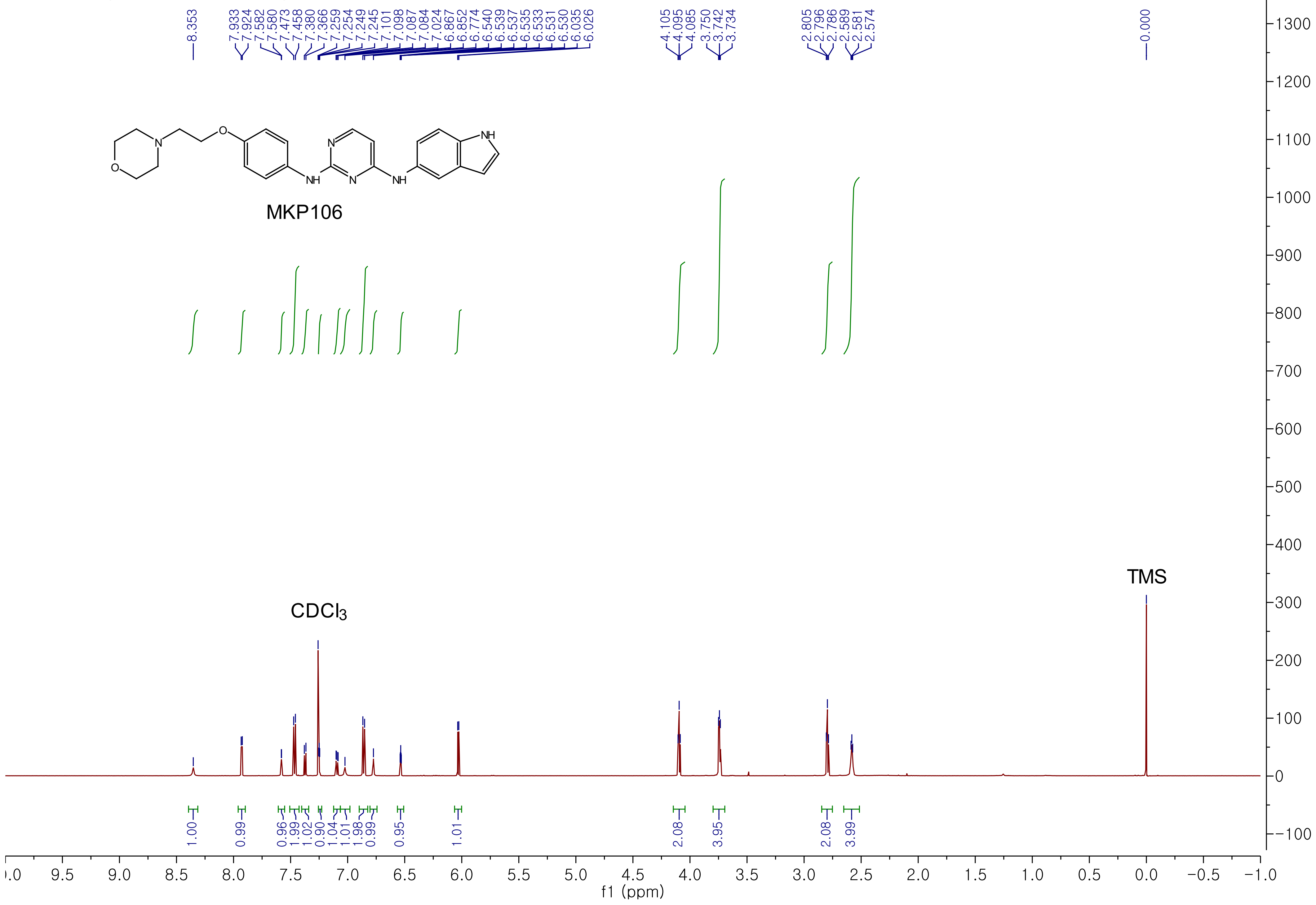

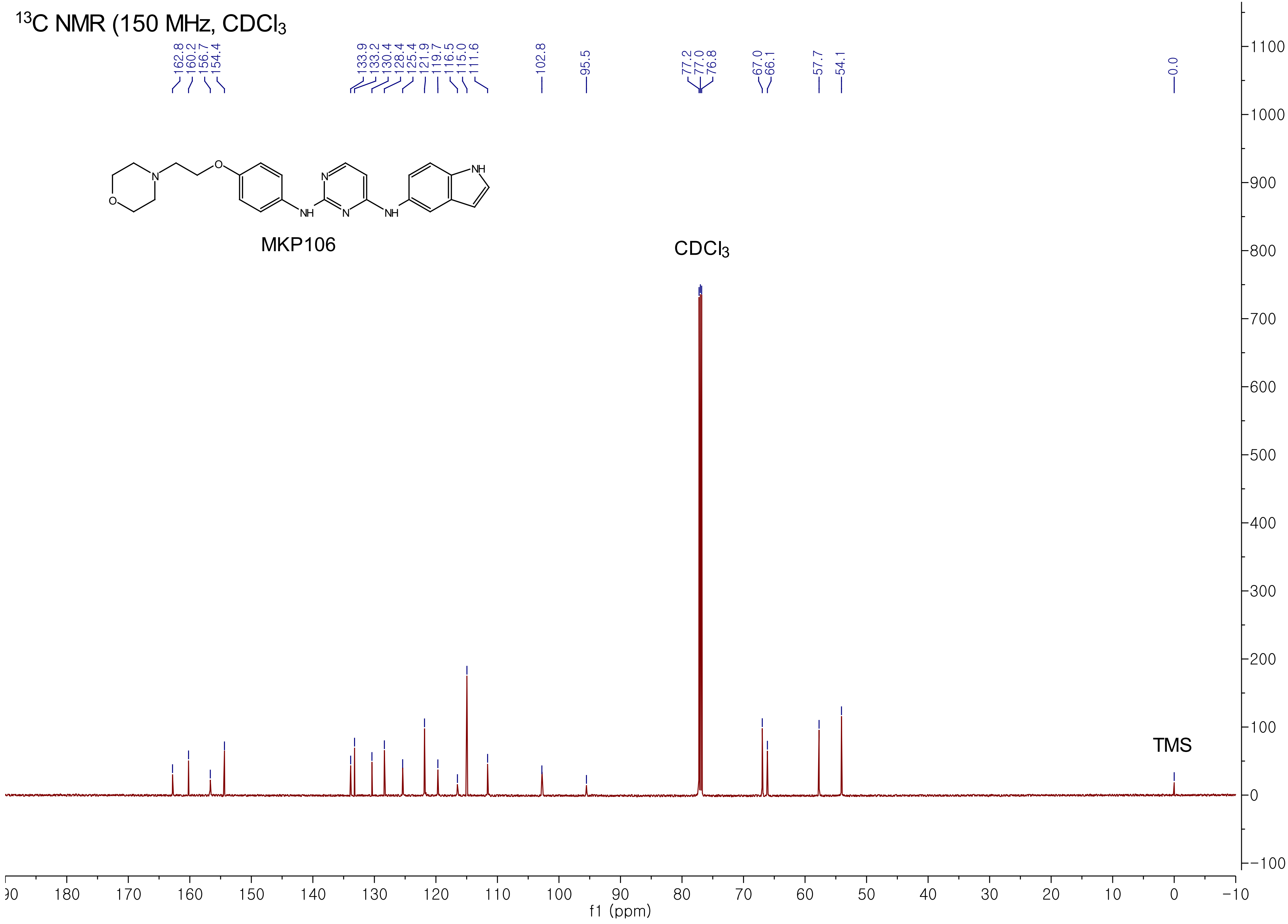

13C NMR (150 MHz, CDCl3)
162.8
160.2
156.7
154.4
133.9
133.2
130.4
128.4
125.4
121.9
119.7
116.5
115.0
111.6
102.8
95.5
77.2
77.0
76.8
67.0
66.1
57.7
54.1
0.0
MKP106
CDCl3
TMS
f1 (ppm)

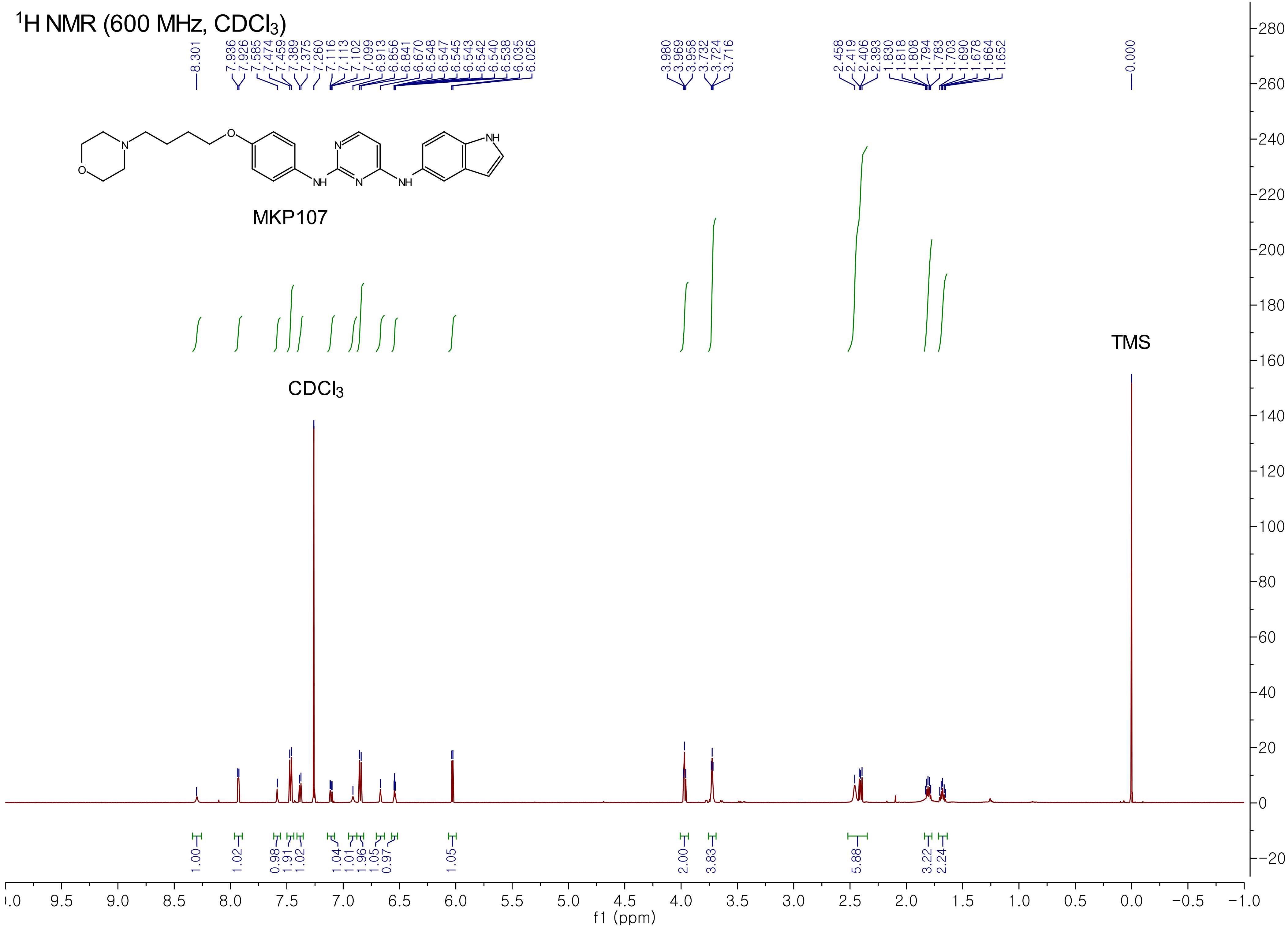

1H NMR (600 MHz, CDCl3)
8.301
7.936
7.926
7.585
7.474
7.459
7.389
7.375
7.260
7.116
7.113
7.102
7.099
6.913
6.856
6.841
6.670
6.548
6.547
6.545
6.543
6.542
6.540
6.538
6.035
6.026
3.980
3.969
3.958
3.732
3.724
3.716
2.458
2.419
2.406
2.393
1.830
1.818
1.808
1.794
1.783
1.703
1.690
1.678
1.664
1.652
0.000
MKP107
CDCl3
TMS
1.00
1.02
0.98
1.91
1.02
1.04
1.01
1.96
1.05
0.97
1.05
2.00
3.83
5.88
3.22
2.24
f1 (ppm)

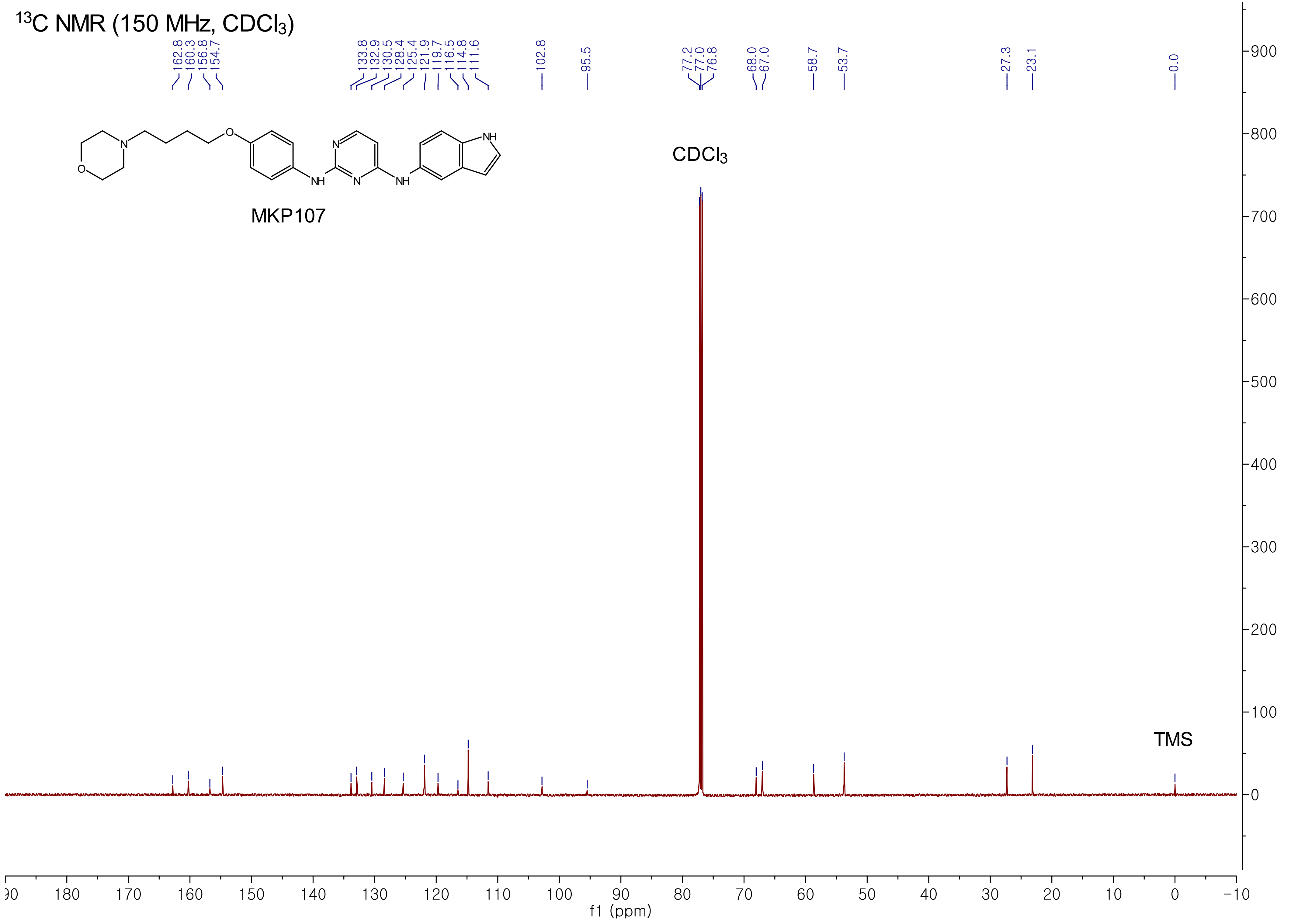

^{13}C NMR (150 MHz, CDCl$_3$)
162.8
160.3
156.8
154.7
133.8
132.9
130.5
128.4
125.4
121.9
119.7
116.5
114.8
111.6
102.8
95.5
77.2
77.0
76.8
68.0
67.0
58.7
53.7
27.3
23.1
0.0
MKP107
CDCl$_3$
TMS
f1 (ppm)

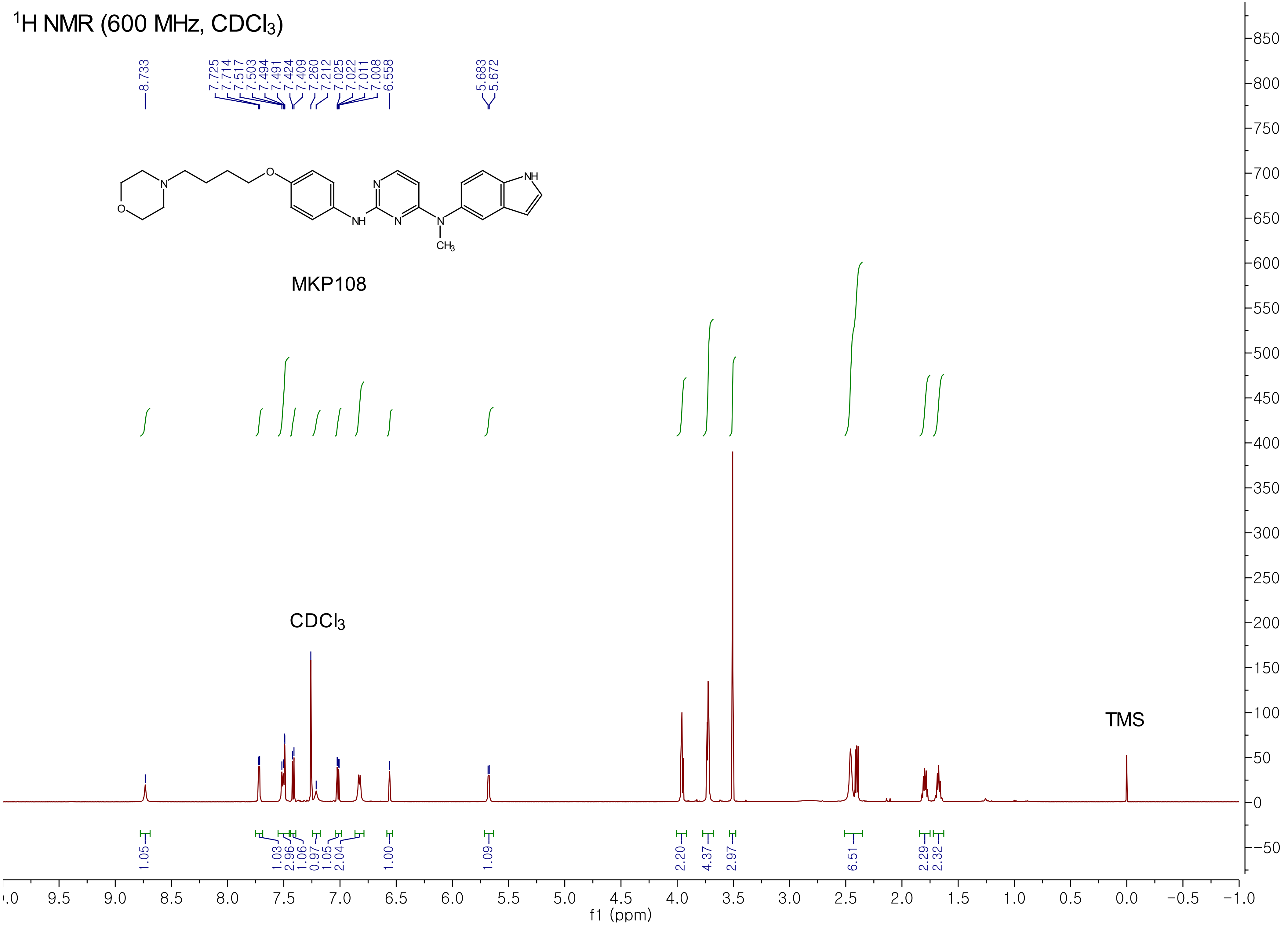

^1H NMR (600 MHz, $CDCl_3$)
8.733
7.725
7.714
7.517
7.503
7.494
7.491
7.424
7.409
7.260
7.212
7.025
7.022
7.011
7.008
6.558
5.683
5.672
NH
N
O
O
NH
N
N
N
CH_3
MKP108
$CDCl_3$
TMS
1.05
1.03
2.96
1.06
0.97
1.05
2.04
1.00
1.09
2.20
4.37
2.97
6.51
2.29
2.32
f1 (ppm)

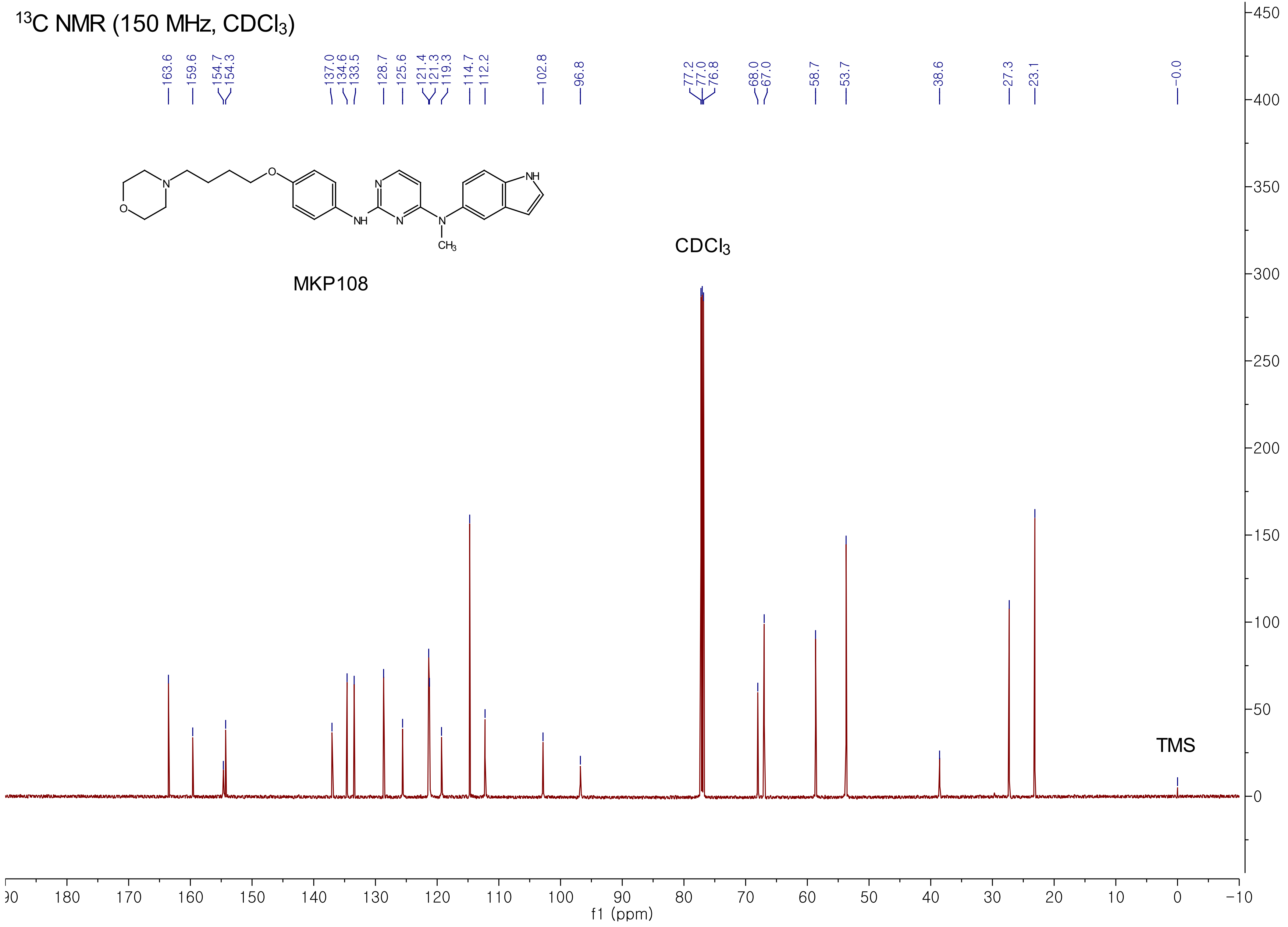

^{13}C NMR (150 MHz, $CDCl_3$)
163.6
159.6
154.7
154.3
137.0
134.6
133.5
128.7
125.6
121.4
121.3
119.3
114.7
112.2
102.8
96.8
77.2
77.0
76.8
68.0
67.0
58.7
53.7
38.6
27.3
23.1
-0.0
MKP108
$CDCl_3$
TMS
f1 (ppm)

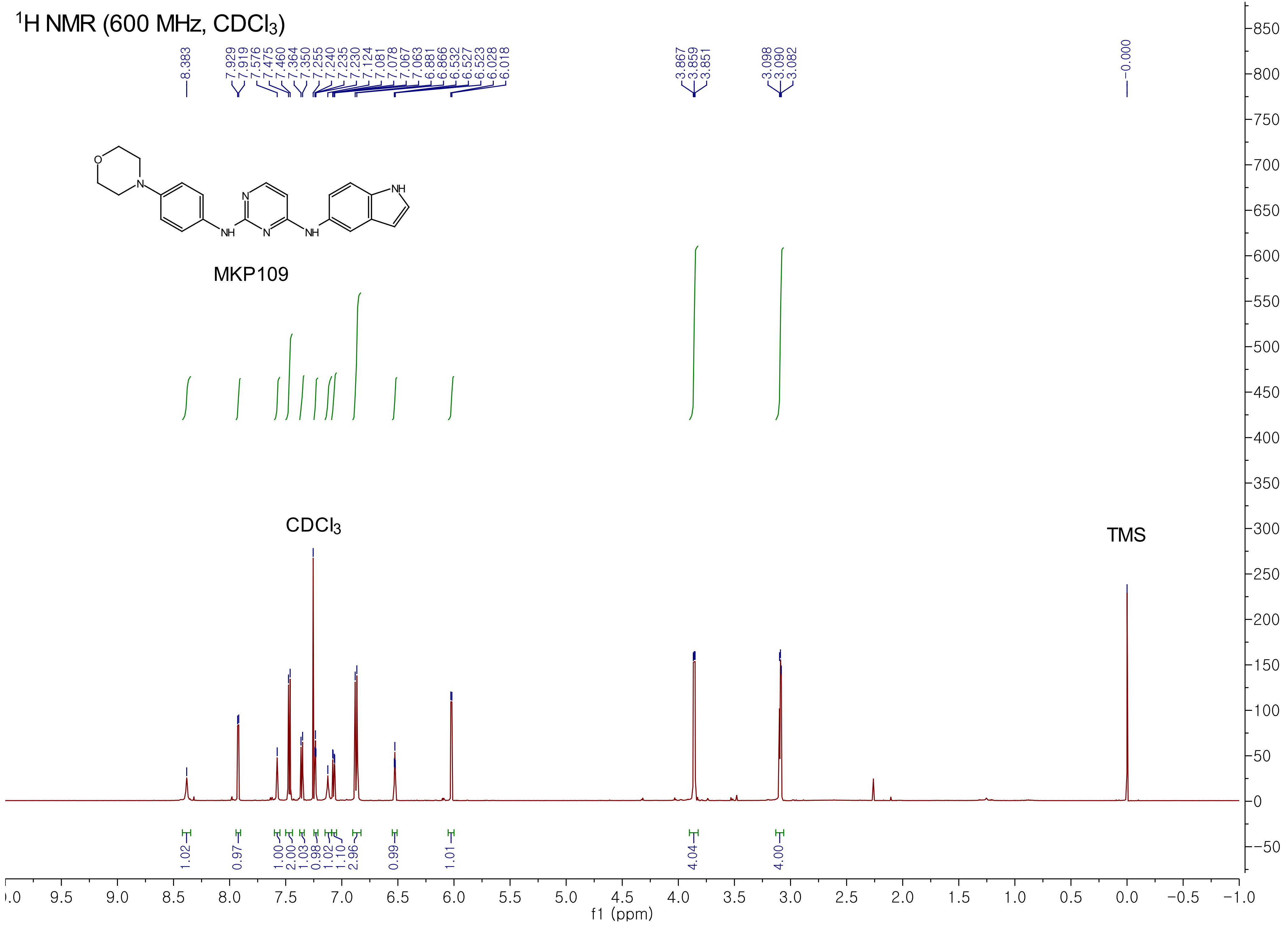

1H NMR (600 MHz, CDCl3)
8.383
7.929
7.919
7.576
7.475
7.460
7.364
7.350
7.255
7.240
7.235
7.230
7.124
7.081
7.078
7.067
7.063
6.881
6.866
6.532
6.527
6.523
6.028
6.018
3.867
3.859
3.851
3.098
3.090
3.082
-0.000
O
N
N
N
NH
NH
NH
MKP109
CDCl3
TMS
1.02
0.97
1.00
2.00
1.03
0.98
1.02
1.10
2.96
0.99
1.01
4.04
4.00
f1 (ppm)

^{13}C NMR (150 MHz, $CDCl_3$)

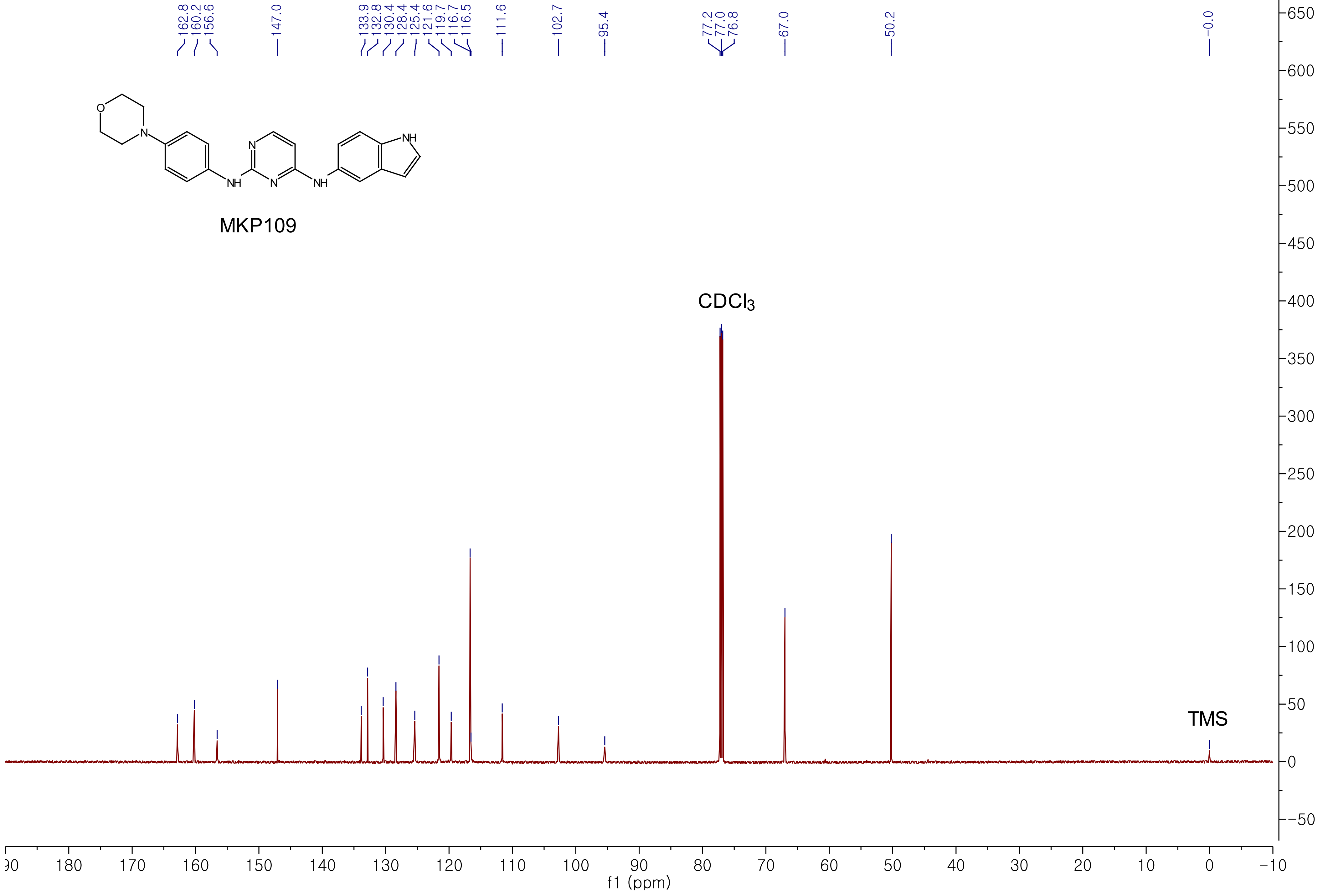

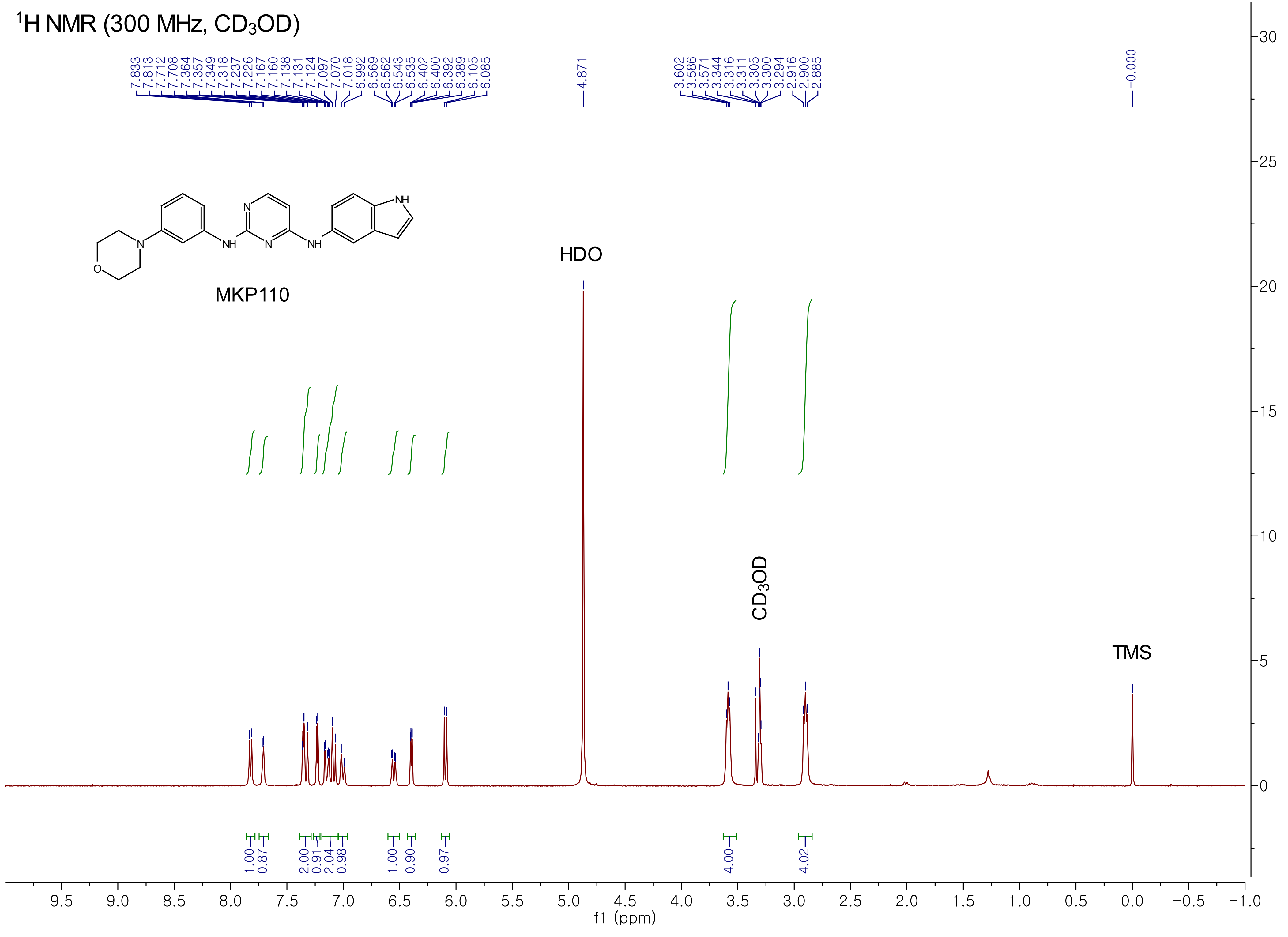

^{1}H NMR (300 MHz, CD_3OD)
MKP110
HDO
CD_3OD
TMS
f1 (ppm)

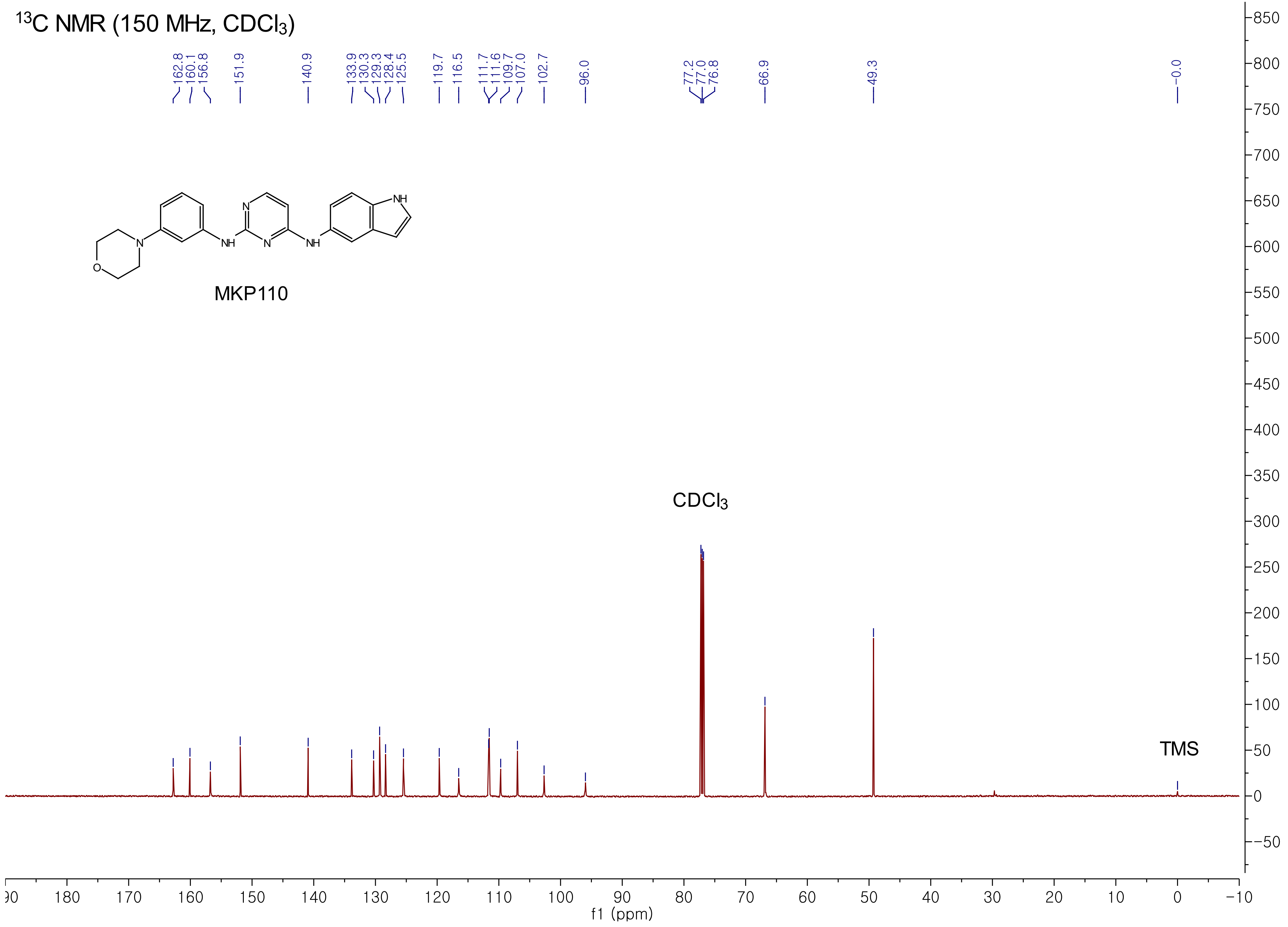

^{13}C NMR (150 MHz, CDCl$_3$)
162.8
160.1
156.8
151.9
140.9
133.9
130.3
129.3
128.4
125.5
119.7
116.5
111.7
111.6
109.7
107.0
102.7
96.0
77.2
77.0
76.8
66.9
49.3
-0.0
NH
N
N
NH
NH
O
N
MKP110
CDCl$_3$
TMS
f1 (ppm)

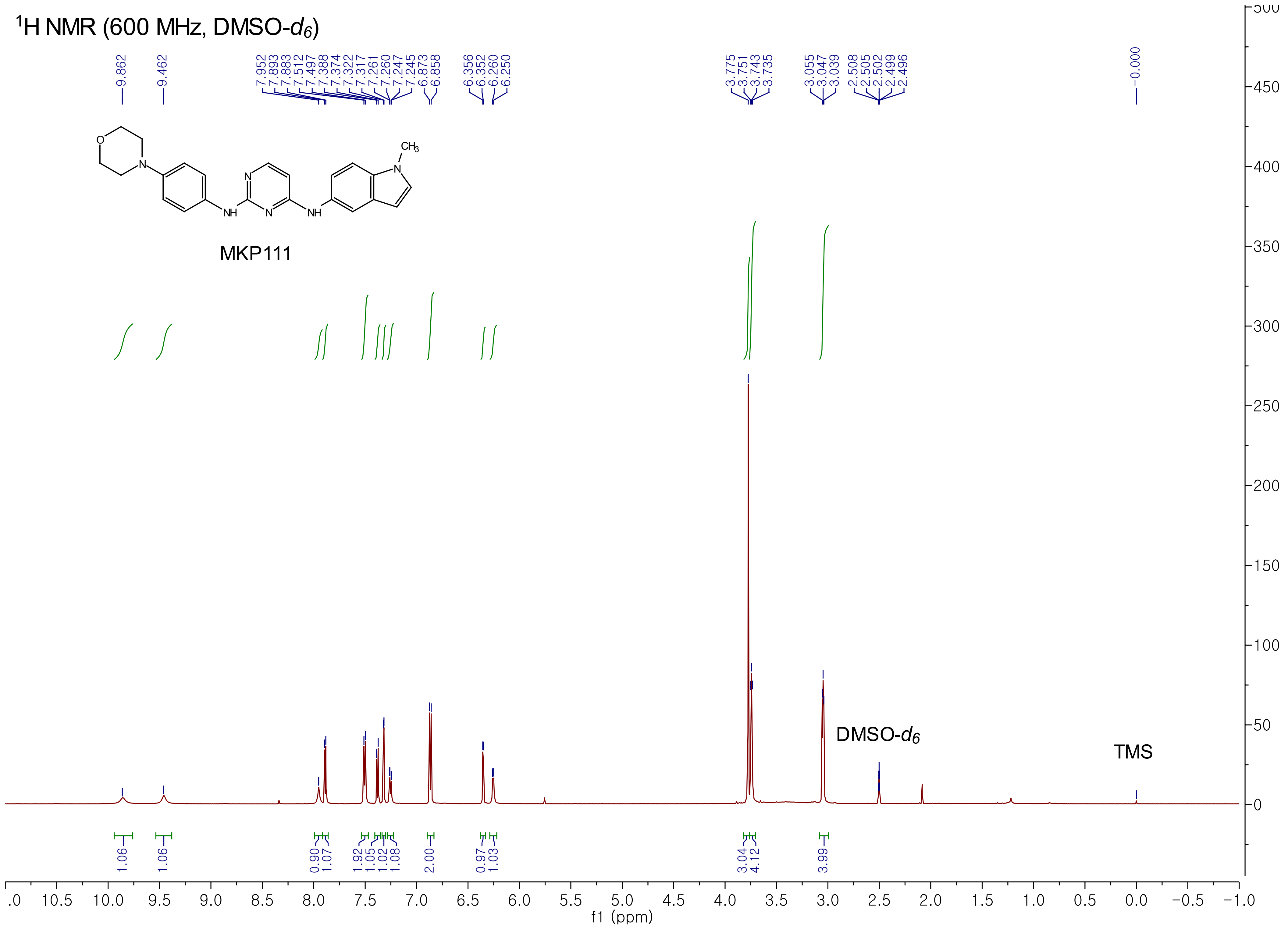

1H NMR (600 MHz, DMSO-d6)
MKP111
DMSO-d6
TMS
f1 (ppm)

^{13}C NMR (150 MHz, DMSO-d_6)

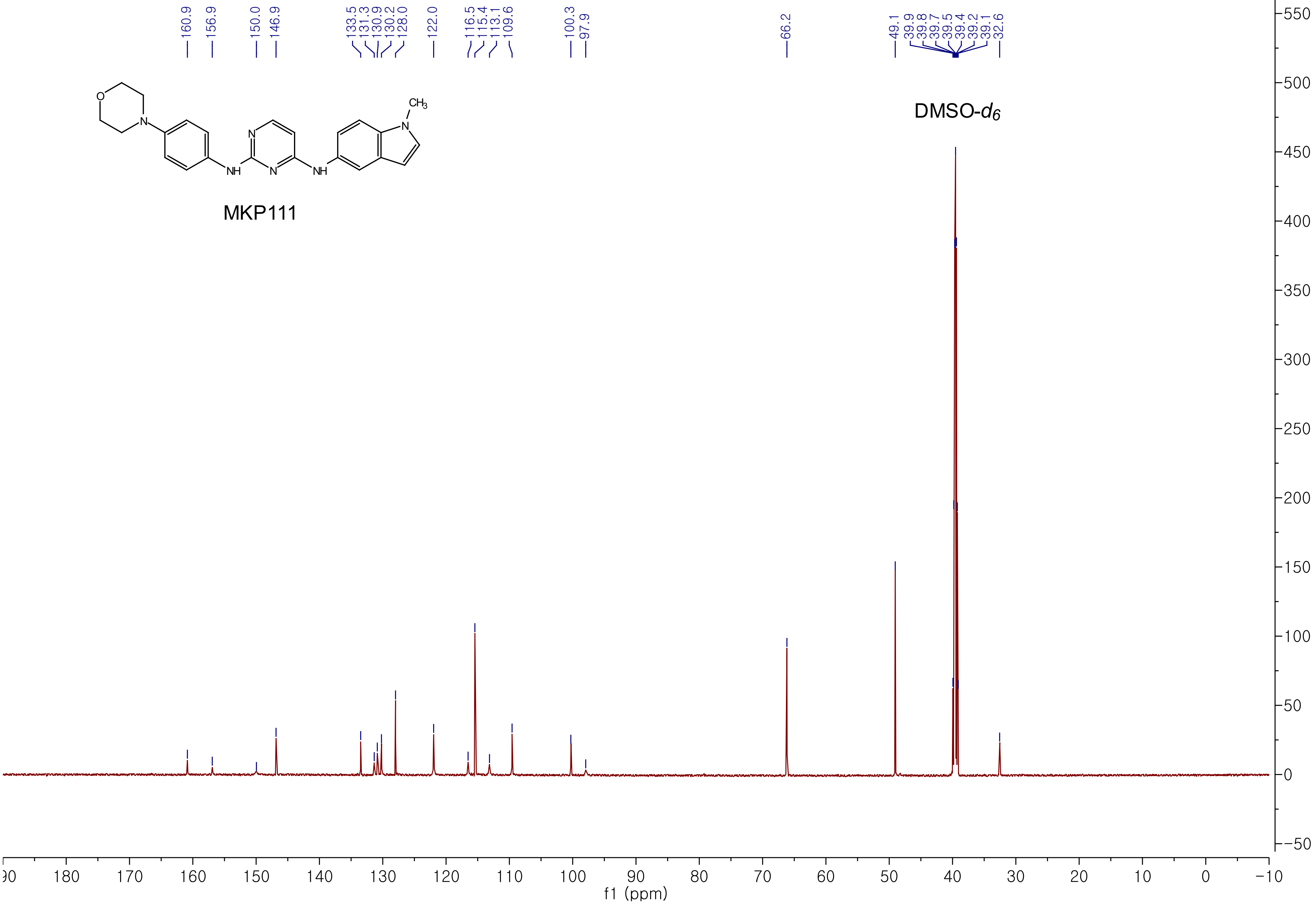

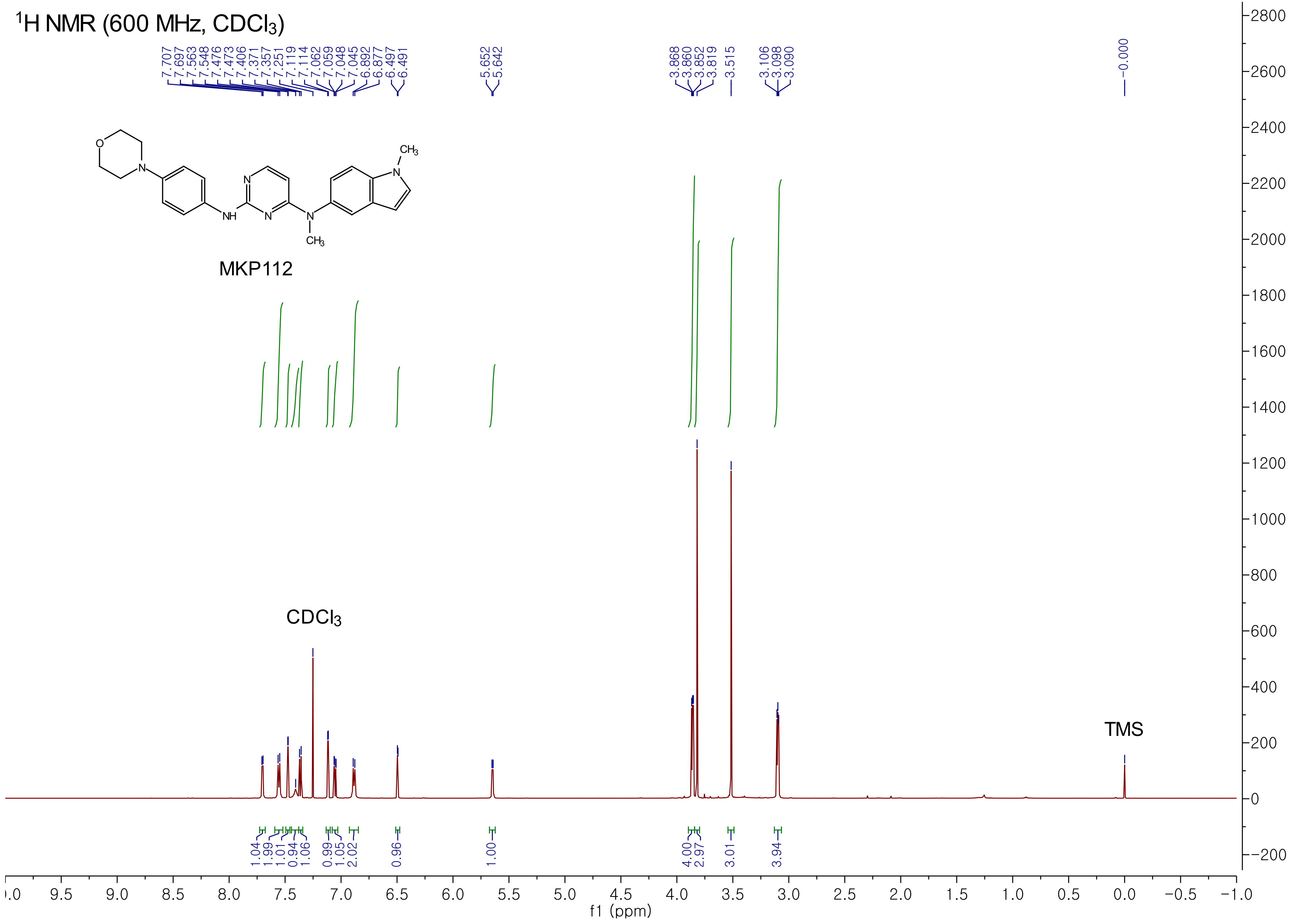

¹H NMR (600 MHz, CDCl3)
MKP112
CDCl3
TMS
f1 (ppm)

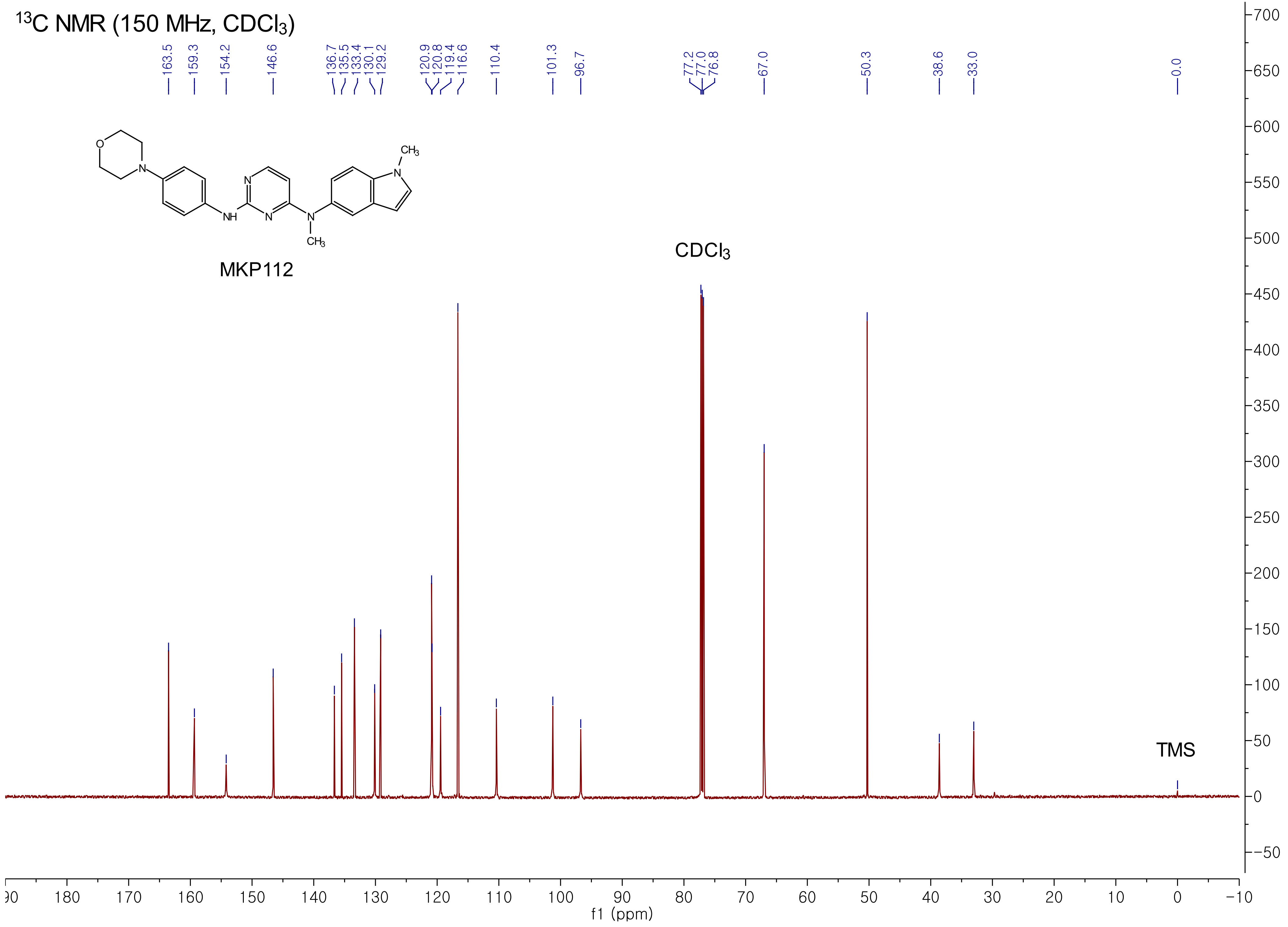

^{13}C NMR (150 MHz, $CDCl_3$)
163.5
159.3
154.2
146.6
136.7
135.5
133.4
130.1
129.2
120.9
120.8
119.4
116.6
110.4
101.3
96.7
77.2
77.0
76.8
67.0
50.3
38.6
33.0
0.0
O
N
NH
N
N
N
CH_3
CH_3
MKP112
$CDCl_3$
TMS
f1 (ppm)

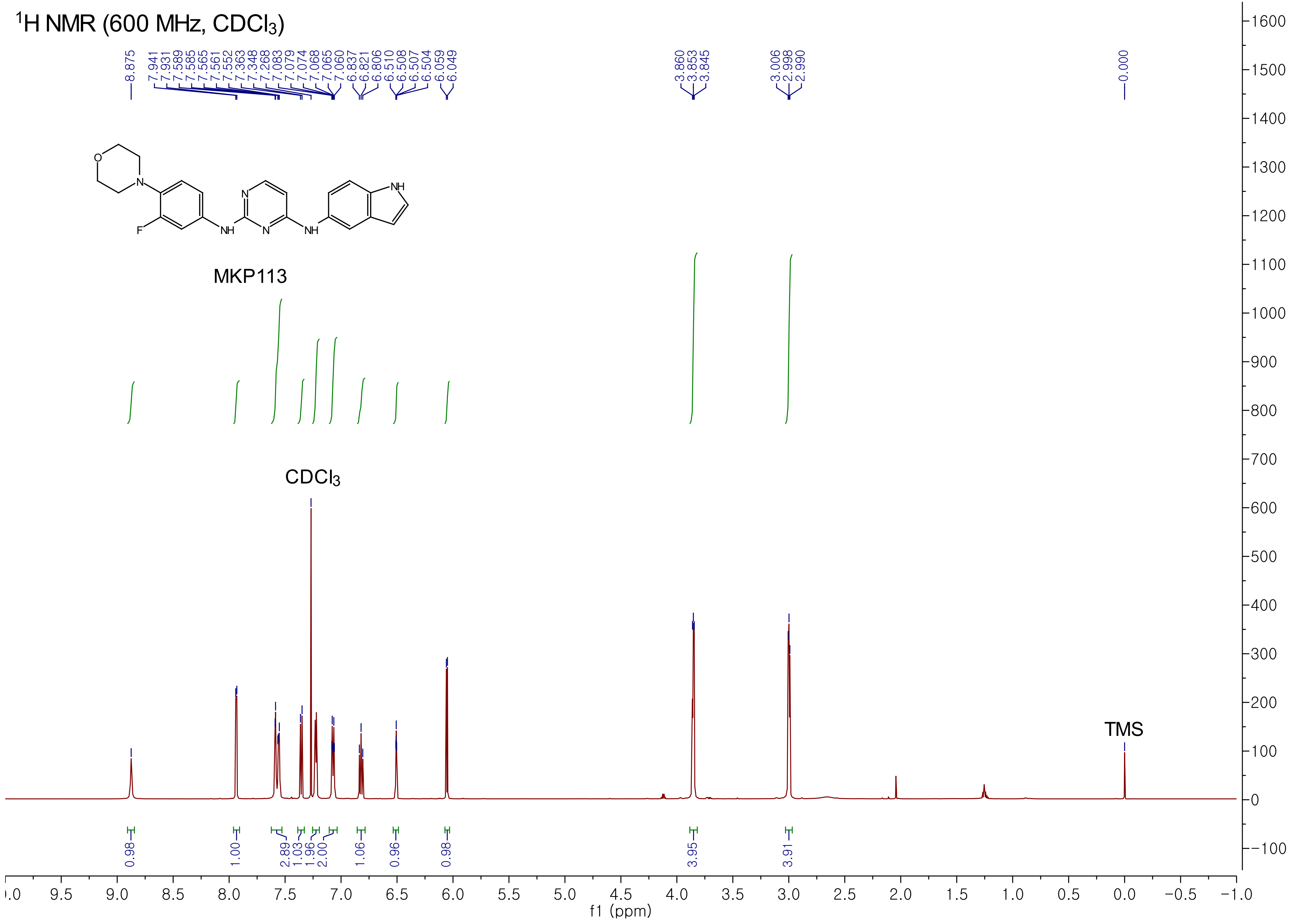

^{1}H NMR (600 MHz, CDCl$_3$)
8.875
7.941 7.931 7.589 7.585 7.565 7.561 7.552 7.363 7.348 7.268 7.083 7.079 7.074 7.068 7.065 7.060 6.837 6.821 6.806 6.510 6.508 6.507 6.504 6.059 6.049
3.860 3.853 3.845
3.006 2.998 2.990
0.000
O
N
F
NH
N
N
NH
NH
MKP113
CDCl$_3$
TMS
0.98 1.00 2.89 1.03 1.96 2.00 1.06 0.96 0.98 3.95 3.91
f1 (ppm)

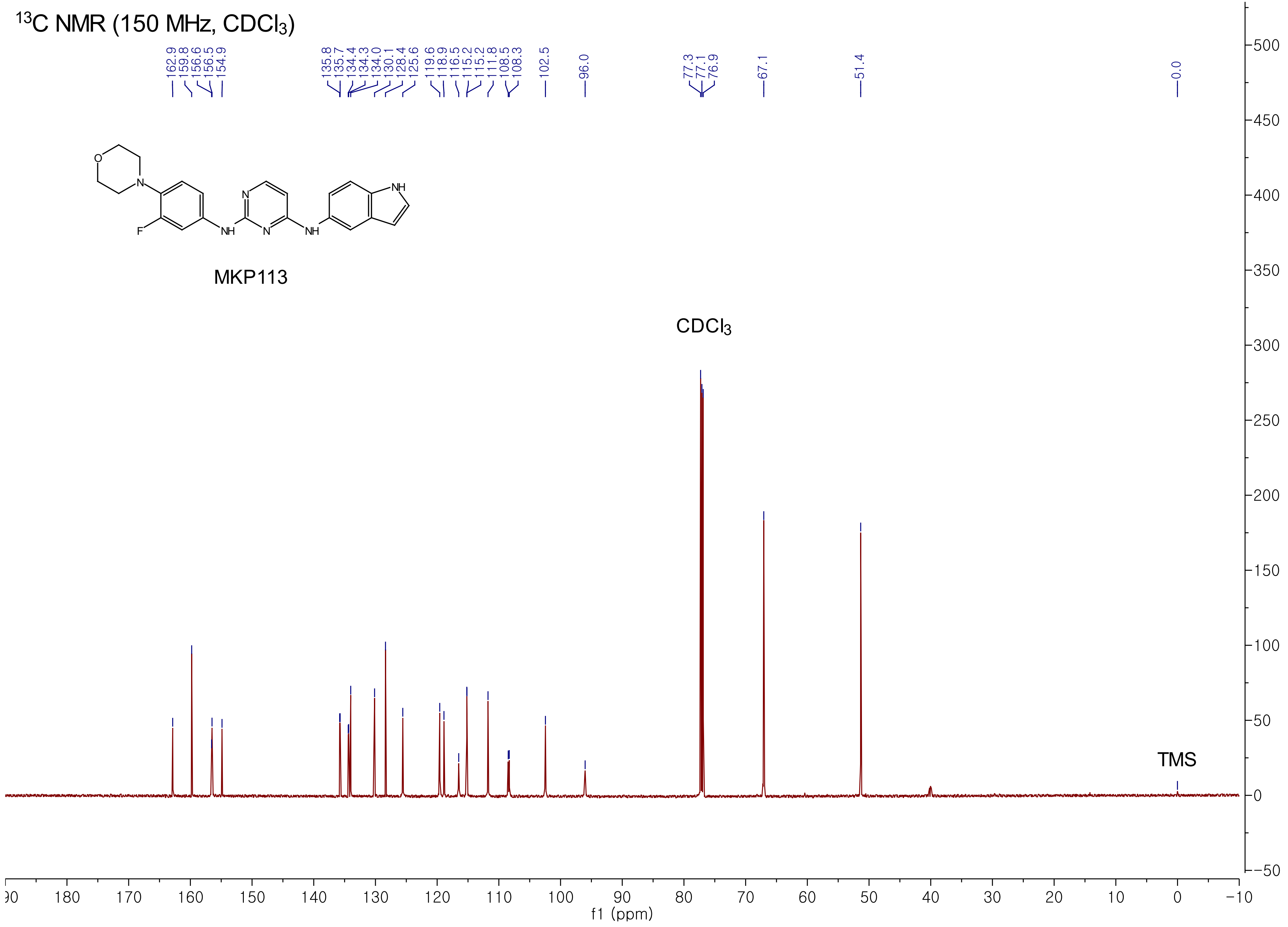

13C NMR (150 MHz, CDCl3)
162.9
159.8
156.6
156.5
154.9
135.8
135.7
134.4
134.3
134.0
130.1
128.4
125.6
119.6
118.9
116.5
115.2
115.2
111.8
108.5
108.3
102.5
96.0
77.3
77.1
76.9
67.1
51.4
0.0
O
N
F
NH
N
N
NH
NH
MKP113
CDCl3
TMS
f1 (ppm)

^{1}H NMR (600 MHz, $CDCl_3$)

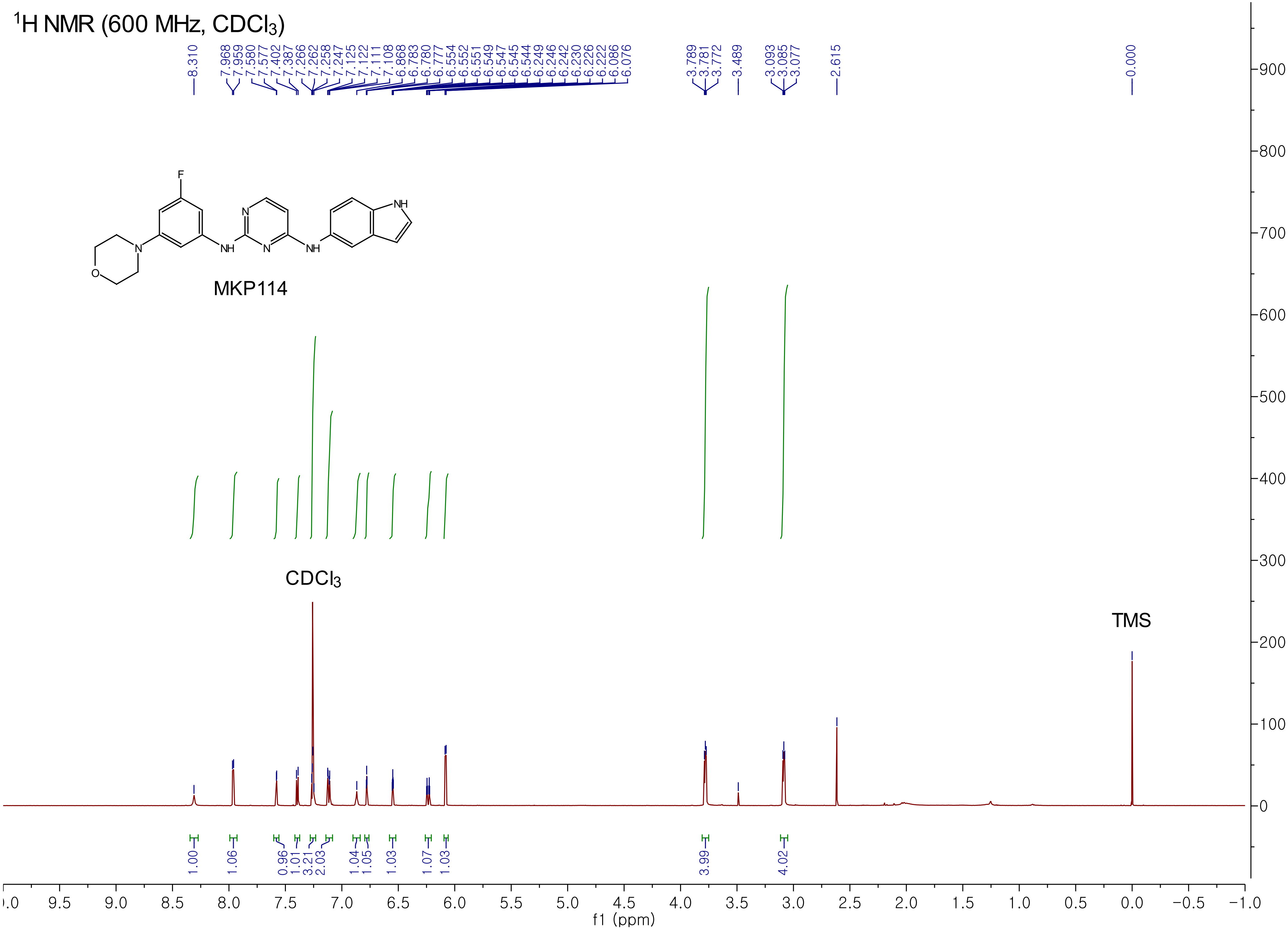

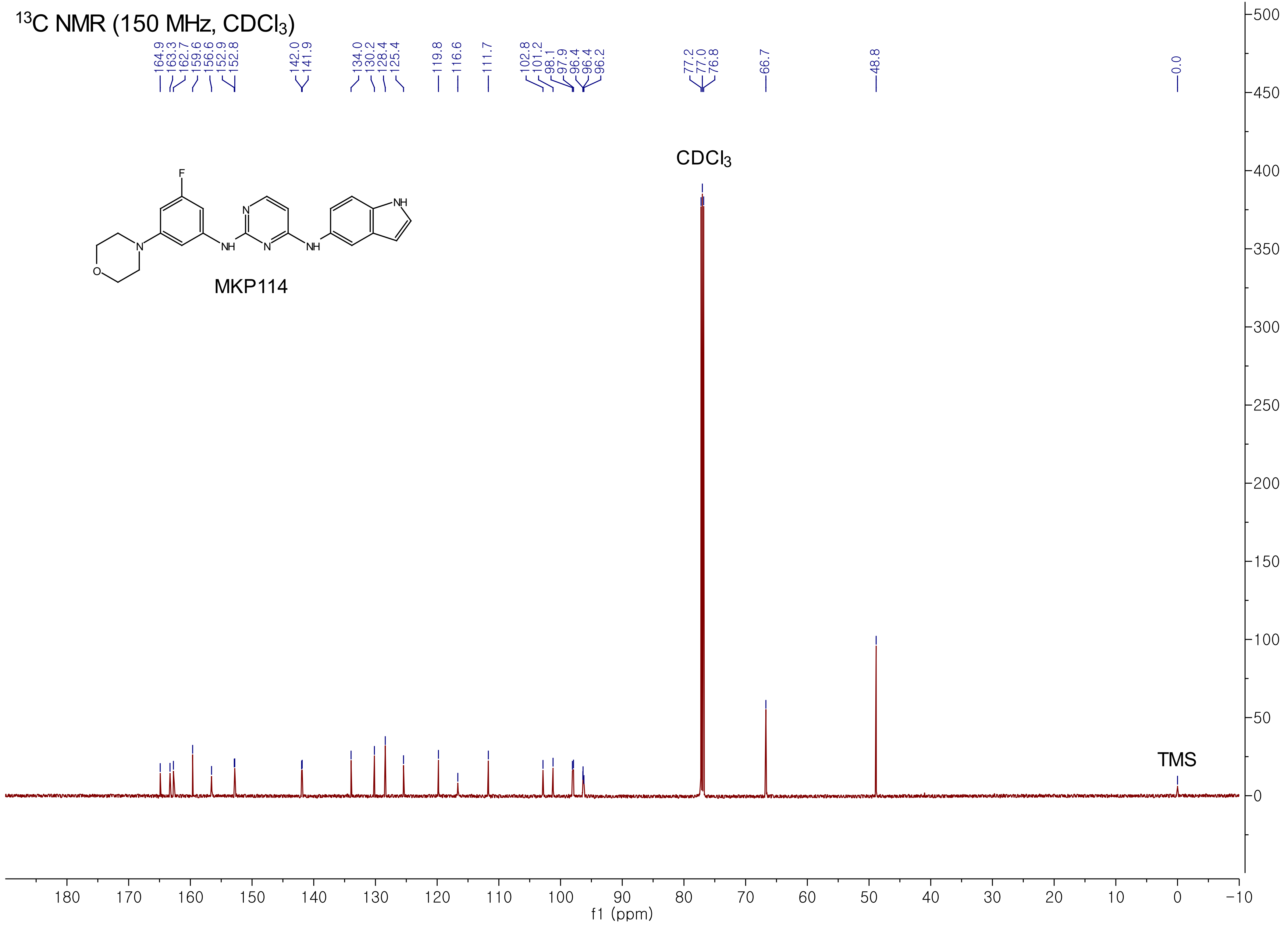

^{13}C NMR (150 MHz, CDCl$_3$)
164.9
163.3
162.7
159.6
156.6
152.9
152.8
142.0
141.9
134.0
130.2
128.4
125.4
119.8
116.6
111.7
102.8
101.2
98.1
97.9
96.4
96.4
96.2
77.2
77.0
76.8
66.7
48.8
0.0
CDCl$_3$
MKP114
TMS
f1 (ppm)

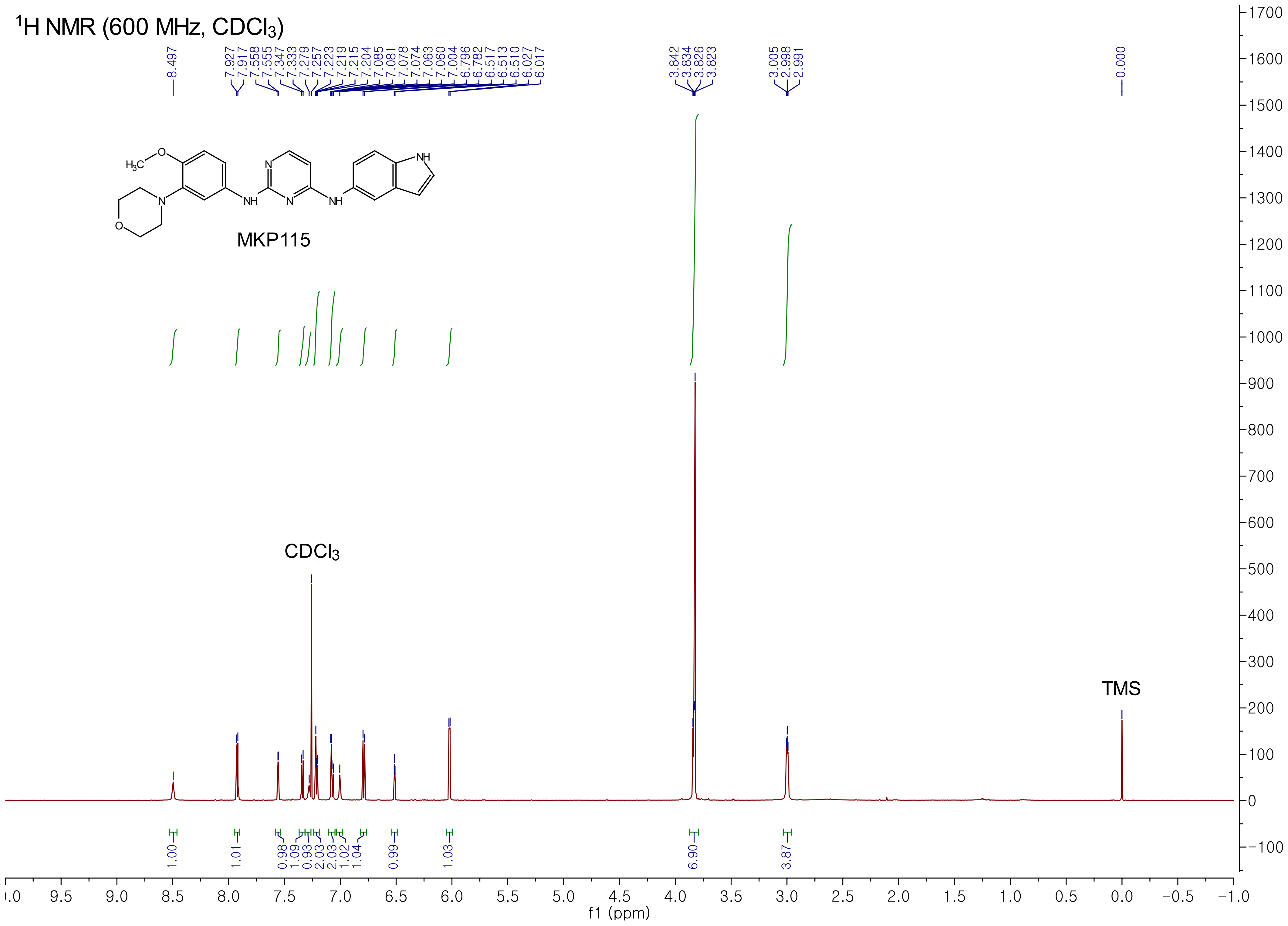

^{1}H NMR (600 MHz, CDCl$_3$)
8.497
7.927
7.917
7.558
7.555
7.347
7.333
7.279
7.257
7.223
7.219
7.215
7.204
7.085
7.081
7.078
7.074
7.063
7.060
7.004
6.796
6.782
6.517
6.513
6.510
6.027
6.017
3.842
3.834
3.826
3.823
3.005
2.998
2.991
0.000
H3C
O
N
NH
N
N
NH
NH
O
MKP115
CDCl3
TMS
1.00
1.01
0.98
1.09
0.93
2.03
2.03
1.02
1.04
0.99
1.03
6.90
3.87
f1 (ppm)

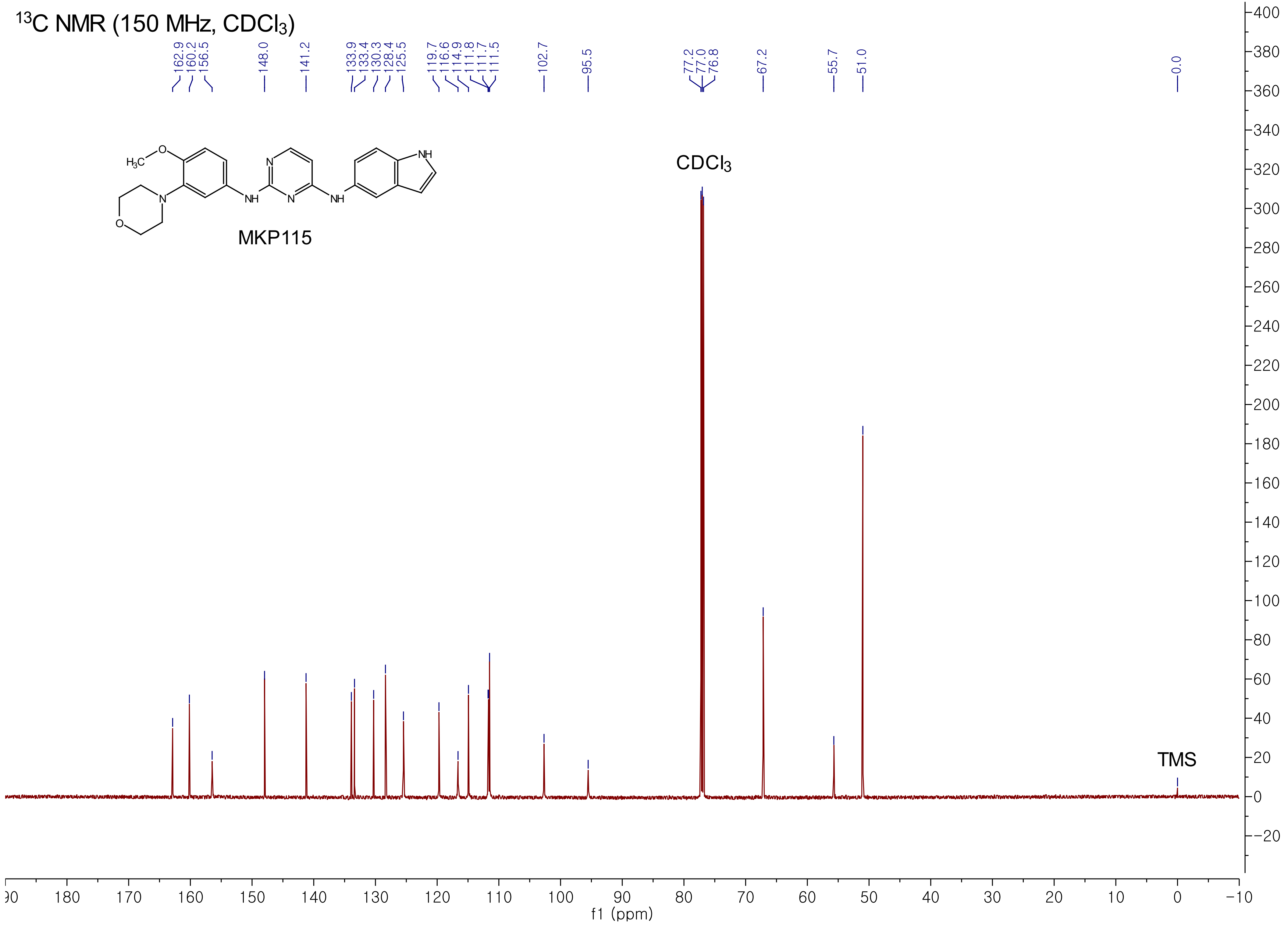

^{13}C NMR (150 MHz, $CDCl_3$)
162.9
160.2
156.5
148.0
141.2
133.9
133.4
130.3
128.4
125.5
119.7
116.6
114.9
111.8
111.7
111.5
102.7
95.5
77.2
77.0
76.8
67.2
55.7
51.0
0.0
$CDCl_3$
TMS
f1 (ppm)

MKP115

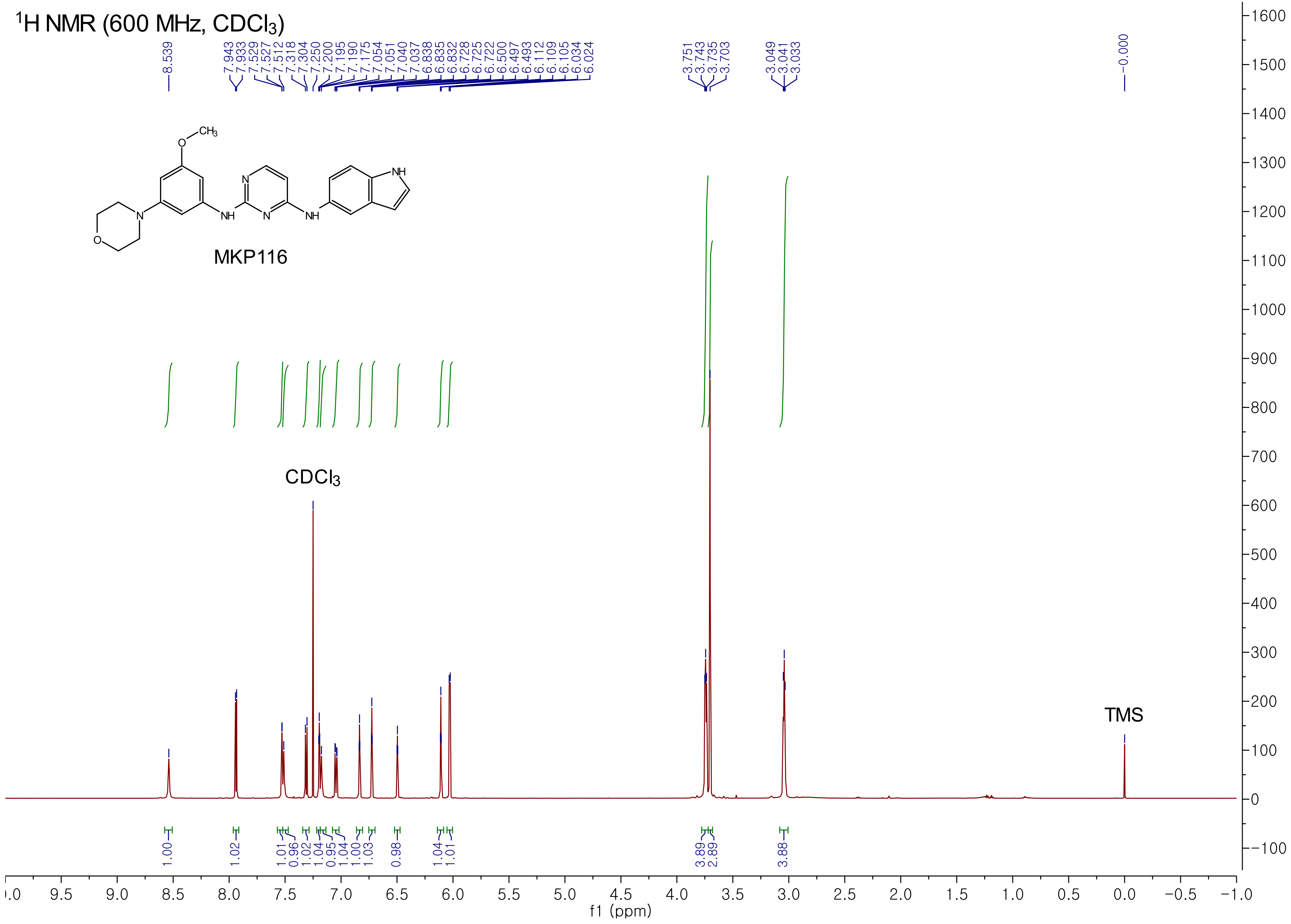

1H NMR (600 MHz, CDCl3)
8.539
7.943
7.933
7.529
7.527
7.512
7.318
7.304
7.250
7.200
7.195
7.190
7.175
7.054
7.051
7.040
7.037
6.838
6.835
6.832
6.728
6.725
6.722
6.500
6.497
6.493
6.112
6.109
6.105
6.034
6.024
3.751
3.743
3.735
3.703
3.049
3.041
3.033
-0.000
MKP116
CDCl3
TMS
1.00
1.02
1.01
0.96
1.02
1.04
0.95
1.04
1.00
1.03
0.98
1.04
1.01
3.89
2.89
3.88
f1 (ppm)

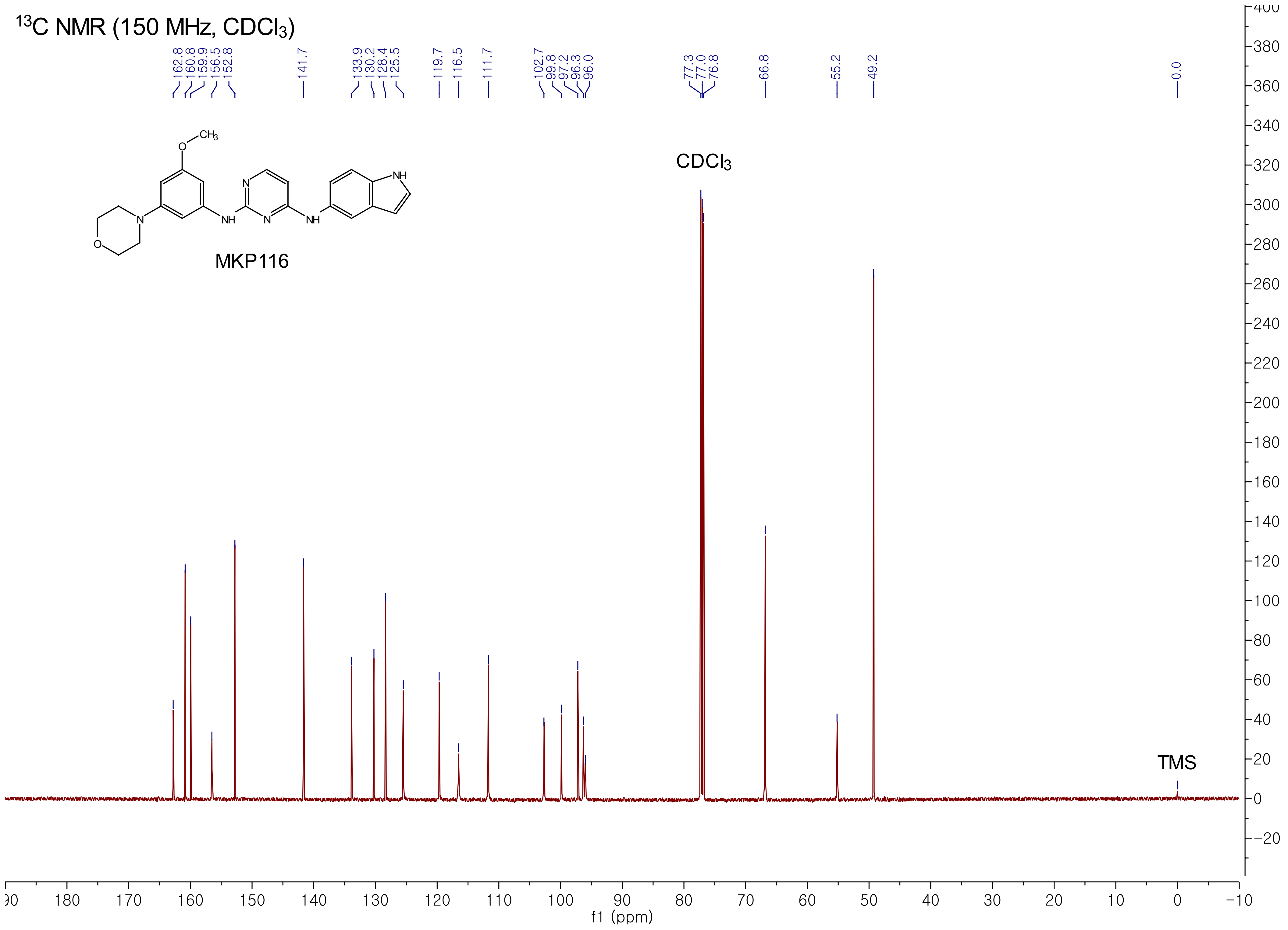

^{13}C NMR (150 MHz, CDCl$_3$)
162.8
160.8
159.9
156.5
152.8
141.7
133.9
130.2
128.4
125.5
119.7
116.5
111.7
102.7
99.8
97.2
96.3
96.0
77.3
77.0
76.8
66.8
55.2
49.2
0.0
CDCl$_3$
TMS
MKP116
f1 (ppm)

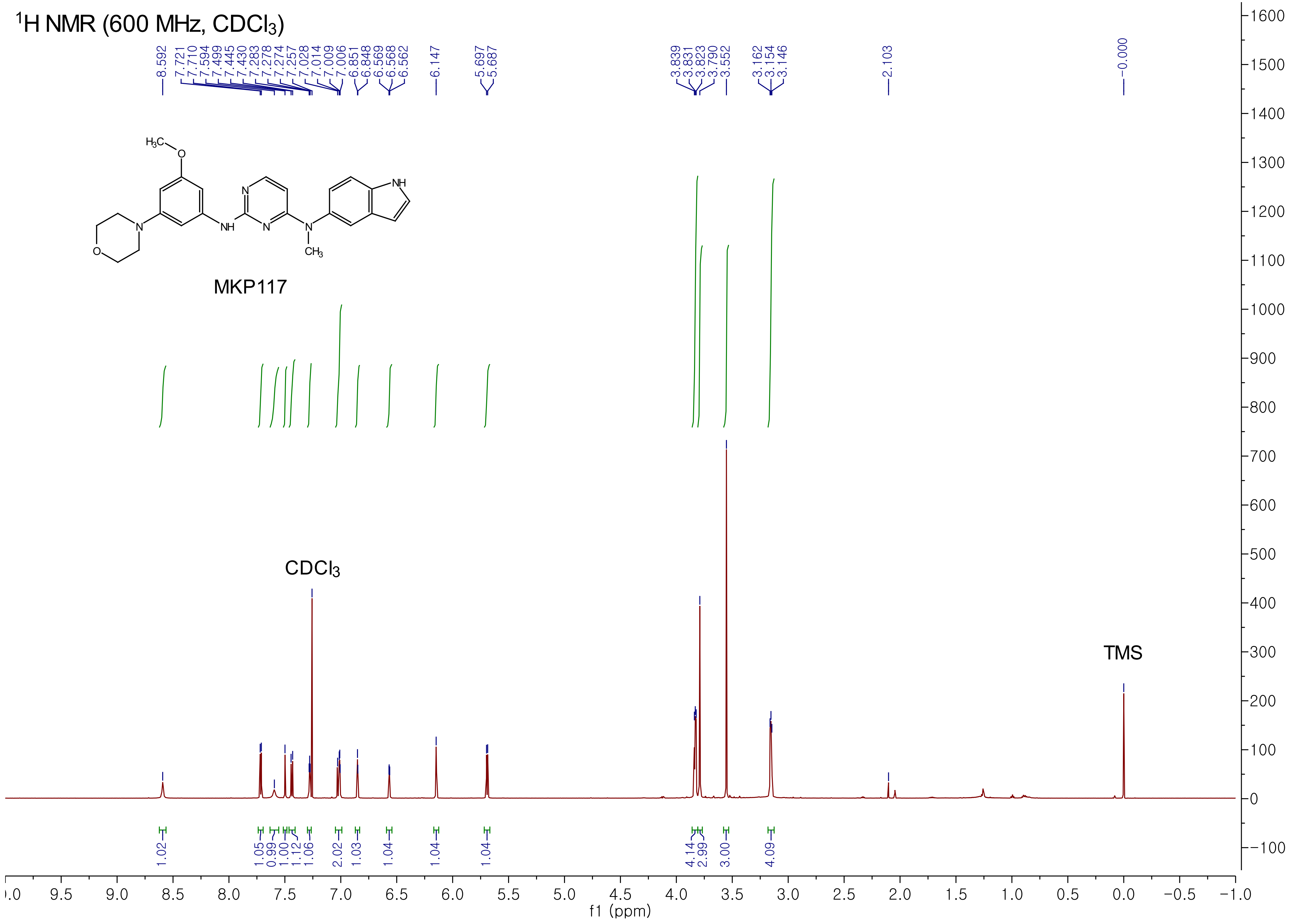

1H NMR (600 MHz, CDCl3)
8.592
7.721
7.710
7.594
7.499
7.445
7.430
7.283
7.278
7.274
7.257
7.028
7.014
7.009
7.006
6.851
6.848
6.569
6.568
6.562
6.147
5.697
5.687
3.839
3.831
3.823
3.790
3.552
3.162
3.154
3.146
2.103
-0.000
H3C
O
N
NH
N
N
N
NH
CH3
O
MKP117
CDCl3
TMS
1.02
1.05
0.99
1.00
1.12
1.06
2.02
1.03
1.04
1.04
1.04
4.14
2.99
3.00
4.09
f1 (ppm)

^{13}C NMR (150 MHz, $CDCl_3$)

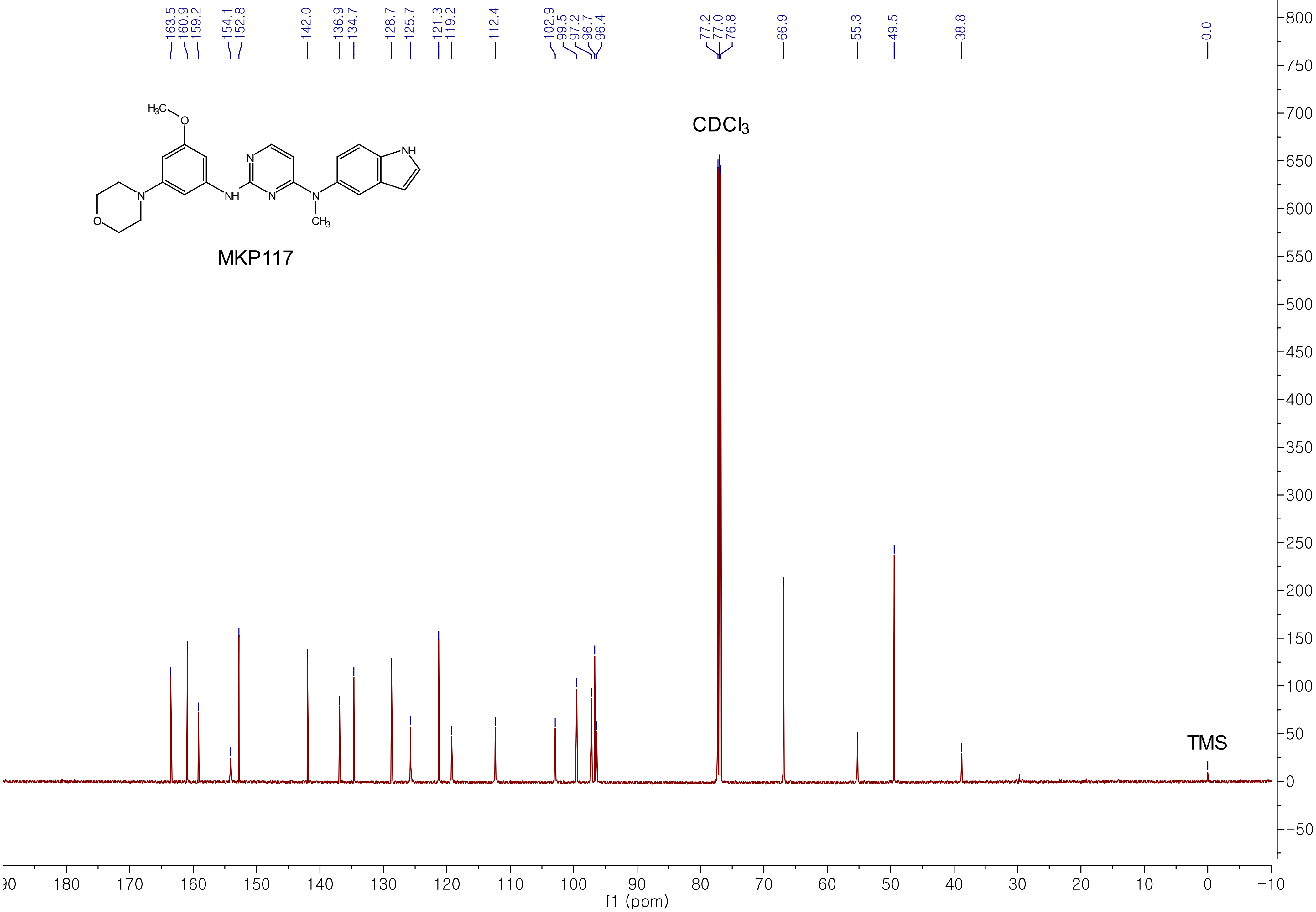

MKP117

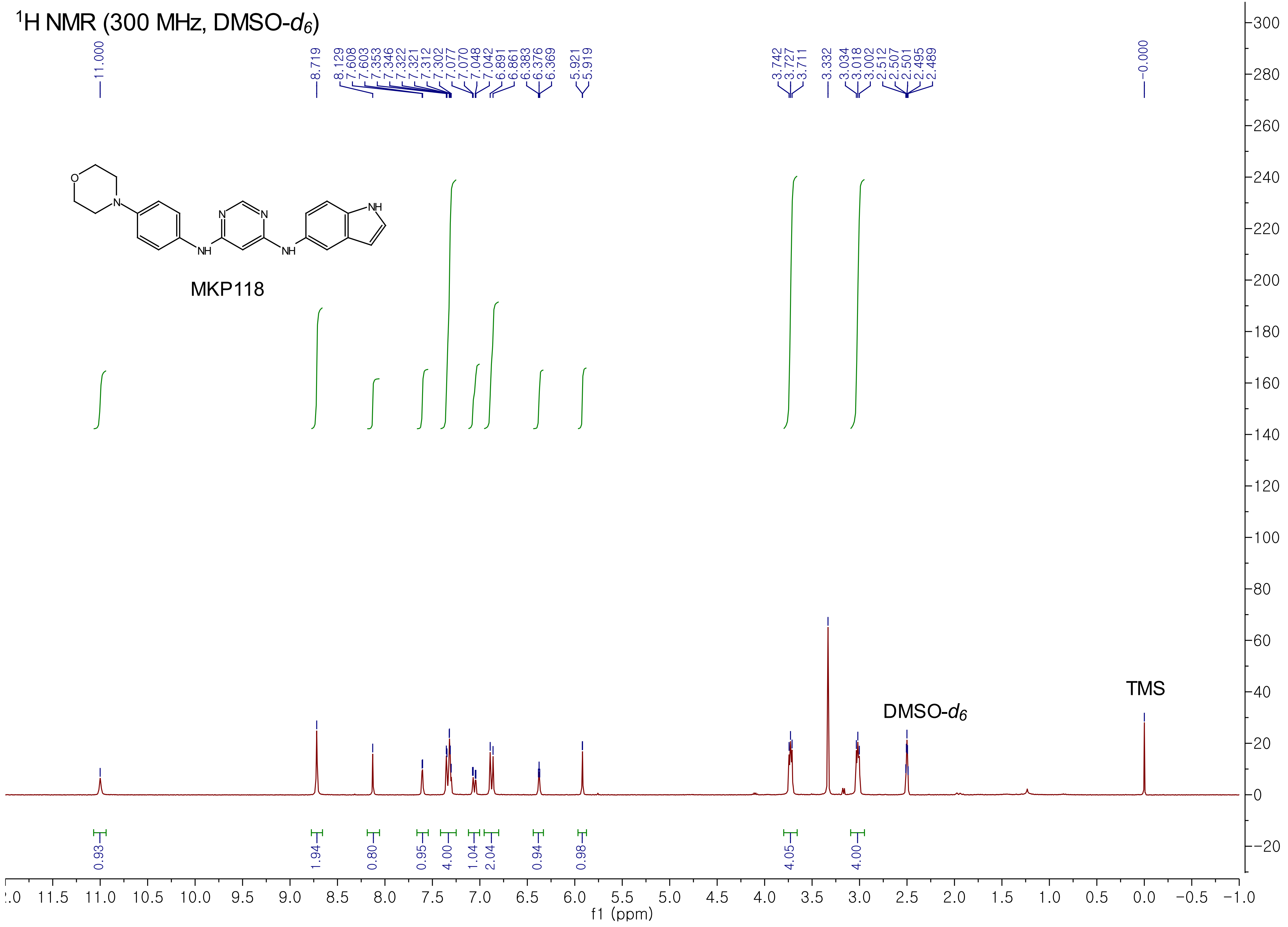

1H NMR (300 MHz, DMSO-d6)
MKP118
DMSO-d6
TMS
f1 (ppm)

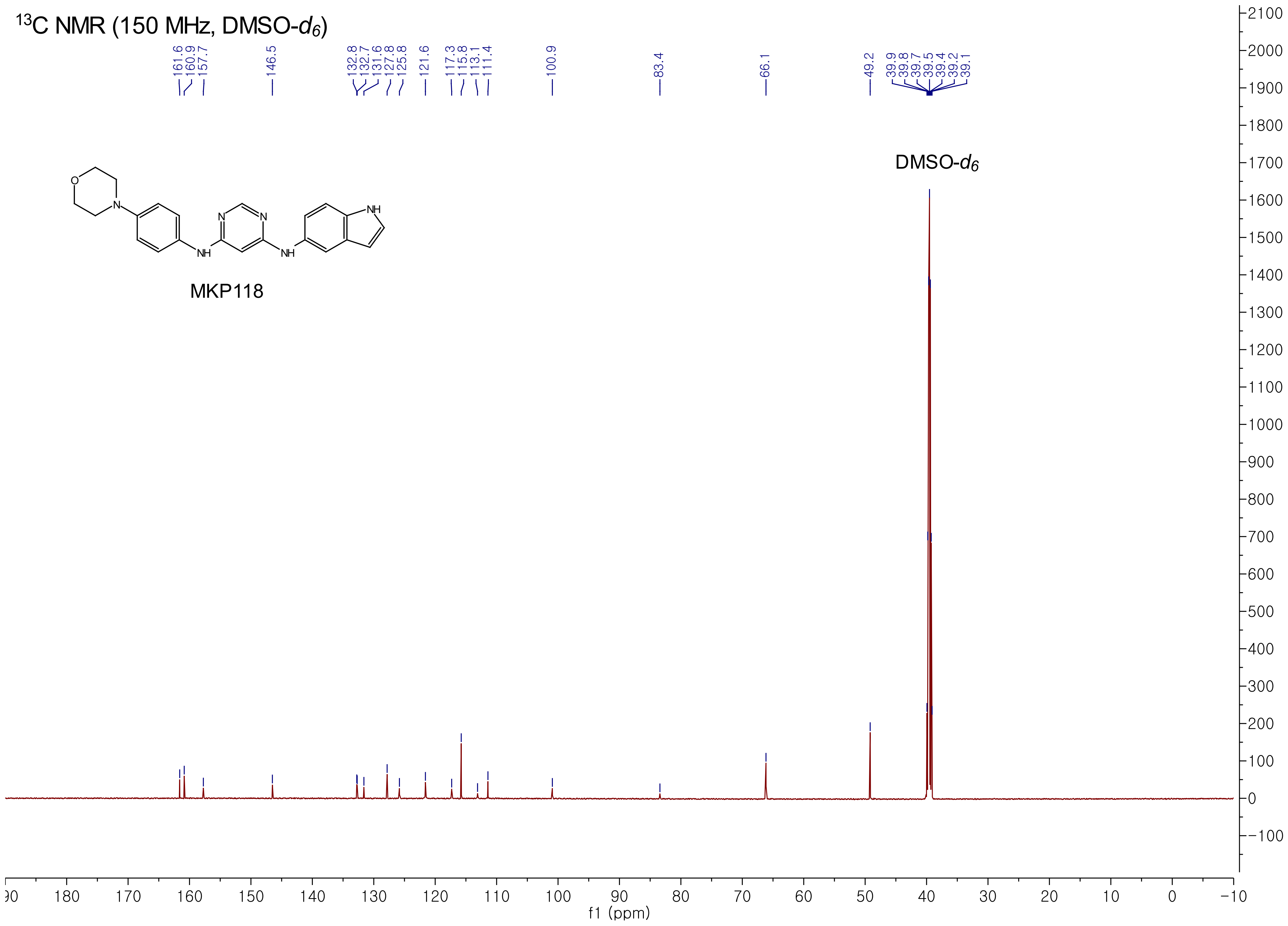

13C NMR (150 MHz, DMSO-d6)
161.6
160.9
157.7
146.5
132.8
132.7
131.6
127.8
125.8
121.6
117.3
115.8
113.1
111.4
100.9
83.4
66.1
49.2
39.9
39.8
39.7
39.5
39.4
39.2
39.1
DMSO-d6
O
N
N
N
NH
NH
NH
MKP118
f1 (ppm)

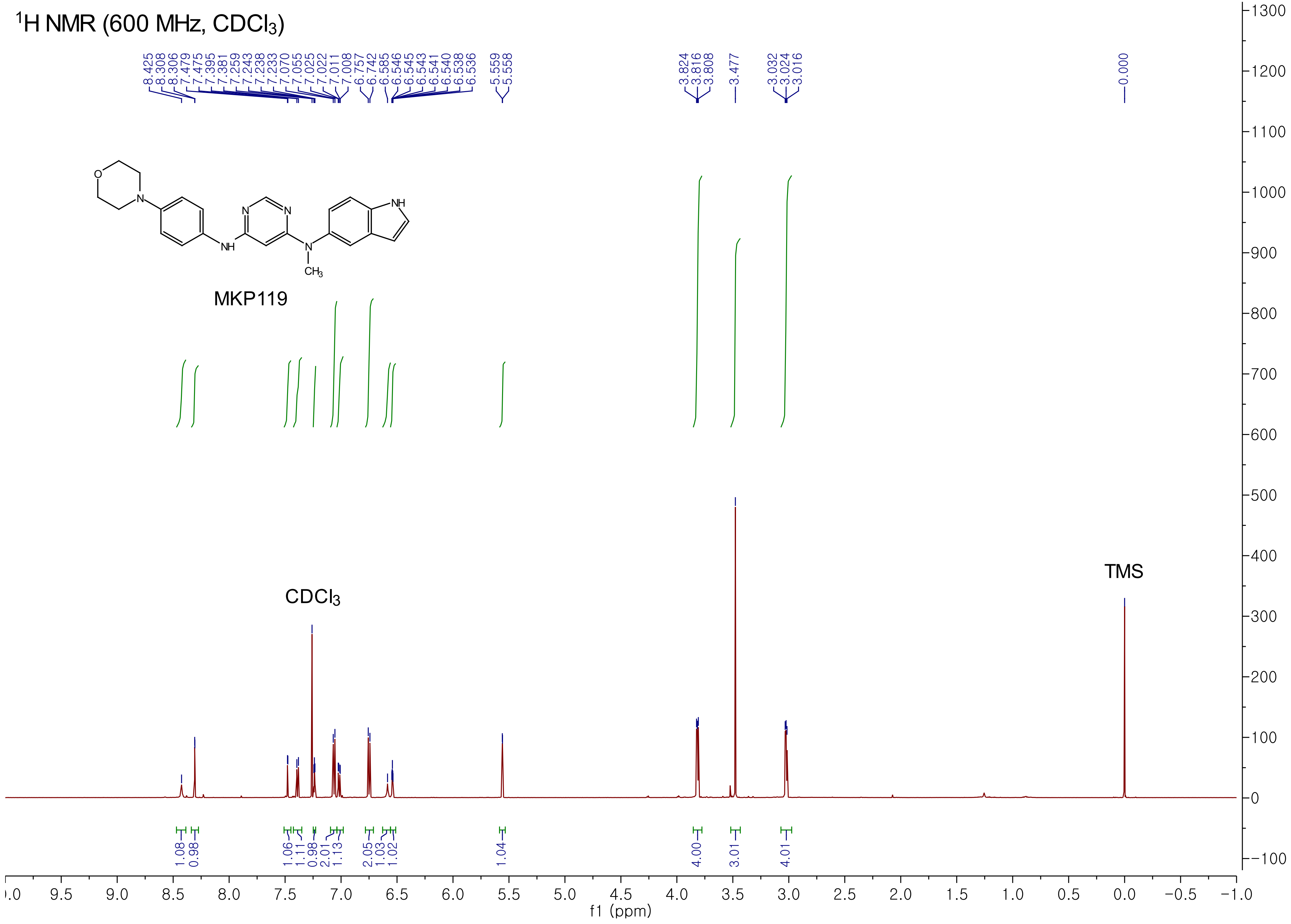

¹H NMR (600 MHz, CDCl3)
8.425
8.308
8.306
7.479
7.475
7.395
7.381
7.259
7.243
7.238
7.233
7.070
7.055
7.025
7.022
7.011
7.008
6.757
6.742
6.585
6.546
6.545
6.543
6.541
6.540
6.538
6.536
5.559
5.558
3.824
3.816
3.808
3.477
3.032
3.024
3.016
0.000
MKP119
CDCl3
TMS
1.08
0.98
1.06
1.11
0.98
2.01
1.13
2.05
1.03
1.02
1.04
4.00
3.01
4.01
f1 (ppm)

^{13}C NMR (150 MHz, $CDCl_3$)

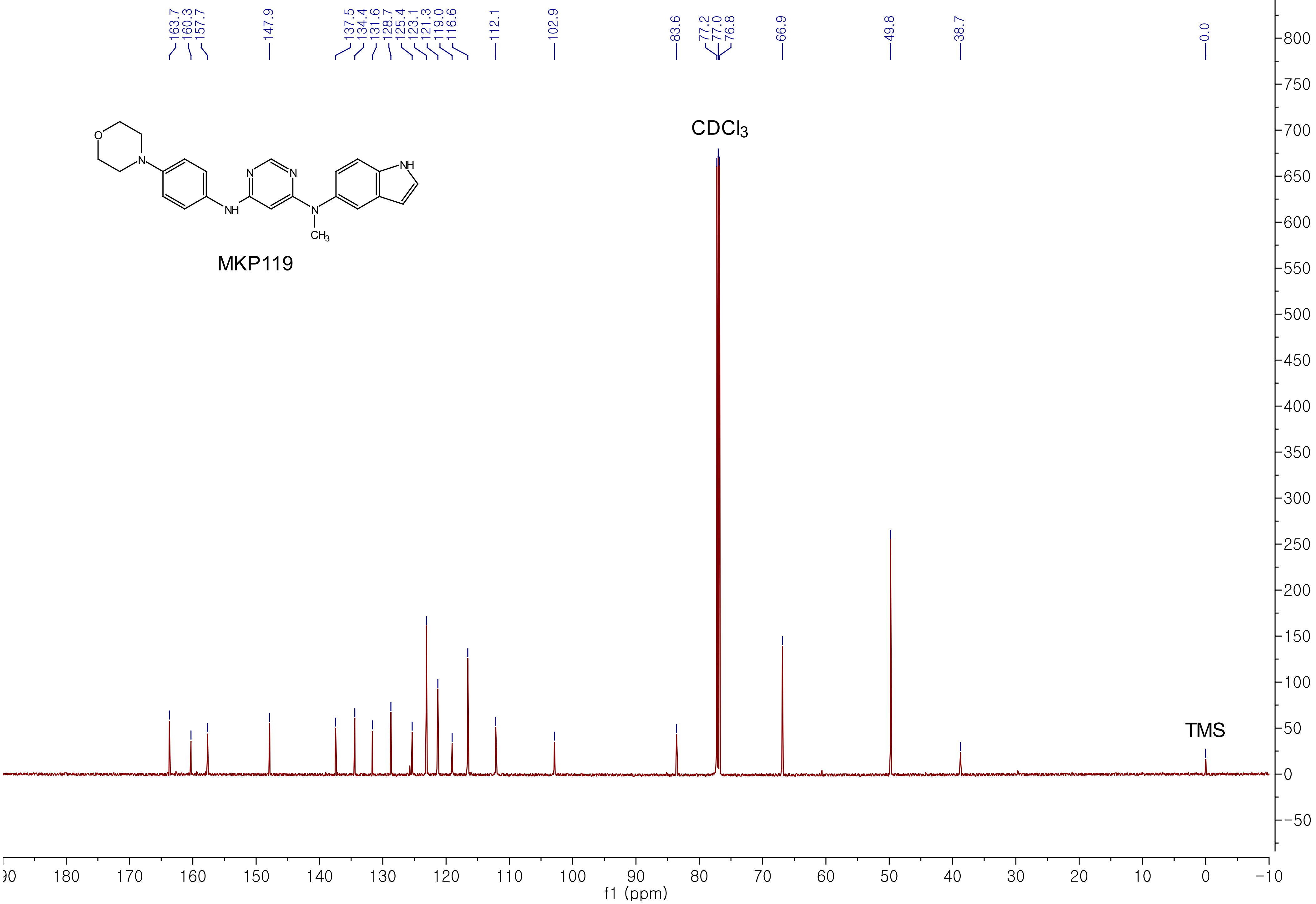

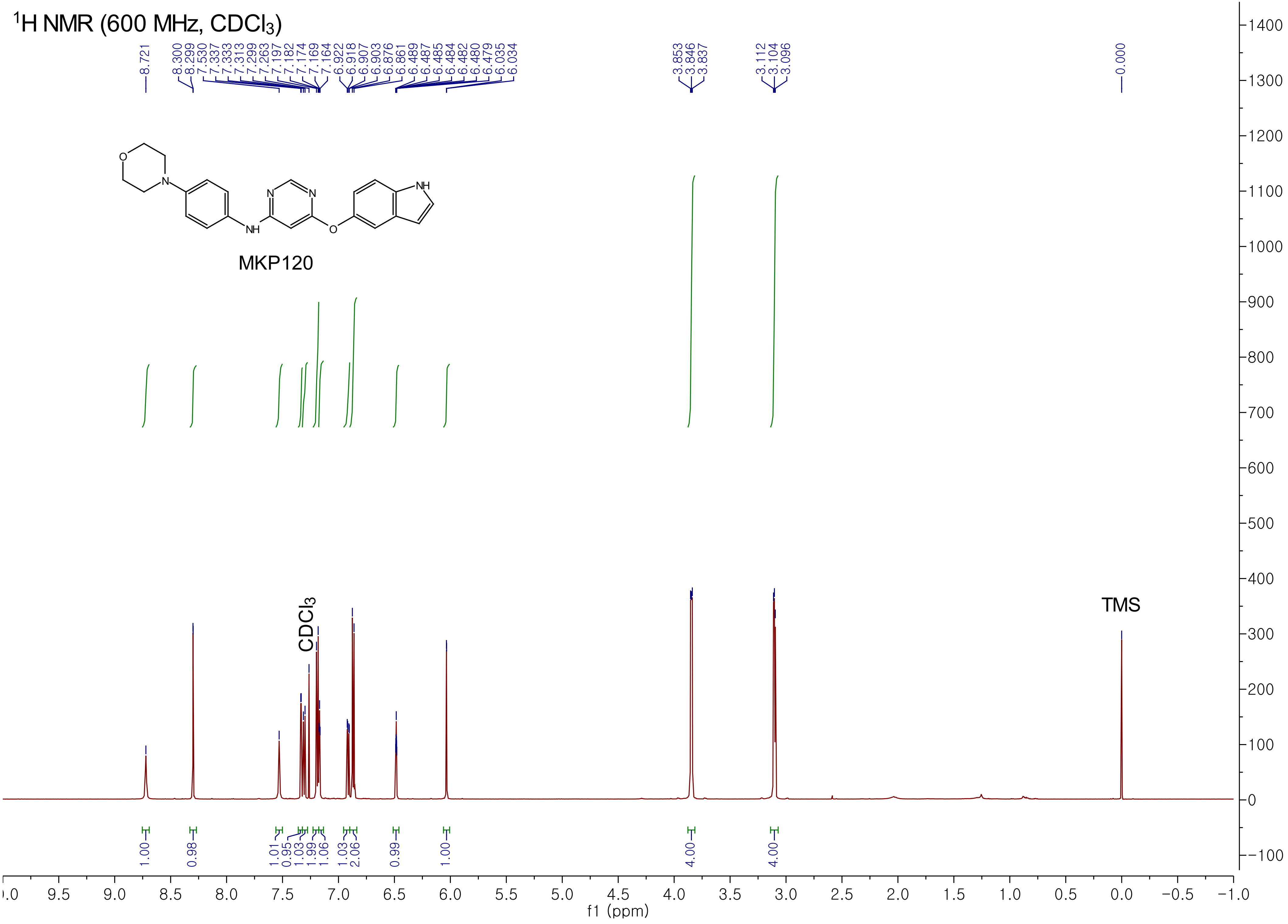

1H NMR (600 MHz, CDCl3)
8.721
8.300
8.299
7.530
7.337
7.333
7.313
7.299
7.263
7.197
7.182
7.174
7.169
7.164
6.922
6.918
6.907
6.903
6.876
6.861
6.489
6.487
6.485
6.484
6.482
6.480
6.479
6.035
6.034
3.853
3.846
3.837
3.112
3.104
3.096
0.000
MKP120
CDCl3
TMS
1.00
0.98
1.01
0.95
1.03
1.99
1.06
1.03
2.06
0.99
1.00
4.00
4.00
f1 (ppm)

^{13}C NMR (150 MHz, $CDCl_3$)

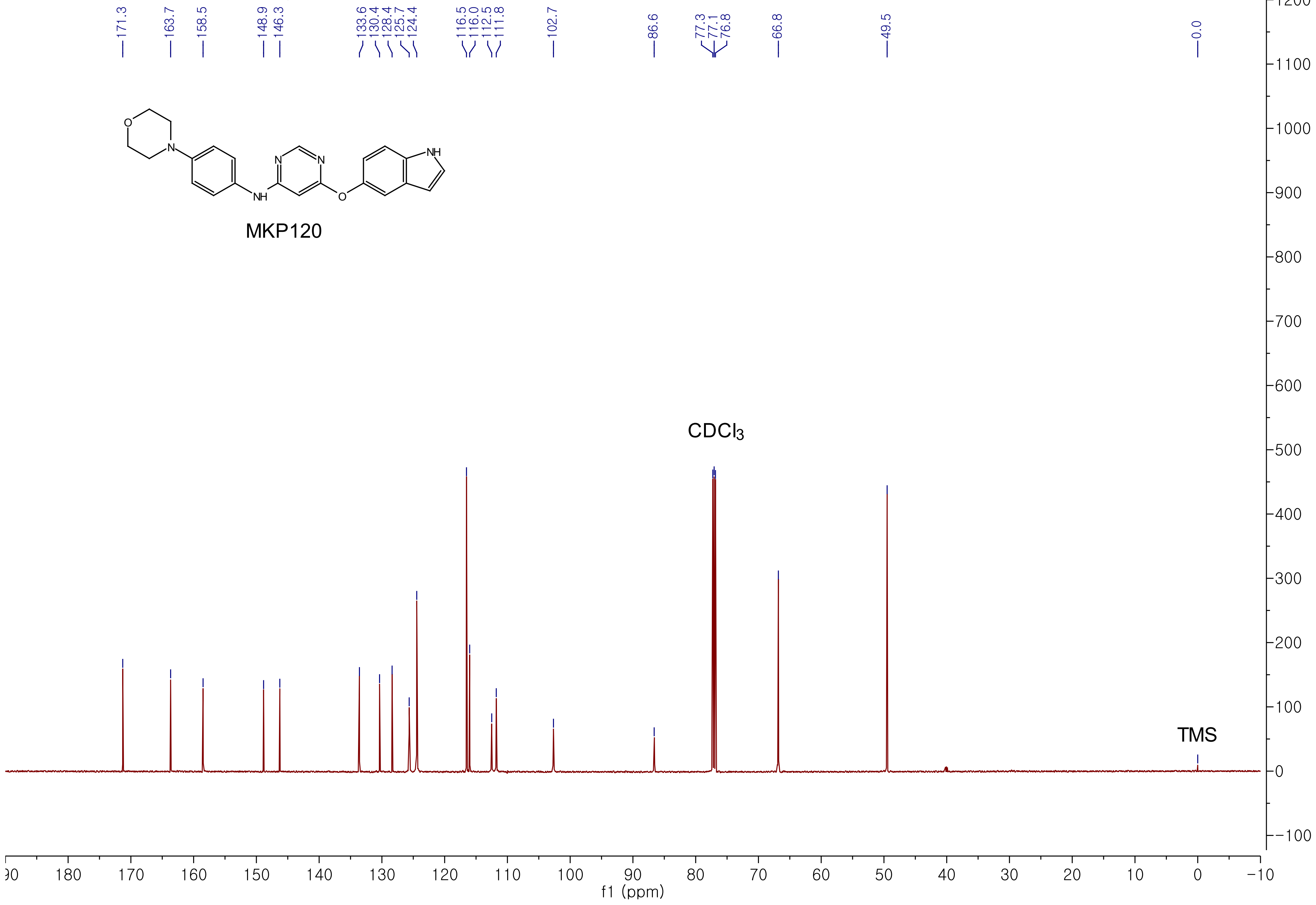

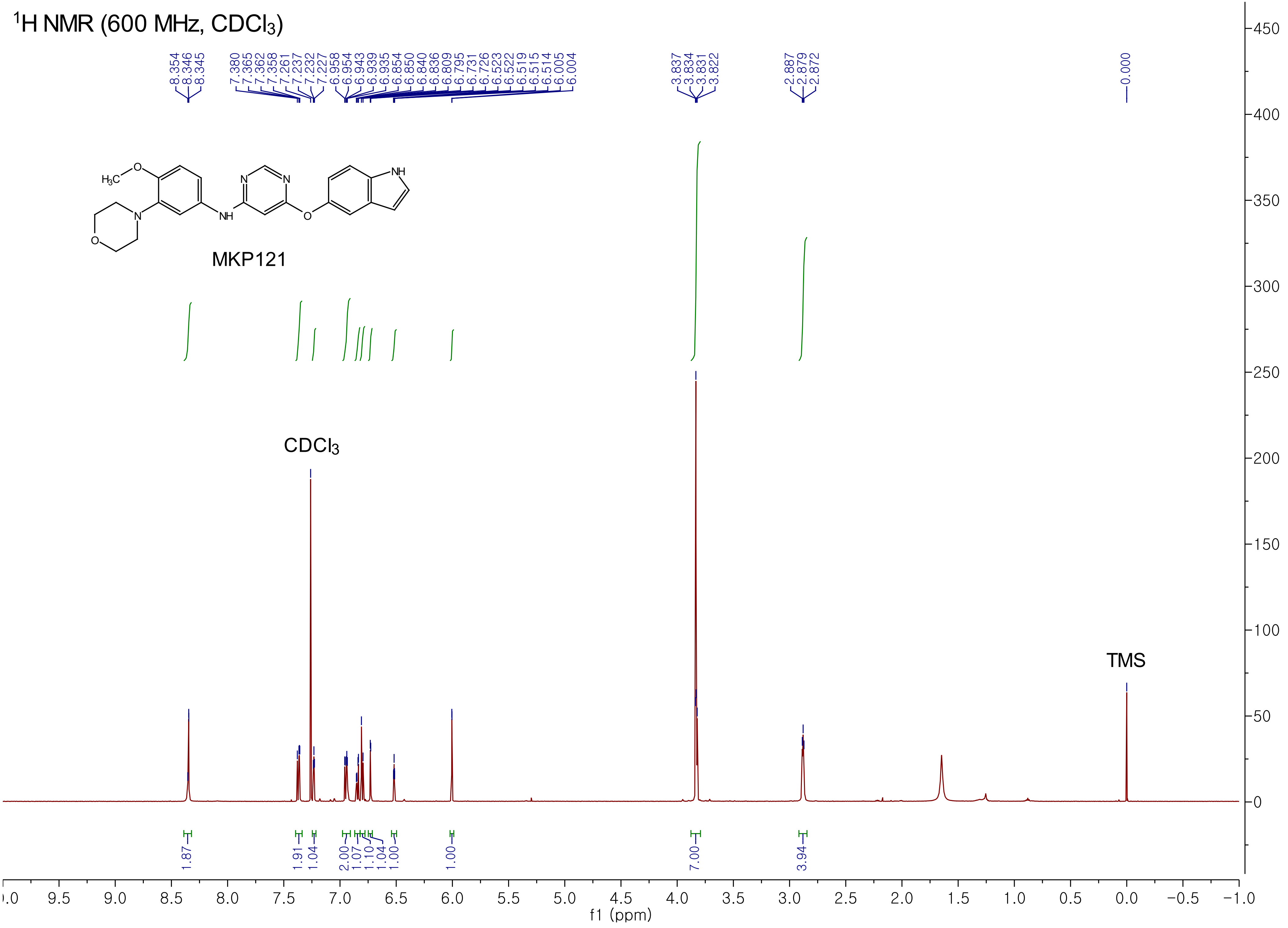

1H NMR (600 MHz, CDCl3)
8.354
8.346
8.345
7.380
7.365
7.362
7.358
7.261
7.237
7.232
7.227
6.958
6.954
6.943
6.939
6.935
6.854
6.850
6.840
6.836
6.809
6.795
6.731
6.726
6.523
6.522
6.519
6.515
6.514
6.005
6.004
3.837
3.834
3.831
3.822
2.887
2.879
2.872
0.000
H3C
O
N
NH
N
N
O
NH
O
MKP121
CDCl3
TMS
1.87
1.91
1.04
2.00
1.07
1.10
1.04
1.00
1.00
7.00
3.94
f1 (ppm)

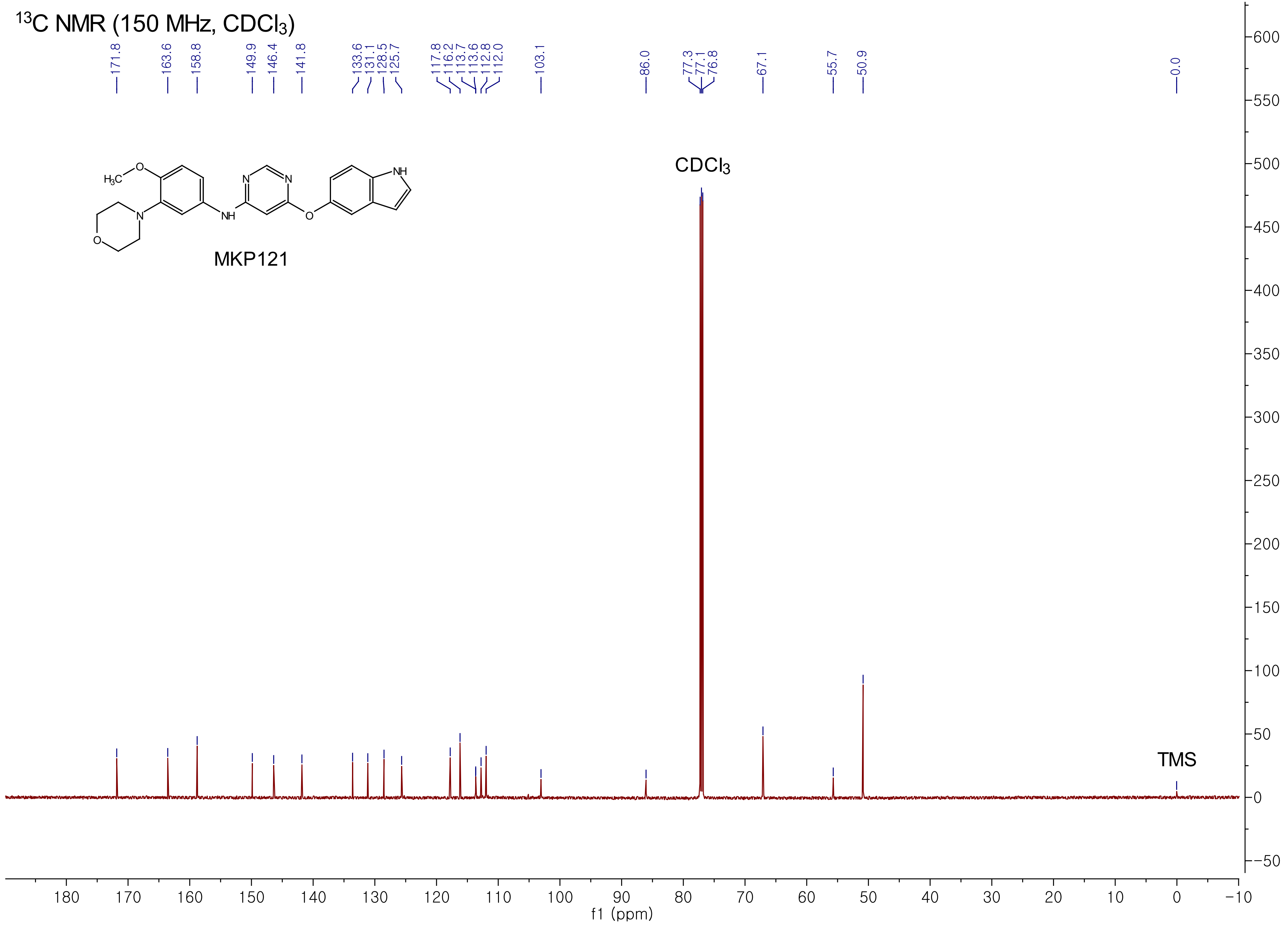

^{13}C NMR (150 MHz, $CDCl_3$)
171.8
163.6
158.8
149.9
146.4
141.8
133.6
131.1
128.5
125.7
117.8
116.2
113.7
113.6
112.8
112.0
103.1
86.0
77.3
77.1
76.8
67.1
55.7
50.9
0.0
H_3C
O
N
NH
MKP121
$CDCl_3$
TMS
f1 (ppm)

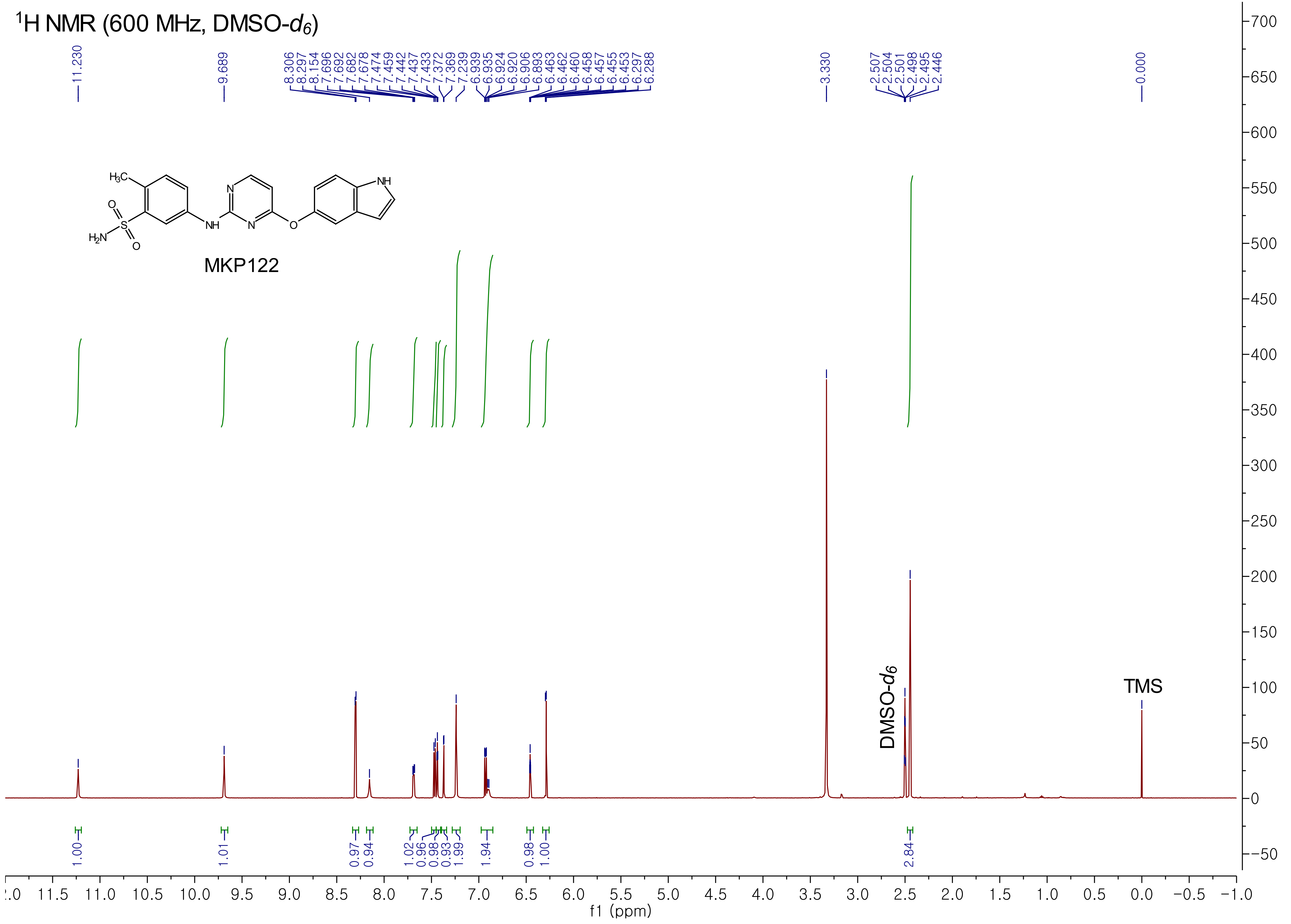

1H NMR (600 MHz, DMSO-d6)
11.230
9.689
8.306 8.297 8.154 7.696 7.692 7.682 7.678 7.474 7.459 7.442 7.437 7.433 7.372 7.369 7.239 6.939 6.935 6.924 6.920 6.906 6.893 6.463 6.462 6.460 6.458 6.457 6.455 6.453 6.297 6.288
3.330
2.507 2.504 2.501 2.498 2.495 2.446
0.000
H3C
O
S
H2N
O
NH
N
N
O
NH
MKP122
DMSO-d6
TMS
1.00
1.01
0.97
0.94
1.02
0.96
0.98
0.93
1.99
1.94
0.98
1.00
2.84
f1 (ppm)

^{13}C NMR (150 MHz, DMSO-d_6)

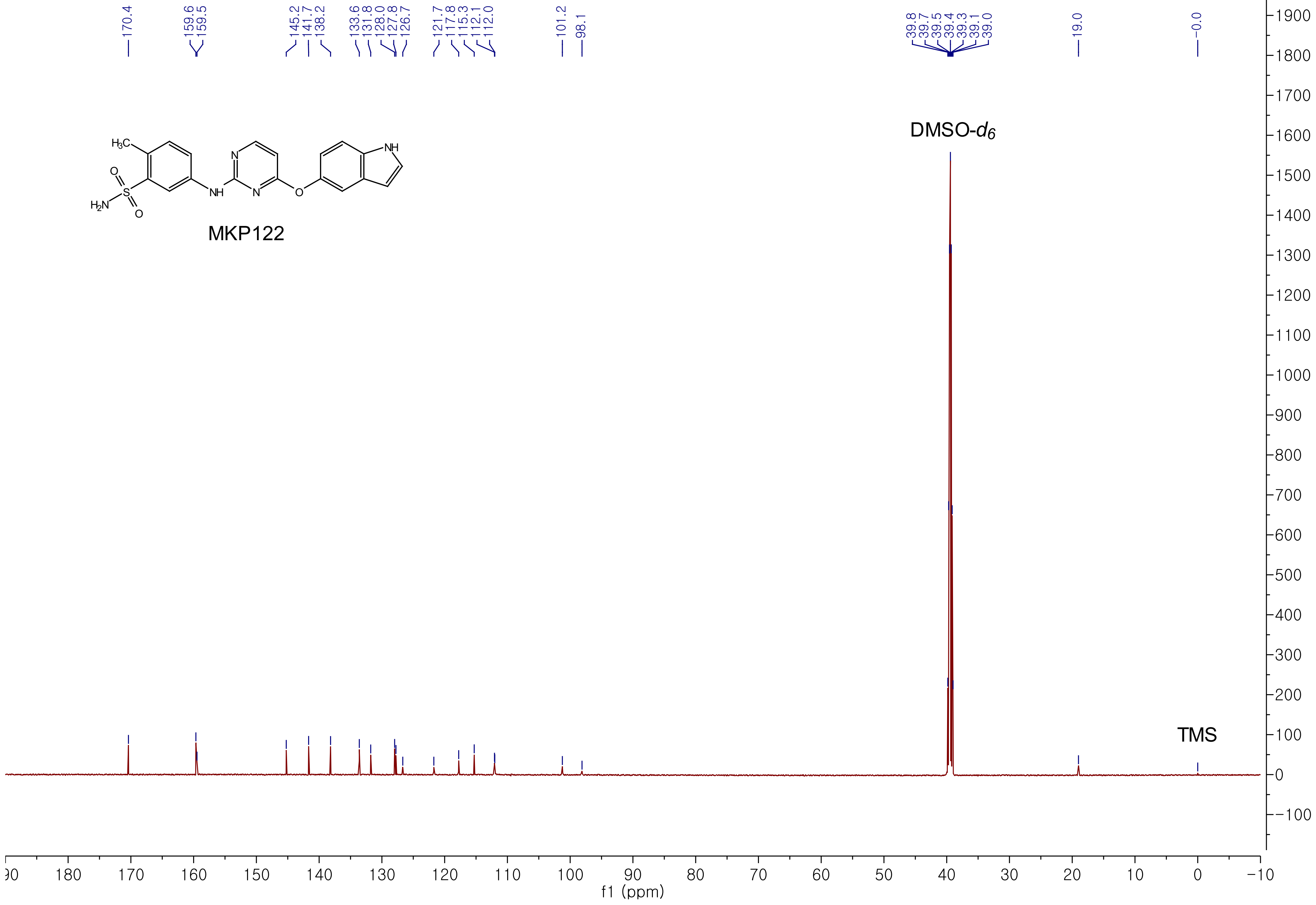

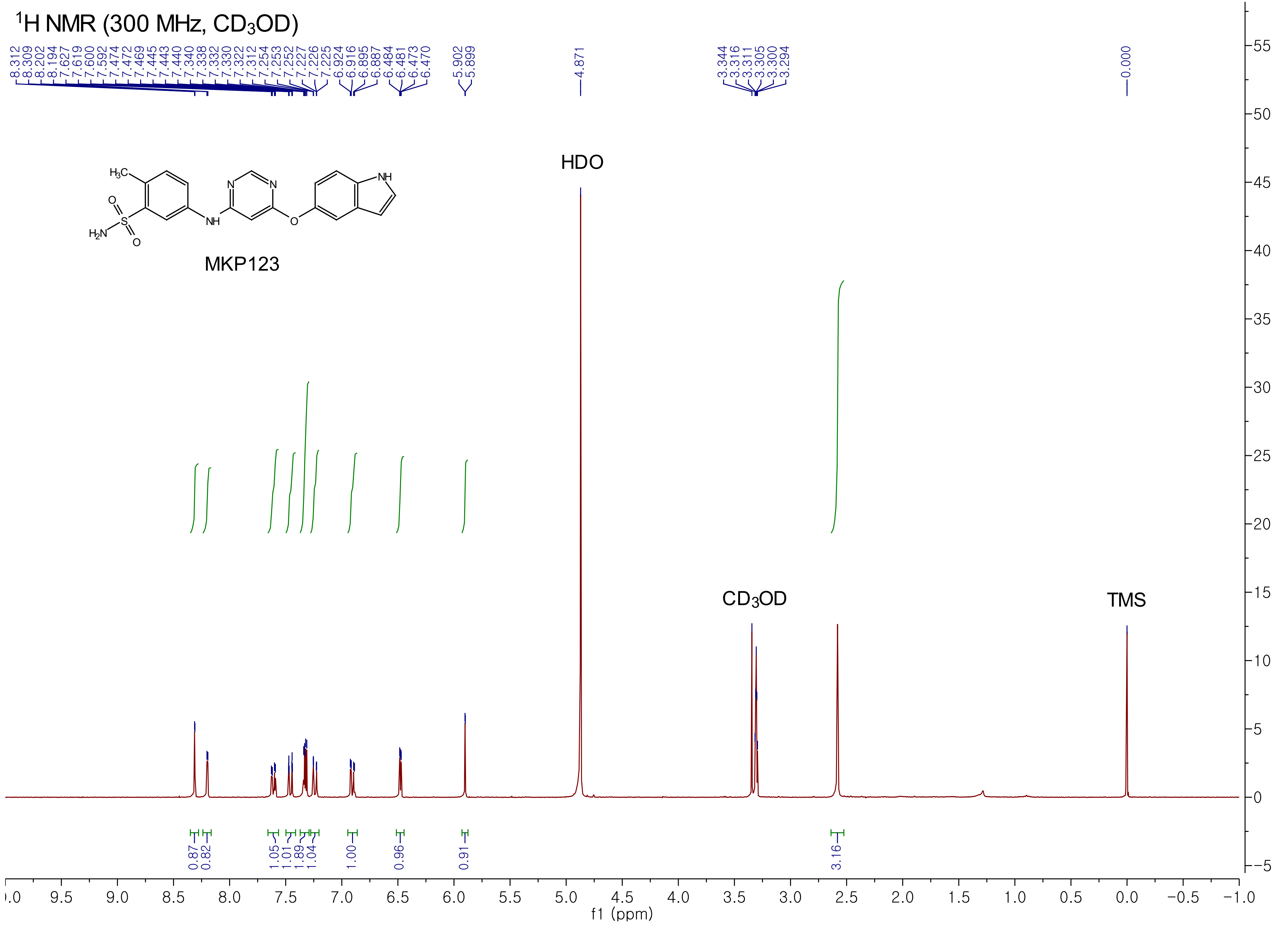

^{1}H NMR (300 MHz, CD_3OD)
8.312 8.309 8.202 8.194 7.627 7.619 7.600 7.592 7.474 7.472 7.469 7.445 7.443 7.440 7.340 7.338 7.332 7.330 7.322 7.312 7.254 7.253 7.252 7.227 7.226 7.225 6.924 6.916 6.895 6.887 6.484 6.481 6.473 6.470
5.902 5.899
4.871
3.344 3.316 3.311 3.305 3.300 3.294
0.000
MKP123
HDO
CD_3OD
TMS
0.87 0.82 1.05 1.01 1.89 1.04 1.00 0.96 0.91 3.16
f1 (ppm)

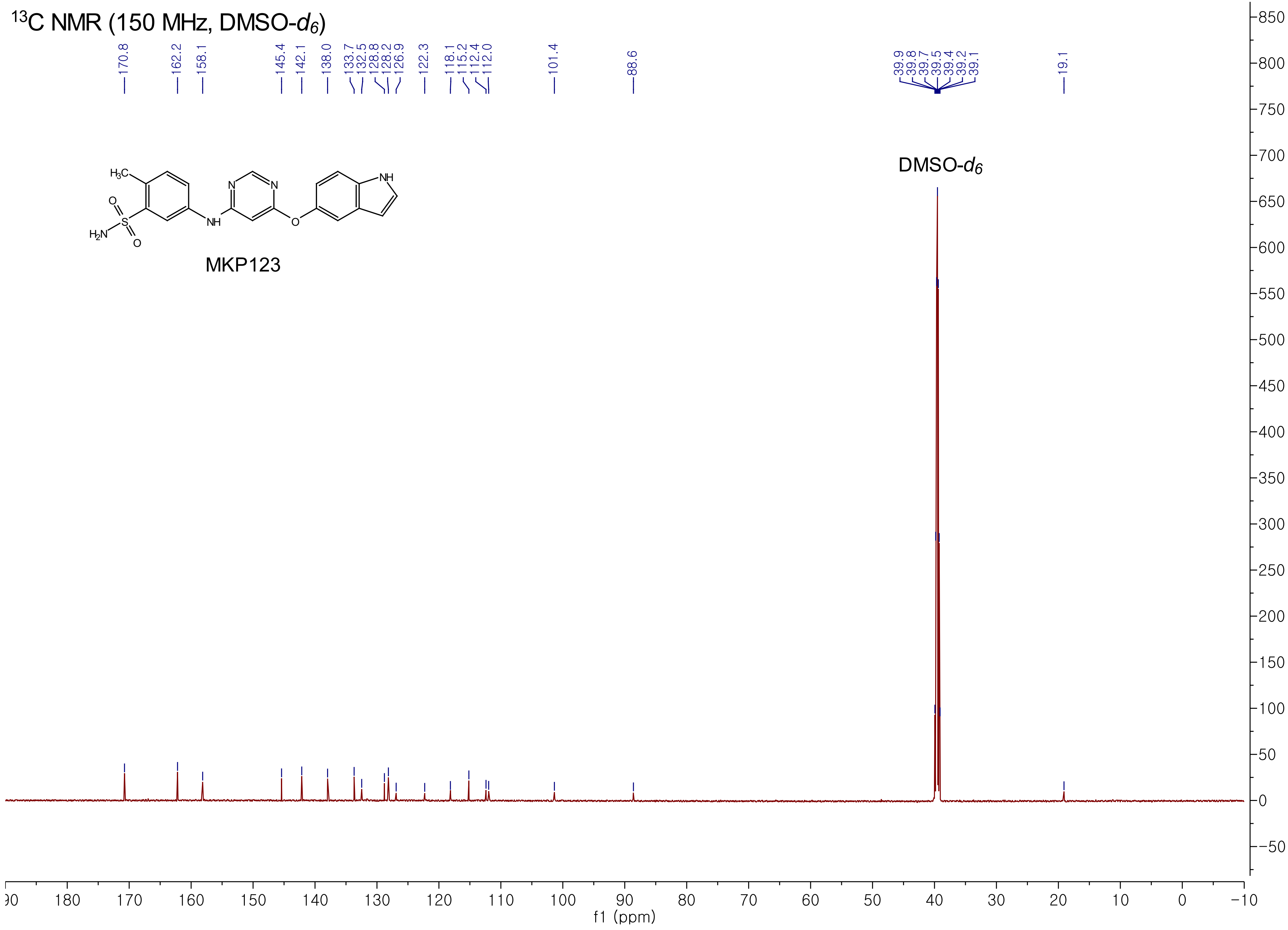

13C NMR (150 MHz, DMSO-d6)
170.8
162.2
158.1
145.4
142.1
138.0
133.7
132.5
128.8
128.2
126.9
122.3
118.1
115.2
112.4
112.0
101.4
88.6
39.9
39.8
39.7
39.5
39.4
39.2
39.1
19.1
DMSO-d6
H3C
O
S
H2N
O
NH
N
N
O
NH
MKP123
f1 (ppm)
